# Correction: The Whole Set of Constitutive Promoters Recognized by RNA Polymerase RpoD Holoenzyme of *Escherichia coli*

**DOI:** 10.1371/journal.pone.0100908

**Published:** 2014-06-16

**Authors:** The PLOS ONE Staff

## Notice of Republication

This article was republished on May 30^th^, 2014 due to the exclusion of Figure 2 from the PDF. Please download this article again to view the corrected version. The originally published, uncorrected article and the republished, corrected article are provided here for reference.

## Supporting Information

File S1 Originally published, uncorrected article.
(PDF)

File S2 Republished corrected article.
(PDF)
