# Supplementary material for: The Whole Set of Constitutive Promoters Recognized by RNA Polymerase RpoD Holoenzyme of Escherichia coli
Source: PLoS One. 2014 Mar 6;9(3):e90447. doi: 10.1371/journal.pone.0090447 (PMC3946193; doi:10.1371/journal.pone.0090447)
Supplement: Table S1 — RNA polymerase RpoD holoenzyme-binding sites on the E. coli genome. A total of 1,075 RpoD holoenzyme-binding sites were identified within spacers on the entire E. coli K-12 W3110 genome. The binding sites identified within intergenic spacers were classified into Type-A, Type-B and Type-C (see Fig. 2 and Table 1). The constitutive promoters were predicted based on the location of the RpoD holoenzyme-binding sites. [S1A] Promoters within type-A spacers. The genes under the constitutive promoters on left- and right-sides of type-A intergenic spacers and their functions are described. The genes shown under filled background encodes DNA-binding transcription factors (TFs), each regulating a set of target genes. The spacers under grey background contain two distinct peaks of holoenzyme binding. The positions of these genes on the genome are described on the left-end column while the levels of SELEX-peaks relative to the highest peak are described on the right-end column. [S1B] Promoters within type-B spacers. The genes under the constitutive promoters on either left- or right-sides of type-B intergenic spacers and their functions are described. The positions of these genes on the genome are described on the left-end column while the levels of SELEX-peaks relative to the highest peak are described on the right-end column. The genes shown under black background encodes DNA-binding TFs. The spacers under grey background contain two distinct peaks of holoenzyme binding. (PDF) [file pone.0090447.s001.pdf]

Supplemental Table S1A

RNA Polymerase RpoD Holoenzyme-Binding Sites on the *E. coli* Genome (Cut-off level, 2.0)

Position	Function	Left Gene	D	RpoD	D	Right Gene	Function	Level
41946	carnitine BCCT transporter	caiT	<		>	fixA	anaerobic carnitine metabolism	0.05
113368	dephospho-CoA kinase	coaE	<		>	guaC	GMP reductase	0.04
141230	glucose dehydrogenase	gcd	<		>	hpt	hypoxanthine-guanine phosphoribosyltransferase	0.23
142654	carbonic anhydrase 2	can	<		>	yadG	predicted ABC transporter	0.04
243432	acyl-CoA dehydrogenase	fadE	<		>	lpcA	D-sedoheptulose 7-phosphate isomerase	0.06
246530	YafQ-DinJ system antitoxin	dinJ	<		>	yafL	lipoprotein and C40 family peptidase	0.11
259470	outer membrane phosphoporin protein E	phoE	<		>	proB	glutamate-5-kinase	0.10
281364	CP4-6 prophage transcriptionl regulator	yagA	<		>	yagE	CP4-6 prophage lyase/synthase	0.04
310970	predicted regulator	matA	<		>	ykgL	predicted protein	0.04
328568	transcription regulator	betI	<		>	betT	BetT choline BCCT transporter	0.36
383958	conserved protein	yaiS	<		>	tauA	taurine transporter	0.07
563962	transcription regulator	fimZ	<		>	argU	tRNA-argU	0.03
607040	carboxylateamine ligase	ybdK	<		>	hokE	small toxic polypeptide	0.10
611830	enterochelin receptor outer membrane receptor	fepA	<		>	fes	enterochelin esterase	0.16
621536	iron-enterobactin transporter	fepD	<		>	entS	EntS MFS transporter	0.23
623866	iron-enterobactin transporter	fepB	<		>	entC	isochorismate synthase 1	0.06
637856	thiol:disulfide interchange protein	dsbG	<		>	ahpC	alkyl hydroperoxide reductase	0.04
702948	glucosamine-6-phosphate deaminase	nagB	<		>	nagE	N-acetyl glucosamine PTS enzyme	0.09
753948	citrate synthase	gltA	<		>	sdhC	succinate dehydrogenase membrane protein	0.04
765072	transcription regulator	mngR	<		>	mngA	2-O-a-mannosyl-D-glycerate PTS enzyme	0.07
812472	hypothetical protein	ybhU	<		>	uvrB	DNA repair excision nuclease	0.10
849568	threonine and homoserine exporter	rhtA	<		>	ompX	outer membrane protein X	0.16
852256	RybA small RNA	rybA	<		>	mntR	transcription regulator	0.17
889170	predicted transporter	ybjL	<		>	ybjM	predicted inner membrane protein	0.17
1020252	SOS cell division inhibitor	sulA	<		>	sxy	transcriptional coactivator for CRP	0.16
1102554	transcription regulator	csgD	<		>	csgB	curlin minor subunit	0.24
1103068	transcription regulator	csgD	<		>	csgB	curlin minor subunit	0.04
1108470	periplasmic glucan succinyl modification protein	mdoC	<		>	mdoG	periplasmic glucan (MDO) biosynthesis protein	0.33
1115832	lauroyl acyltransferase	lpxL	<		>	yceA	conserved protein	0.05
1197570	e14 prophage inner membrane protein	ymfE	<		>	lit	e14 prophage cell death peptidase	0.14
1226764	g-type lysozyme inhibitor	pliG	<		>	ycgL	conserved protein	0.32
1234072	sodium/proton transporter	nhaB	<		>	fadR	transcription regulator	0.03
1236636	conserved protein	ycgB	<		>	dadA	D-amino acid dehydrogenase	0.06
1258038	peptidyl-tRNA hydrolase	pth	<		>	ychH	stress-induced protein	0.12
1292540	transcription regulator	hns	<		>	tdk	thymidine-deoxyuridine kinase	0.04
1297734	[illegible]ohol dehydrogenase/cetaldehyde dehydrogenase	adhE	<		>	ychE	predicted inner membrane protein	0.14
1386966	lipid hydroperoxide peroxidase	tpx	<		>	ycjG	L-Ala-D/L-Glu epimerase	0.07
1389946	predicted hydrolase	ycjY	<		>	ycjZ	transcription regulator	0.06
1440860	D-lactate dehydrogenase	ldhA	<		>	ydbH	predicted protein	0.30
1480954	NADH-azoreductase, FMN-dependent	azoR	<		>	hrpA	ATP-dependent helicase	0.07
1488764	glyceraldehyde 3-phosphate dehydrogenase C	gapC	<		>	cybB	cytochrome b561	0.06
1489562	RydC small RNA	rydC	<		>	ydcA	hypothetical protein	0.04
1493268	transcription regulator	ydcI	<		>	ydcJ	conserved protein	0.06
1507266	predicted protein	yncJ	<		>	hicA	HicAB toxin	0.04
1554348	biofilm-dependent modulation protein	bdm	<		>	osmC	osmotically inducible peroxidase	0.09
1612752	succinate semialdehyde dehydrogenase	sad	<		>	yneJ	transcription regulator	0.12
1616964	predicted transporter	marC	<		>	marR	transcription regulator	0.03
1630740	predicted transporter	ydfJ	<		>	ydfK	Qin prophage transcription regulator	0.14
1717842	anhydro-N-acetylmuramic acid kinase	anmK	<		>	slyB	outer membrane lipoprotein	0.04
1732144	glutaredoxin 4	grxD	<		>	ydhO	predicted lipoprotein	0.05
1737846	transcriptiol regulator	ydhB	<		>	ydhC	YdhC drug MFS transporter	0.04
1753332	predicted protein	ydhZ	<		>	pykF	pyruvate kinase I monomer	0.06
1800650	threonyl-tRNA synthetase	thrS	<		>	arpB	predicted protein	0.04
1908132	transcriptio regulator	kdgR	<		>	yebQ	predicted transporter	0.18
1921040	protein phosphatase 1	pphA	<		>	ryeA	RyeA small RNA	0.05
1944202	small protein	yobI	<		>	yebB	predicted protein	0.04
1948750	aspartyl-tRNA synthetase	aspS	<		>	yecD	predicted hydrolase	0.07
2006256	predicted protein	yedD	<		>	yedE	predicted inner membrane protein	0.04
2023330	DsrA RNA	dsrA	<		>	yedP	predicted phosphatase	0.04
2036832	transcription regulator	yedW	<		>	yedX	conserved protein	0.14
2065860	IS5 transposase and trans-activator	insH-6	<		>	yoeA	CP4-44 prophage hemin or colicin receptor	0.05
2066688	IS5 transposase and trans-activator	insH-6	<		>	yoeA	CP4-44 prophage hemin or colicin receptor	0.18

2087740	YoeB-YefM toxin transcription regualator	yefM	<		>	hisL	*his* operon leader peptide	0.30
2228572	tRNA dihydrouridine synthase	dusC	<		>	yohJ	conserved inner membrane protein	0.12
2241932	GTP cyclohydrolase I monomer	folE	<		>	yeiG	S-lactoylglutathione hydrolase/S-formylglutathione	0.05
2247764	transcription regulator	yeiE	<		>	yeiH	conserved inner membrane protein	0.06
2301638	ferredoxin-type protein	napF	<		>	yojO	hypothetical protein	0.06
2310860	outer membrane protein OmpC	ompC	<		>	micF	MicF antisense RNA	0.07
2362346	outer membrane protein	yfaZ	<		>	nudI	pyrimidine deoxynucleoside triphosphate pyrophos	0.04
2404652	transcription regulator	lrhA	<		>	alaA	glutamate-pyruvate aminotransferase	0.08
2405036	transcription regulator	lrhA	<		>	alaA	glutamate-pyruvate aminotransferase	0.53
2411432	conserved inner membrane protein	yfbV	<		>	ackA	propionate kinase/acetate kinase	0.04
2481772	EmrK putative membrane fusion protein	emrK	<		>	evgA	transcription regulator	0.45
2493362	predicted inner membrane protein	yfdY	<		>	lpxP	palmitoleoyl acyltransferase	0.17
2507630	glucokinase	glk	<		>	yfeO	predicted ion channel protein	0.06
2510752	manganese ion NRAMP transporter	mntH	<		>	nupC	nucleoside NUP transporter	0.21
2524952	transcription regulator	yfeR	<		>	yfeH	putative cytochrome oxidase	0.04
2550354	predicted acyltransferase	ypeA	<		>	amiA	N-acetylmuramoyl-L-alanine amidase 1	0.86
2583536	putative oxidoreductaset	aegA	<		>	narQ	NarPQ TCS sensory histidine kinase	0.05
2589272	small protein	ypfM	<		>	yffB	conserved protein	0.10
2597942	dihydrodipicolinate synthase	dapA	<		>	gcvR	transcription regulator	0.03
2618872	uracil phosphoribosyltransferase	upp	<		>	purM	phosphoribosylaminoimidazole synthetase	0.09
2632266	IMP dehydrogenase	guaB	<		>	xseA	exonuclease VII	0.10
2661336	tRNA:Cm32/Um32 methyltransferase	trmJ	<		>	suhB	inositol monophosphatase	0.26
2683870	serine hydroxymethyltransferase	glyA	<		>	hmp	NO dioxygenase/dihydropteridine reductase 2	0.19
2708460	RNA polymerase, sigma E factor	rpoE	<		>	nadB	L-aspartate oxidase	0.18
2752830	toxin of a putative toxin-antitoxin pair	ratA	<		>	smpB	small protein B	0.26
2784038	tRNA-Ile	ileY	<		>	ygaQ_1	predicted protein	0.22
2795244	predicted membrane protein	yqaE	<		>	ygaV	transcription regulator	0.17
2823668	membrane-bound lytic murein transglycosylase B	mltB	<		>	srlA	glucitol/sorbitol-specific PTS IIC	0.07
2859462	DNA-binding transcriptional regulator, DEOR-type	ygbI	<		>	ygbJ	predicted NAD(P)-binding dehydrogenase	0.05
2890256	sulfite reductase, flavoprotein subunit	cysJ	<		>	queD	6-carboxy-5,6,7,8-tetrahydropterin synthase	0.04
2903434	conserved protein	queE	<		>	yqcG	envelope stress response small protein	0.25
2977048	diaminopimelate decarboxylase	lysA	<		>	lysR	transcription regulator	0.04
3053570	predicted protein	ygfB	<		>	zapA	cytokinetic ring protein	0.16
3126270	glycolate oxidase	glcD	<		>	glcC	transcription regulator	0.06
3190146	predicted glycogen synthesis protein	glgS	<		>	yqiJ	putative oxidoreductase	0.17
3214730	predicted siderophore interacting protein	yqjH	<		>	yqjI	transcription regulator	0.33
3217532	aerotaxis sensor receptor, flavoprotein	aer	<		>	ygjG	putrescine aminotransferase/diamine transaminas	0.20
3338068	phospholipid ABC transporter	mlaF	<		>	yrbG	YrbG CaCA transporter	0.05
3411666	transcription regulator	envR	<		>	acrE	transmembrane protein	0.04
3431738	conserved protein	smf	<		>	def	peptide deformylase	0.15
3559934	thiosulfate sulfurtransferase	glpE	<		>	glpD	glycerol 3-phosphate dehydrogenase	0.69
3635668	FAD/NAD(P)-bindingoxidoreductase	yhiN	<		>	pitA	PitA phosphate transporter	0.07
3646262	damage-inducible protein	dinQ	<		>	arsR	transcription regulator	0.12
3651236	IS5 transposase and trans-activator	insH	<		>	slp	starvation lipoprotein	0.14
3651858	IS5 transposase and trans-activator	insH	<		>	slp	starvation lipoprotein	0.14
3735330	conserved protein	bax	<		>	malS	alpha-amylase	0.18
3769870	predicted inner membrane protein	yibI	<		>	mtlA	mannitol PTS permease	0.04
3806148	lipopolysaccharide core heptosyl transferase III	waaQ	<		>	waaA	KDO transferase	0.11
3819438	DNA ligase	ligB	<		>	gmk	deoxyguanylate/guanylate kinase	0.20
3834134	GPH transporter	yicJ	<		>	selC	tRNA-sec (selenocycteine)	0.04
3851352	IstR-2 RNA	istR-2	<		>	tisA	phantom gene	0.74
3925170	transcription regulator	asnC	<		>	asnA	asparagine synthetase	0.12
3948344	predicted ATP-dependent protease	yifB	<		>	ilvL	*ilvGEDA* operon leader peptide	0.04
3984454	arylsulfatase	aslA	<		>	glmZ	GlmZ small RNA	0.98
3988832	hydroxymethylbilane synthase	hemC	<		>	cyaA	adenylate cyclase	0.09
4083972	formate dehydrogenase-Ot	fdoG	<		>	fdhD	affects formate dehydrogenase-N	0.22
4103830	transcription regulator	cpxR	<		>	cpxP	Cpx response regulator	0.06
4116232	glycerol MIP channel	glpF	<		>	zapB	cell division factor ZapB	0.28
4124862	primosome factor N'	priA	<		>	rpmE	50S ribosomal subunit protein L31	0.03
4126572	transcription regulator	metJ	<		>	metB	O-succinylhomoserine lyase	0.17
4228166	dipeptidase E	pepE	<		>	rluF	23S rRNA pseudouridine 2604 synthase	0.03
4231546	aspartate aminotransferase	lysC	<		>	pgi	6-phosphogluconolactonase	0.06
4262332	quinone oxidoreductase	qor	<		>	dnaB	replicative DNA helicase	0.06
4272050	excision nuclease	uvrA	<		>	ssb	ssDNA-binding protein	0.67
4273156	predicted inner membrane protein	yjcB	<		>	yjcC	c-di-GMP-specific phosphodiesterase	0.11
4368630	APC transporter	yjeH	<		>	groS	GroES chaperone	0.12
4417636	L-ascorbate 6-phosphate lactonase	ulaG	<		>	ulaA	L-ascorbate PTS permease	0.16

4422860	predicted protein	***yjfY***	<		>	***rpsF***	30S ribosomal subunit protein S6	0.07
4432132	NAD(P)H:quinone oxidoreductase	***ytfG***	<		>	***ytfH***	transcription regulator	0.25
4472066	predicted NAD(P)-binding oxidoreductase	***bdcA***	<		>	***bdcR***	transcription regulator	0.31
4476464	ornithine carbamoyltransferase	***argI***	<		>	***rraB***	ribonuclease E inhibitor protein B	0.07
4477556	predicted acetyltransferase	***yjgM***	<		>	***yjgN***	conserved inner membrane protein	0.14
4502056	IS1 predicted transposase	***insG***	<		>	***yjhB***	YjhB MFS transporter	0.07
4538050	N-acetylneuraminic acid outer membrane channel	***nanC***	<		>	***fimB***	*fim* promoter switch	0.04
4538758	N-acetylneuraminic acid outer membrane channel	***nanC***	<		>	***fimB***	*fim* promoter switch	0.32
4569666	transcription regulator	***yjiR***	<		>	***yjiS***	conserved protein	0.03
4601330	predicted inner membrane protein	***yjjP***	<		>	***yjjQ***	transcription regulator	0.04
4603830	ferrioxamine B iron utilization	***fhuF***	<		>	***yjjZ***	predicted protein	0.20
4633370	transcription regulator	***rob***	<		>	***creA***	conserved protein	0.03
4638418	transcription regulator	***arcA***	<		>	***yjjY***	predicted protein	0.42

142

Supplemental Table S1B

RNA Polymerase RpoD Holoenzyme-Binding Sites on the *E. coli* Genome (Cut-off level, 2.0)

Position	Function	*Left Gene*	D	RpoD	D	*Right Gene*	Function	Level
19830	*InsAB* transposase	***insAB-1***	<		<	***insB-1***		0.17
127644		***aceF***	>		>	***lpd***	lipoamide dehydrogenase	0.04
164658		***hrpB***	>		>	***mrcB***	fused glycosyl transferase and transpeptidase	0.05
167354		***mrcB***	>		>	***fhuA***	colicin M receptor outer membrane protein	0.46
176542		***clcA***	>		>	***erpA***	essential respiratory protein A	0.39
184258	conserved protein	***yaeH***	<		<	***yaeI***		0.06
268354	CP4-6 prophage GTP-binding protein	***ykfA***	<		<	***perR***		0.06
289640	ornithine carbamoyltransferase 2	***argF***	<		<	***insAB-3***		0.16
292358	CP4-6 prophage conserved protein	***yagK***	<		<	***yagL***		0.93
331130		***betT***	>		>	***yahA***	c-di-GMP phosphodiesterase	0.05
379186	transcription regulator	***frmR***	<		<	***yaiO***		0.28
447242	cytochrome *bo* terminal oxidase	***cyoD***	<		<	***cyoC***		0.12
454246		***bolA***	>		>	***tig***	cell division trigger factor	0.10
455840		***tig***	>		>	***clpP***	ATP-dependent serine protease	0.08
467530		***cof***	>		>	***ybaO***	transcription regulator	0.28
477932	conserved inner membrane protein	***ylaB***	<		<	***ylaC***		0.06
478570	predicted inner membrane protein	***ylaC***	<		<	***maa***		0.05
479130	maltose O-acetyltransferase	***maa***	<		<	***hha***		0.04
522060		***ybbP***	>		>	***rhsD***	*rhs* element protein	0.06
522472		***ybbP***	>		>	***rhsD***	*ths* element protein	0.19
570042		***ybcK***	>		>	***ybcL***	DLP12 prophage kinase inhibitor	0.05
585168	outer membrane protease VII	***ompT***	<		<	***envY***		0.19
592452	phage N4 receptor inner membrane protein	***nfrB***	<		<	***cusS***		0.03
604658	dihydropteridine reductase monomer	***nfsB***	<		<	***ybdF***		0.05
629042		***entH***	>		>	***cstA***	carbon starvation peptide transporter	0.05
656450		***pagP***	>		>	***cspE***	transcription antiterminator and RNA stability regul	0.09
659648	lipoate synthase monomer	***lipA***	<		<	***ybeF***		0.07
660756	transcription regulator	***ybeF***	<		<	***lipB***		0.07
710068	RyhB-regulated *fur* leader peptide	***uof***	<		<	***fldA***		0.16
710768	flavodoxin 1	***fldA***	<		<	***ybfE***		0.16
711270	LexA-regulated protein	***ybfE***	<		<	***ybfF***		0.07
733362		***ybfB***	>		>	***ybfO***	Rhs-like conserved protein	0.03
752436	fimbrial-like adhesin protein	***ybgD***	<		<	***gltA***		0.07
770038		***mngB***	>		>	***cydA***	cytochrome bd-I terminal oxidase	0.13
770668		***mngB***	>		>	***cydA***	cytochrome bd-I terminal oxidase	0.15
786938	phosphoglyceromutase 1 monomer	***gpmA***	<		<	***galM***		0.38
791446	UDP-glucose 4-epimerase monomer	***galE***	<		<	***modF***		0.12
837732	conserved protein	***ybiI***	<		<	***ybiX***		0.04
891170		***nfsA***	>		>	***rimK***	ribosomal protein S6 modification protein	0.05
899960	arginine binding periplasmic protein	***artJ***	<		<	***artM***		0.04
903064	arginine transporter	***artP***	<		<	***ybjP***		0.10
914352	predicted transporter	***ybjE***	<		<	***aqpZ***		0.10
938542		***rarA***	>		>	***serS***	seryl-tRNA synthetase	0.05
959450		***aroA***	>		>	***ycaL***	putative heat shock protein	0.05
1019652	outer membrane protein 3a	***ompA***	<		<	***sulA***		0.03
1043864	phosphotyrosine-protein phosphatase	***etp***	<		<	***gfcE***		0.03
1084156		***efeB***	>		>	***phoH***	phosphate starvation-induced ATP-binding protein	0.19
1120230	biofilm formation regulator	***bssS***	<		<	***dinI***		0.63
1133766		***flgF***	>		>	***flgG***	flagellar basal-body rod protein	0.03
1150750		***fabG***	>		>	***acpP***	apo-[acyl carrier protein]	0.09
1152532		***fabF***	>		>	***pabC***	para-aminobenzoate synthase	0.03
1165242		***ycfP***	>		>	***ndh***	NADH:ubiquinone oxidoreductase II	0.20
1173266	transcription-repair coupling factor	***mfd***	<		<	***ycfT***		0.05
1195654		***icd***	>		>	***C0293***	C0293 small RNA	0.11
1209454		***pinE***	>		>	***mcrA***	restriction of DNA at 5-methylcytosine residues	0.08
1213264	tanscription regulator	***ycgE***	<		<	***ycgF***		0.23
1216544		***ymgC***	>		>	***ycgG***	conserved inner membrane protein	0.04
1218568		***ymgF***	>		>	***ycgH***	autotransporter	0.06
1218960		***ymgF***	>		>	***ycgH***	autotransporter	0.15
1219948		***ymgF***	>		>	***ycgH***	autotransporter	0.04
1229964		***C0299***	>		>	***umuD***	mutagenesis and error-prone repair protein	0.05
1257172	putative GTP-binding protein	***ychF***	<		<	***pth***		0.07

1285738		***narI***	>		>	***ychS***	predicted protein	0.09
1298670		***ychE***	>		>	***oppA***	oligopeptide ABC transporter	0.25
1320956	anthranilate synthase	***trpE***	<		<	***trpL***		0.05
1322756		***yciO***	>		>	***yciQ***	predicted inner membrane protein	0.04
1331866		***topA***	>		>	***cysB***	transcriptional regulator	0.13
1338150		***pgpB***	>		>	***yciS***	conserved inner membrane protein	0.06
1341546	osmotically inducible lipoprotein	***osmB***	<		<	***yciT***		0.09
1349156	enoyl-[acyl-carrier-protein] reductase	***fabI***	<		<	***ycjD***		0.42
1349842	conserved protein	***ycjD***	<		<	***sapF***		0.04
1355134	ABC peptide transporter	***sapA***	<		<	***ymjA***		0.07
1432638	Rac prophage transcription regulator	***ynaE***	<		<	***uspF***		0.20
1453172		***paaB***	>		>	***paaC***	phenylacetyl-CoA monooxygenase	0.04
1500460		***tehB***	>		>	***ydcL***	predicted lipoprotein	0.06
1525368		***yncH***	>		>	***rhsE***	RhsE protein	0.05
1529370		***yncI***	>		>	***yncM***	predicted protein	0.06
1584860	acid resistance protein	***ydeP***	<		<	***ydeQ***		0.05
1590548	HipAB toxin	***hipB***	<		<	***ydeU***		0.19
1596530	predicted lipoprotein	***ydeK***	<		<	***lsrK***		0.05
1639234	Qin prophage S lysis protein	***essQ***	<		<	***cspB***		0.07
1647456		***dicF***	>		>	***dicB***	Qin prophage cell division inhibition protein	0.04
1651956	Zn-depd and NAD(P)-binding oxidoreductase,	***rspB***	<		<	***rspA***		0.12
1653236	mannonate/altronate dehydratase	***rspA***	<		<	***ynfA***		0.04
1669136		***ynfM***	>		>	***asr***	acid shock protein	0.03
1682244		***rstB***	>		>	***tus***	replication inhibition Ter-site protein	0.18
1733430		***ydhO***	>		>	***sodB***	superoxide dismutase (Fe)	0.05
1739130		***ydhC***	>		>	***cfa***	cyclopropane fatty acyl phospholipid synthase	0.24
1762758	Fe-S cluster scaffold protein	***sufA***	<		<	***rydB***		0.03
1763230	predicted protein	***ydiH***	<		<	***ydiI***		0.14
1768530		***rprA***	>		>	***ydiL***	conserved protein	0.09
1787570		***aroH***	>		>	***ydiE***	conserved protein	0.20
1808170		***yniC***	>		>	***ydjM***	LexA-regulated inner membrane protein	0.29
1808932		***ydjM***	>		>	***ydjN***	predicted transporter	0.09
1811360	predicted protein	***ydjO***	<		<	***cedA***		0.25
1821530		***nadE***	>		>	***cho***	excision repair endonuclease	0.09
1823166	CpsA-related protein	***ves***	<		<	***spy***		0.03
1852958	predicted transcriptional regulator	***ydjF***	<		<	***ydjG***		0.13
1870038		***yeaI***	>		>	***yeaJ***	predicted diguanylate cyclase	0.04
1877272	conserved inner membrane protein	***yeaQ***	<		<	***yoaG***		0.05
1887966	fatty acyl-CoA synthetase	***fadD***	<		<	***yeaY***		0.05
1905768	stress response small protein	***yobF***	<		<	***yebO***		0.13
1906230	stress response small protein	***yobF***	<		<	***yebO***		0.07
1912840	tail-specific protease	***prc***	<		<	***proQ***		0.04
1923454		***holE***	>		>	***yobB***	conserved protein	0.05
1927864	conserved inner membrane protein	***yebE***	<		<	***yebF***		0.31
1932868	phosphogluconate dehydratase	***edd***	<		<	***zwf***		0.11
1956436	trimethylamine N-oxide reductase III	***torY***	<		<	***cutC***		0.10
1990832	phosphatidylglycerophosphate synthase	***pgsA***	<		<	***uvrC***		0.05
1994970	transcription regulator	***sdiA***	<		<	***yecC***		0.10
1999044	regulator of sigma-S activity	***fliZ***	<		<	***fliA***		0.04
2039332		***yedZ***	>		>	***zinT***	cadmium-induced cadmium binding protein	0.26
2054646		***amn***	>		>	***yeeN***	conserved protein	0.20
2063954	cobinamide kinase	***cobU***	<		<	***insH-6***		0.18
2069334		***yeeP***	>		>	***isrC***	IsrC small RNA	0.27
2077562	conserved protein	***yeeX***	<		<	***yeeA***		0.12
2111266	dTDP-glucose 4,6-dehydratase	***rfbB***	<		<	***galF***		0.08
2163042		***baeR***	>		>	***yegP***	predicted protein	0.07
2165566	P2 prophage transcription regulator	***ogrK***	<		<	***yegZ***		0.19
2181734	predicted hydrolase	***yegX***	<		<	***thiD***		0.05
2210264	conserved protein	***yehS***	<		<	***yehT***		0.14
2222972	penicillin-binding protein 7	***pbpG***	<		<	***yohC***		0.07
2276340	predicted protein	***yejG***	<		<	***bcr***		0.68
2284170		***yejM***	>		>	***proL***	Pro-tRNA	0.14
2304772	malate:quinone oxidoreductase	***mqo***	<		<	***yojI***		0.11
2342332	adhesin	***yfaL***	<		<	***ypaB***		0.05
2371636	polymyxin B resistance protein	***pmrD***	<		<	***menE***		0.05
2403250	NADH:ubiquinone oxidoreductase	***nuoA***	<		<	***lrhA***		0.03
2403752	NADH:ubiquinone oxidoreductase	***nuoA***	<		<	***lrhA***		0.04

2424846	histidine/lysine/arginine/ornithine transporter	***hisJ***	<		<	***argT***		0.08
2454170	predicted fimbrial-like adhesin protein	***yfcV***	<		<	***sixA***		0.11
2488236	YfdV AEC transporter	***yfdV***	<		<	***oxc***		0.04
2491738	formyl-CoA transferase monomer	***frc***	<		<	***yfdX***		0.03
2530446		***cysZ***	>		>	***cysK***	cysteine synthase A	0.04
2561330		***yffP***	>		>	***yffQ***	CPZ-55 prophage protein	0.07
2562468		***yffR***	>		>	***yffS***	CPZ-55 prophage protein	0.07
2599140		***bcp***	>		>	***hyfA***	hydrogenase 4	0.31
2618272	UraA uracil NCS2 transporter	***uraA***	<		<	***upp***		0.03
2654368	aminopeptidase B	***pepB***	<		<	***iscX***		0.03
2689364	GlmY small RNA	***glmY***	<		<	***purL***		0.23
2698136	RyfB small RNA	***ryfB***	<		<	***shoB***		0.08
2723936	alpha-ketoglutarate MFS transporter	***kgtP***	<		<	***rrfG***		0.27
2749768		***nadK***	>		>	***recN***	recombination and DNA repair protein	0.22
2753630		***smpB***	>		>	***ssrA***	tmRNA, 10Sa RNA	1.00
2754072		***ssrA***	>		>	***intA***	CP4-57 prophage integrase	0.03
2763458	CP4-57 prophage protein	***yfjL***	<		<	***yfjM***		0.34
2765650		***yfjO***	>		>	***yfjP***	CP4-57 GTP-binding protein	0.04
2773166		***yfjW***	>		>	***yfjX***	CP4-57 antirestriction protein	0.35
2805072		***proW***	>		>	***proX***	glycine betaine transporter	0.07
2807634		***ygaY***	>		>	***ygaZ***	YgaZ L-valine LIV-E exporter	0.19
2826636		***srlD***	>		>	***gutM***	transcription regulator	0.07
2845448	hydrogenase 3t	***hycD***	<		<	***hycC***		0.09
2857732		***mutS***	>		>	***pphB***	protein Tyr-phosphatase	0.20
2877772	predicted integrase	***cas1***	<		<	***casE***		0.04
2909368	MazE antitoxin	***chpR***	<		<	***relA***		0.50
2967172	RNA pyrophosphohydrolase	***rppH***	<		<	***ygdT***		0.46
2982230	predicted 5-keto 4-deoxyuronate isomerase	***kduI***	<		<	***yqeF***		0.18
2989046		***ygeF***	>		>	***ygeG***	predicted chaperone	0.05
2991858		***ygeI***	>		>	***pbl***	predicted peptidoglycan-binding enzyme	0.04
3044072	predicted NAD(P)-binding oxidoreductase	***ygfF***	<		<	***gcvP***		0.04
3054032		***zapA***	>		>	***ssrS***	6S RNA	0.12
3054846		***ygfA***	>		>	***sibC***	SibC small RNA	0.09
3071934	erythrose 4-phosphate dehydrogenase	***epd***	<		<	***yggC***		0.05
3112370	prepilin peptidase	***pppA***	<		<	***yghJ***		0.03
3151570		***metC***	>		>	***yghB***	conserved inner membrane protein	0.11
3166762	MqsR toxin mRNA interferase	***mqsR***	<		<	***ygiV***		0.24
3167230	transcription regulator	***ygiV***	<		<	***ygiW***		0.04
3174034	esterase	***yqiA***	<		<	***cpdA***		0.07
3183170		***yqiC***	>		>	***ygiL***	predicted fimbrial-like adhesin protein	0.05
3205972		***ttdB***	>		>	***ttdT***	TtdT tartrate/succinate DASS transporter	0.04
3220450		***ebgR***	>		>	***ebgA***	evolved beta-D-galactosidase	0.05
3226938		***ygjJ***	>		>	***ygjK***	glycoside hydrolase	0.07
3237654		***alx***	>		>	***sstT***	SstT DAACS transporter	0.14
3244640		***exuT***	>		>	***exuR***	transcription regulator	0.12
3245772		***exuR***	>		>	***yqjA***	conserved inner membrane protein	0.22
3265634		***tdcR***	>		>	***yhaB***	predicted protein	0.06
3270768	tartronate semialdehyde reductase	***garR***	<		<	***garL***		0.03
3332864		***ispB***	>		>	***sfsB***	transcription regulator	0.16
3375742	stringent starvation protein'	***sspA***	<		<	***rpsI***		0.59
3383544		***argR***	>		>	***yhcN***	stress-induced protein	0.13
3408230		***prmA***	>		>	***dusB***	tRNA dihydrouridine synthase	0.14
3465030	bacterioferritin-associated ferredoxin	***bfd***	<		<	***chiA***		0.07
3474542	transcription regulator	***yheO***	<		<	***fkpA***		0.05
3481234		***yheS***	>		>	***yheT***	predicted hydrolase	0.07
3497644		***yhfL***	>		>	***frlA***	predicted fructoselysine transporter	0.12
3538170		***yhgF***	>		>	***feoA***	ferrous iron transport protein A	0.05
3576742	transcription regulator	***gntR***	<		<	***yhhW***		0.39
3598870	RNA polymerase, sigma 32 (sigma H) factor	***rpoH***	<		<	***ftsX***		0.03
3628772	predicted HlyD family secretion protein	***yhiI***	<		<	***yhiJ***		0.51
3630862	predicted protein	***yhiJ***	<		<	***yhiK***		0.45
3648872		***arsC***	>		>	***yhiS***	predicted protein	0.59
3654348	acid stress chaperone	***hdeB***	<		<	***hdeA***		0.06
3702772	dipeptide ABC transporter	***dppC***	<		<	***dppB***		0.05
3741746		***yiaK***	>		>	***yiaL***	conserved protein	0.04
3752670	conserved inner membrane protein	***yiaW***	<		<	***aldB***		0.14
3794944		***waaC***	>		>	***rfaL***	O-antigen ligase	0.17

3815652		***yicC***	>		>	***dinD***	DNA-damage-inducible protein	0.08
3906432	phosphate ABC transporter	***pstB***	<		<	***pstA***		0.04
3957944	peptidyl-prolyl cis-trans isomerase C	***ppiC***	<		<	***yifO***		0.28
4047838		***polA***	>		>	***spf***	Spot42 RNA	0.04
4054334	NtrB sensory kinase	***glnL***	<		<	***glnA***		0.10
4058260		***typA***	>		>	***yihL***	putative transcriptional regulator	0.04
4105564		***fieF***	>		>	***pfkA***	6-phosphofructokinase-1 monomer	0.04
4117434	ribonuclease E inhibitor protein	***rraA***	<		<	***menA***		0.12
4179270		***rplL***	>		>	***rpoB***	RNA polymerase, beta-subunit	0.04
4188758	SroH small RNA	***sroH***	<		<	***thiH***		0.04
4281230	conserved protein	***yjcF***	<		<	***actP***		0.32
4292136		***nrfG***	>		>	***gltP***	GltP glutamate/aspartate DAACS transporter	0.05
4295170	conserved protein	***yjcO***	<		<	***fdhF***		0.05
4323832	conserved protein	***yjdN***	<		<	***yjdM***		0.03
4338634	arginine decarboxylase	***adiA***	<		<	***melR***		0.16
4358254	CadB cadaverine/lysine APC exchanger	***cadB***	<		<	***cadC***		0.06
4364770	DcuA dicarboxylate Dcu transporter	***dcuA***	<		<	***aspA***		0.06
4372468	predicted protein	***yjeJ***	<		<	***yjeK***		0.16
4374836		***ecnB***	>		>	***sugE***	SugE SMR transporter	0.13
4402644		***yjeT***	>		>	***purA***	adenylosuccinate synthetase	0.05
4408030		***rlmB***	>		>	***yjfI***	conserved protein	0.06
4424154		***rpsR***	>		>	***rplI***	50S ribosomal subunit protein L9	0.07
4427854		***fklB***	>		>	***cycA***	CycA serine/alanine/glycine APC transporter	0.03
4460968	ribonucleoside-triphosphate reductase	***nrdD***	<		<	***treC***		0.10
4498432	KpLE2 predicted protein	***yjgX_2***	<		<	***yjgX_1***		0.23
4518430	KpLE2 prophage transcription regulator	***yjhU***	<		<	***yjhF***		0.07
4523966	KpLE2 prophage transcription regulator	***yjhI***	<		<	***sgcR***		0.05
4530330	KpLE2 predicted endoglucanase	***sgcX***	<		<	***yjhP***		0.08
4539934		***fimB***	>		>	***fimE***	regulator for fimA	0.08
4540968		***fimE***	>		>	***fimA***	major type 1 subunit fimbrin (pilin)	0.04
4552370		***uxuB***	>		>	***uxuR***	transcription regulator	0.23
4557562	isoaspartyl dipeptidase	***iadA***	<		<	***yjiG***		0.06
4561866	conserved protein	***yjiK***	<		<	***yjiL***		0.05
4570370		***yjiS***	>		>	***yjiT***	conserved protein	0.03
4577430	5-methylcytosine restriction system	***mcrB***	<		<	***symE***		0.52
4617638		***deoA***	>		>	***deoB***	phosphopentomutase	0.04
4619734		***deoD***	>		>	***yjjJ***	transcription regulator	0.05
