# Supplementary material for: The Whole Set of Constitutive Promoters Recognized by RNA Polymerase RpoD Holoenzyme of Escherichia coli
Source: PLoS One. 2014 Mar 6;9(3):e90447. doi: 10.1371/journal.pone.0090447 (PMC3946193; doi:10.1371/journal.pone.0090447)
Supplement: Table S2 — Operons under the control of the constitutive promoters. Operons under the control of constitutive promoters are listed on the operon columns. Promoters shown by shaded background represent those listed in RegulonDB and EcoCyc databases. Spacers containing H-NS binding sites are indicated by HNS. [S2A] Operons under the control of constitutive promoters within type-A spacers. The constitutive promoters identified within type-A spacers direct bidirectional transcription of the operons indicated on left- or right-operon columns. The map positions of the promoter-proximal genes are indicated on the left- and right-end columns. [S2B] Operons under the control of constitutive promoters within type-B spacers. The constitutive promoters identified within type-B spacers direct transcription toward one direction. (PDF) [file pone.0090447.s002.pdf]

Supplemental Table S2A
Constitutive Promoters within Type-A Spacers

Map	Operon	Left Gene	D	P	HNS	P	D	Right Gene	Operon	Map
0.87	*caiTABCDE*	*caiT*	<		HNS		>	*fixA*	*fixABCX*	0.91
2.43	*coaED*	*coaE*	<				>	*guaC*	*guaC*	2.45
2.99	*gcd*	*gcd*	<				>	*hpt*	*hpt*	3.05
3.06	*can*	*can*	<<				>>	*yadG*	*yadGH*	3.08
5.19	*fadE*	*fadE*	<				>	*lpcA*	*lpcA*	5.25
5.31	*dinJ-yafQ*	*dinJ*	<				>	*yafL*	*yafL*	5.32
5.57	*phoE*	*phoE*	<		HNS		>	*proB*	*proBA*	5.60
6.04	*yagAB-ykgN*	*yagA*	<				>	*yagE*	*yagEF*	6.07
6.68	*matA*	*matA*	<		HNS		>	*ykgL*	*ykgL*	6.71
7.07	*betIBA*	*betI*	<		HNS		>	*betT*	*betT*	7.08
8.26	*yaiS*	*yaiS*	<		HNS		>	*tauA*	*tauABCD*	8.29
12.14	*fimZ*	*fimZ*	<<				>>	*argU*	*argU*	12.15
13.05	*ybdK*	*ybdK*	<<				>	*hokE*	*hokE*	13.08
13.14	*fepA*	*fepA*	<		HNS		>	*fes*	*fes-ybdZ-entFE*	13.19
13.37	*fepDGC*	*fepD*	<<		HNS		>>	*entS*	*entS*	13.40
13.42	*fepB*	*fepB*	<				>	*entC*	*entCEBAH*	13.45
13.73	*dsbG*	*dsbG*	<				>	*ahpC*	*ahpCF*	13.75
15.13	*nagB*	*nagB*	<				>	*nagE*	*nagE*	15.16
16.22	*gltA*	*gltA*	<		HNS		>	*sdhC*	*sdhCDAB*	16.26
16.46	*mngR*	*mngR*	<<		HNS		>>	*mngA*	*mngAB*	16.49
17.51	*ybhU*	*ybhU*	<<				>>	*uvrB*	*uvrB*	17.52
18.29	*rhtA*	*rhtA*	<				>	*ompX*	*ompX*	18.31
18.37	*rybA*	*rybA*	<<				>>	*mntR*	*mntR-ybiR*	18.37
19.13	*ybjL*	*ybjL*	<		HNS		>	*ybjM*	*ybjM*	19.17
21.98	*sulA*	*sulA*	<		HNS		>	*sxy*	*sxy*	21.99
23.75	*csgDEFG*	*csgD*	<<		HNS		>	*csgB*	*csgBAC*	23.78
23.86	*mboC*	*mdoC*	<		HNS		>	*mdoG*	*mboGH*	23.89
24.03	*lpxL*	*lpxL*	<				>	*yceA*	*yceA*	24.05
25.79	*ymfED*	*ymfE*	<		HNS		>	*lit*	*lit*	25.82
26.43	*pliG*	*pliG*	<				>	*ycgL*	*ycgLM*	26.44
26.56	*nhaB*	*nhaB*	<				>	*fadR*	*fadR*	26.60
26.62	*ycgB*	*ycgB*	<				>	*dadA*	*dadAX*	26.66
27.10	*pth-ychF*	*pth*	<<				>>	*ychH*	*ychH*	27.11
27.84	*hns*	*hns*	<		HNS		>	*tdk*	*tdk*	27.86
27.90	*adhE*	*adhE*	<				>	*ychE*	*ychE*	27.97
29.88	*tpx*	*tpx*	<<				>>	*ycjG*	*ycjG*	29.89
29.94	*ycjY*	*ycjY*	<				>	*ycjZ*	*ycjZ*	29.96
31.03	*ldhA*	*ldhA*	<<				>>	*ydbH*	*ydbH-ynbE-ydbL*	31.06
31.90	*azoR*	*azoR*	<				>	*hrpA*	*hrpA*	31.92
32.07	*gapC*	*gapC*	<<				>	*cybB*	*cybB*	32.09
32.10	*rydC*	*rydC*	<				>	*ydcA*	*ydcA*	32.11
32.16	*ydcI*	*ydcI*	<		HNS		>	*ydcJ*	*ydcJ*	32.19
32.48	*yncJ*	*yncJ*	<				>	*hicA*	*hicAB*	32.49
33.50	*bdm-sra*	*bdm*	<		HNS		>	*osmC*	*osmC*	33.51
34.73	*sad*	*sad*	<				>	*yneJ*	*yneJ*	34.76
34.84	*marC*	*marC*	<				>	*marR*	*marRAB*	34.85
35.11	*ydfJ*	*ydfJ*	<				>	*ydfK*	*ydfK*	35.15
37.00	*anmK*	*anmK*	<				>	*slyB*	*slyB*	37.03
37.33	*grxD*	*grxD*	<		HNS		>	*ydhO*	*ydhO*	37.34
37.44	*ydhB*	*ydhB*	<				>	*ydhC*	*ydhC*	37.46
37.78	*ydhZ*	*ydhZ*	<		HNS		>	*pykF*	*pykF*	37.80
38.77	*thrS-infC-rpmI-rplT-pheMS-infA*	*thrS*	<		HNS		>	*arpB*	*arpB*	38.82

41.11	*kdgR*	*kdgR*	<				>	*yebQ*	*yebQ*	41.13
41.39	*pphA*	*pphA*	<				>	*ryeA*	*ryeA*	41.41
41.90	*yobI*	*yobI*	<<				>>	*yebB*	*yebB*	41.91
41.96	*aspA*	*aspS*	<				>	*yecD*	*yecDEN*	42.00
43.23	*yedD*	*yedD*	<				>	*yedE*	*yedEF*	43.24
43.61	*dsrA*	*dsrA*	<<				>>	*yedP*	*yedP*	43.61
43.89	*yedWV*	*yedW*	<<				>>	*yedX*	*yedXY*	43.90
44.49	*insH-6*	*insH-6*	<<				>>	*yoeA*	*yoeA*	44.54
44.99	*yefM-yoeB*	*yefM*	<				>	*hisL*	*hisLGDCBHAFI*	45.00
48.01	*dusC*	*dusC*	<				>	*yohJ*	*yohJK*	48.03
48.30	*folEyeiB*	*folE*	<<				>>	*yeiG*	*yeiG*	48.32
48.42	*yeiE*	*yeiE*	<<				>>	*yeiH*	*yeiH*	48.45
49.59	*napFDAGHBC-ccmBCDEFGH*	*napF*	<<				>>	*yojO*	*yojO*	49.61
49.78	*ompC*	*ompC*	<				>	*micF*	*micF*	49.81
50.90	*yfaZ*	*yfaZ*	<		HNS		>	*nudI*	*nudI*	50.92
51.81	*lrhA*	*lrhA*	<<				>>	*alaA*	*alaA*	51.85
51.96	*yfbV*	*yfbV*	<				>	*ackA*	*ackA-pta*	51.98
53.46	*emrKV*	*emrK*	<				>	*evgA*	*evgAS*	53.49
53.73	*yfdV*	*yfdY*	<				>	*lpxP*	*lpxP*	53.75
54.02	*glk*	*glk*	<				>	*yfeO*	*yfeO*	54.07
54.09	*mntH*	*mntH*	<				>	*nupC*	*nupC*	54.12
54.40	*yfeR*	*yfeR*	<				>	*yfeH*	*yfeH*	54.42
54.96	*ypeA-yfeZ*	*ypeA*	<				>	*amiA*	*amiA-hemF*	54.97
55.64	*aegA*	*aegA*	<<				>>	*narQ*	*narQ*	55.69
55.80	*ypfM*	*ypfM*	<<				>>	*yffB*	*yffB-dapE-ypfN*	55.81
55.97	*dapA-bamC*	*dapA*	<<				>>	*gcvR*	*gcvR*	55.99
56.43	*upp-uraA*	*upp*	<<				>>	*purM*	*purMN*	56.47
56.70	*guaBA*	*guaB*	<<				>>	*xseA*	*xseA*	56.73
57.34	*trmJ*	*trmJ*	<<				>>	*suhB*	*suhB*	57.36
57.81	*glyA*	*glyA*	<<				>>	*hmp*	*hmp*	57.85
58.35	*rpoE-rseABC*	*rpoE*	<<				>>	*nadB*	*nadB*	58.38
59.32	*ratAB*	*ratA*	<				>	*smpB*	*smpB*	59.33
60.00	*ileY*	*ileY*	<<		HNS		>	*ygaQ_1*	*ygaQ_12*	60.01
60.24	*yqaE*	*yqaE*	<<		HNS		>>	*ygaV*	*ygaVF*	60.25
60.83	*mltB*	*mltB*	<		HNS		>	*srlA*	*srlAEBD-gutM*	60.86
61.61	*ygbI*	*ygbI*	<<				>>	*ygbJ*	*ygbJK*	61.63
62.25	*cysJIH*	*cysJ*	<<				>>	*queD*	*queD-ygcN*	62.29
62.56	*queE*	*queE*	<<		HNS		>>	*yqcG*	*ygcG*	62.58
64.14	*lysA*	*lysA*	<<				>>	*lysR*	*lysR*	64.16
65.80	*ygfB-pepF-ubiH-visC*	*ygfB*	<				>	*zapA*	*zapA*	65.82
67.34	*glcDEFGBA*	*glcD*	<				>	*glcC*	*glcC*	67.38
68.75	*glgS*	*glgS*	<		HNS		>	*yqiJ*	*yqiJK*	68.76
69.27	*yqjH*	*yqjH*	<				>	*yqjI*	*yqjI*	69.29
69.31	*aer*	*aer*	<<		HNS		>>	*ygjG*	*ygjG*	69.35
71.93	*mlaFEDCB*	*mlaF*	<<				>>	*yrbG*	*yrbG-kcsDC-lptCAB*	71.95
73.51	*envR*	*envR*	<		HNS		>	*acrE*	*acrEF*	73.54
73.94	*smf*	*smf*	<<				>>	*def*	*def-fmt*	73.96
76.72	*glpEGR*	*glpE*	<				>	*glpD*	*glpD*	76.73
78.33	*yhiN*	*yhiN*	<<		HNS		>>	*pitA*	*pitA*	78.36
78.58	*dinQ*	*dinQ*	<				>	*arsR*	*arsRBC*	78.60
78.67	*insH-11*	*insH-11*	<		HNS		>	*slp*	*slp-dctR*	78.71
80.49	*bax*	*bax*	<				>	*malS*	*malS*	80.51
81.24	*yibIH*	*yibI*	<				>	*mtlA*	*mltADR*	81.26
82.01	*waaQGP-rfaS-waaBIJY-rfaZ-waaK*	*waaQ*	<				>	*waaA*	*waaA-coaD*	82.04
82.28	*ligB*	*ligB*	<				>	*gmk*	*gmk*	82.32
82.60	*yicJI*	*yicJ*	<		HNS		>	*selC*	*selC*	82.64
83.00	*istR-2*	*istR-2*	<<				>	*tisA*	*tisA*	83.01

84.59	*asnC-mioC-mnmG*	*asnC*	<				>	*asnA*	*asnA*	84.60
85.06	*yifB*	*yifB*	<		HNS		>	*ilvL*	*ilvLXGM*	85.10
85.83	*aslA*	*aslA*	<				>	*glmZ*	*glmZ*	85.88
85.95	*hemCDKY*	*hemC*	<				>	*cyaA*	*cyaA*	85.98
87.95	*fdoGHI-fdhE*	*fdoG*	<				>	*fdhD*	*fdhD*	88.02
88.43	*cpxRA*	*cpxR*	<				>	*cpxP*	*cpxP*	88.45
88.70	*glpFKX*	*glpF*	<				>	*zapB*	*zapB*	88.72
88.86	*priA*	*priA*	<				>	*rpmE*	*rpmE*	88.91
88.93	*metJ*	*metJ*	<				>	*metB*	*metBL*	99.94
91.12	*pepE*	*pepE*	<				>	*rluF*	*rluF*	91.14
91.17	*lysC*	*lysC*	<				>	*pgi*	*pgi*	91.21
91.84	*qor*	*qor*	<				>	*dnaB*	*dnaB*	91.87
92.01	*uvrA*	*uvrA*	<				>	*ssb*	*ssb*	92.08
92.09	*yjcB*	*yjcB*	<		HNS		>	*yjcC*	*yjcC*	92.11
94.13	*yjeH*	*yjeH*	<				>	*groS*	*groSL*	94.16
95.19	*ulaG*	*ulaG*	<<		HNS		>>	*ulaA*	*ulaABCDEF*	95.22
95.32	*yjfY*	*yjfY*	<				>	*rpsF*	*rpsF-priB-rpsR-rplI*	95.33
95.51	*ytfG*	*ytfG*	<				>	*ytfH*	*ytfH*	95.53
96.37	*bdcA*	*bdcA*	<<				>>	*bdcR*	*bdcR*	96.39
96.46	*argI*	*argI*	<				>	*rraB*	*rraB*	96.48
96.50	*yjgM*	*yjgM*	<<		HNS		>>	*yjgN*	*yjgN*	96.51
96.99	*insG*	*insG*	<<				>	*yjhB*	*yjhBC*	97.03
97.78	*nanCM*	*nanC*	<<		HNS		>>	*fimB*	*fimB*	97.83
98.46	*yjiR*	*yjiR*	<				>	*yjiS*	*yjiS*	98.49
99.15	*yjjP*	*yjjP*	<				>	*yjjQ*	*yjjQ-bglJ*	99.18
99.21	*fhuF*	*fhuF*	<<				>>	*yjjZ*	*yjjZ*	99.23
99.84	*rob*	*rob*	<		HNS		>	*creA*	*creABC*	99.87
99.96	*arcA*	*arcA*	<				>	*yjjY*	*yjjY*	99.97
	243 genes	137		111	37x2	117		137	226 genes	
		21 TFs		81%		85%		15 TFs		

Supplemental Table S2B

Constitutive Promoters within Type-B Spacers

Map	Operon	Left Gene		P	HNS	P	P	Right Gene	Operon	Map
0.43	insB-1	insB-1	<<				<	insAB-1		0.43
2.76		aceF	>				>	lpd	lpd	0.43
3.55		hrpB	>				>	mrcB	mrcB	3.55
3.61		mrcB	>				>	fhuA	fhuACDB	3.61
3.81		clcA	>				>	erpA	erpA	3.81
3.96	yaeH	yaeH	<				<	yaeI		3.96
5.76	ykfA	ykfA	<				<	perR		5.76
6.22	argF	argF	<				<	insAB-3		6.22
6.28	yagK	yagK	<		HNS		<	yagL		6.28
7.15		betT	>		HNS		>	yahA	yahA	7.15
8.17	frmRAB	frmR	<				<	yaiO		8.17
9.63	(cyoABCDE)	cyoD	<<				<	cyoC		9.63
9.79		bolA	>				>	tig	tig	9.79
9.83		tig	>				>	clpP	clpPX	9.83
10.08		cof	>				>	ybaO	ybaO	10.08
10.27	ylaB	ylaB	<				<	ylaC		10.27
10.30	ylaC	ylaC	<				<	maa		10.30
10.32	maa	maa	<<				<	hha		10.32
11.26		ybbP	>		HNS		>>	rhsD	rhsDC-yibH	11.26
12.29		ybcK	>		HNS		>	ybcL	ybcLM	12.29
12.56	ompT	ompT	<				<	envY		12.59
12.72	nfrBA	nfrB	<				<	cusS		12.72
13.02	nfsB	nfsB	<				<	ybdF		13.02
13.56		entH	>				>	cstA	cstA	13.56
14.15		pagP	>				>	cspE	cspE	14.15
14.19	lipA	lipA	<				<	ybeF		14.19
14.22	ybeF	ybeF	<				<	lipB		14.22
15.30	uof-fur	uof	<				<	fldA		15.30
15.31	fldA	fldA	<				<	ybfE		15.31
15.32	ybfE	ybfE	<				<	ybfF		15.32
15.81		ybfB	>		HNS		>	ybfO	ybfOC	15.81
16.20	ybgD	ybgD	<				<	gltA		16.20
16.61		mngB	>				>>	cydA	cydAB	16.61
16.94	gpmA	gpmA	<				<	galM		16.94
17.03	galETKM	galE	<				<	modF		17.03
18.07	fiu	fiu	<				<	ybiX		18.07
19.21		nfsA	>				>	rimK	rimK-ybjN	19.21
19.38	artJ	artJ	<				<	artM		19.38
19.45	artPIQM	artP	<				<	ybjP		19.45
19.68	ybjE	ybjE	<				<	aqpZ		19.68
20.23		rarA	>				>	serS	serS	20.23
20.68		aroA	>				>	ycaL	ycaL	20.68
21.95	ompA	ompA	<				<	sulA		21.95
22.49	(gfcE-etp-etk)	etp	<<				<	gfcE		22.49
23.37		efeB	>				>	phoH	phoH	23.37
24.14	bssS	bssS	<				<	dinI		24.14
24.44		flgF	>				>	flgG	(flgBCDEFGHIJ)	24.44
24.80		fabG	>				>	acpP	acpP-fabF	24.80

24.84		*fabF*	>				>>	*pabC*	*pabCG-tmk-holB-ycfH*	24.84
25.12		*ycfP*	>				>	*ndh*	*ndh*	25.12
25.21	*mfd*	*mfd*	<				<	*ycfT*		25.21
25.78		*icd*	>				>	*C0293*	*C0293*	25.78
26.07		*pinE*	>				>	*mcrA*	*mcrA*	26.07
26.13	*bluR*	*bluR*	<<				<	*ycgF*		26.13
26.22		*ymgC*	>				>	*ycgG*	*ycgG*	26.22
26.27		*ymgF*	>				>>	*ycgH*	*ycgH*	26.27
26.51		*C0299*	>				>	*umuD*	*umuDC*	26.51
27.07	*(pth-ychF)*	*ychF*	<				<	*pth*		27.07
27.72		*narI*	>				>	*ychS*	*ychS*	27.72
28.00		*ychE*	>				>>	*oppA*	*oppABCDF*	28.00
28.44	*(trpLEDCBA)*	*trpE*	<<				<	*trpL*		28.44
28.51		*yciO*	>				>	*yciQ*	*yciQ*	28.51
28.71		*topA*	>		HNS		>	*cysB*	*cysB*	28.71
28.84		*pgpB*	>				>	*yciS*	*yciSM*	28.84
28.12	*osmB*	*osmB*	<		HNS		<	*yciT*		28.12
29.06	*fabI*	*fabI*	<				<	*ycjD*		29.06
29.08	*ycjD*	*ycjD*	<				<	*sapF*		29.08
29.17	*sapABCDF*	*sapA*	<				<	*ymjA*		29.17
30.86	*ynaE*	*ynaE*	<		HNS		<	*uspF*		30.86
31.32		*paaB*	>				>	*paaC*	*paaCDEFGHIJK*	31.32
32.34		*tehB*	>				>	*ydcL*	*ydcL*	32.34
32.89		*yncH*	>				>>	*rhsE*	*rhsE*	32.89
32.95		*yncI*	>				>	*yncM*	*yncM*	32.95
34.10	*ydeP*	*ydeP*	<		HNS		<	*ydeQ*		34.10
34.27	*hipBA*	*hipB*	<				<	*ydeU*		34.27
34.28	*ydeK*	*ydeK*	<		HNS		<	*lsrK*		34.28
35.31	*essQ-ydfR-rrrQ-ydfP*	*essQ*	<<		HNS		<	*cspB*		35.31
35.51		*dicF*	>				>	*dicB*	*dicB-ydfDE-insD-intQ*	35.51
35.58	*(rspAB)*	*rspB*	<				<	*rspA*		35.58
35.60	*rspAB*	*rspA*	<				<	*ynfA*		35.60
35.98		*ynfM*	>		HNS		>	*asr*	*asr*	35.98
36.26		*rstB*	>				>	*tus*	*tus*	36.26
37.36		*ydhO*	>				>>	*sodB*	*sodB*	37.36
37.49		*ydhC*	>				>	*cfa*	*cfa*	37.49
37.98	*sufABCDSE*	*sufA*	<				<	*rydB*		37.98
38.00	*ydiH*	*ydiH*	<				<	*ydiI*		38.00
38.12		*rprA*	>				>	*ydiL*	*ydiL*	38.12
38.53		*aroH*	>		HNS		>	*ydiE*	*ydiE*	38.53
38.97		*yniC*	>				>	*ydjM*	*ydjM*	38.97
38.99		*ydjM*	>				>	*ydjN*	*ydjN*	38.99
39.02	*ydjO*	*ydjO*	<		HNS		<	*cedA*		39.02
39.26		*nadE*	>				>	*cho*	*cho*	39.26
39.28	*ves*	*ves*	<		HNS		<	*spy*		39.28
39.92	*ydjF*	*ydjF*	<				<	*ydjG*		39.92
40.31		*yeaI*	>				>	*yeaJ*	*yeaJ*	40.31
40.46	*yeaQ*	*yeaQ*	<<				<	*yoaG*		40.46
40.65	*fadD*	*fadD*	<				<	*yeaY*		40.65
41.07	*yobF-cspC*	*yobF*	<		HNS		<	*yebO*		41.07
41.18	*prc*	*prc*	<				<	*proQ*		41.18
41.46		*holE*	>				>	*yobB*	*yobB-exoX*	41.46
41.53	*yebE*	*yebE*	<				<	*yebF*		41.53

41.62	*edd-eda*	*edd*	<				<	*zwf*		41.62
42.14	*torYZ*	*torY*	<		HNS		<	*cutC*		42.14
42.90	*pgsA*	*pgsA*	<<				<	*uvrC*		42.90
42.98	*sdiA*	*sdiA*	<		HNS		<	*yecC*		42.98
43.07	*(fliAZY)*	*fliZ*	<<				<	*fliA*		43.07
43.96		*yedZ*	>				>	*zinT*	*zinT*	43.96
44.29		*amn*	>		HNS		>	*yeeN*	*yeeN*	44.29
44.47	*cobUST*	*cobU*	<				<	*insH-6*		44.47
44.60		*yeeP*	>				>	*isrC*	*isrC*	44.60
44.77	*yeeX*	*yeeX*	<				<	*yeeA*		44.77
45.48	*rfbBDACX*	*rfbB*	<		HNS		<	*galF*		45.48
46.62		*baeR*	>				>	*yegP*	*yegP*	46.62
46.67	*ogrK*	*ogrK*	<				<	*yegZ*		46.67
47.00	*yegX*	*yegX*	<				<	*thiD*		47.00
47.63	*yehS*	*yehS*	<				<	*yehT*		47.63
47.89	*pbpG*	*pbpG*	<				<	*yohC*		47.89
49.05	*yejG*	*yejG*	<				<	*bcr*		49.05
49.23		*yejM*	>				>	*proL*	*proL*	49.23
49.64	*mqo*	*mqo*	<<		HNS		<	*yojI*		49.64
50.40	*yfaL*	*yfaL*	<		HNS		<	*ypaB*		50.40
51.11	*pmrD*	*pmrD*	<				<	*menE*		51.11
51.78	*nuoABCEFGHIJKLMN*	*nuoA*	<<				<	*lrhA*		51.78
52.25	*hisJQMP*	*hisJ*	<				<	*argT*		52.25
52.87	*yfcV*	*yfcV*	<<		HNS		<	*sixA*		52.87
53.61	*yfdV*	*yfdV*	<		HNS		<	*oxc*		53.61
53.67	*frc*	*frc*	<		HNS		<	*yfdX*		53.67
54.54		*cysZ*	>				>>	*cysK*	*cysK*	54.54
55.21		*yffP*	>				>	*yffQ*	*yffQR*	55.21
55.23		*yffR*	>				>	*yffS*	*yffS*	55.23
56.02		*bcp*	>				>	*hyfA*	*hyfABCDEFGHIJR*	56.02
56.40	*(upp-uraA)*	*uraA*	<				<	*upp*		56.40
57.18	*pepB*	*pepB*	<<				<	*iscX*		57.18
57.96	*glmY*	*glmY*	<				<	*purL*		57.96
58.15	*ryfB*	*ryfB*	<<				<	*shoB*		58.15
58.68	*kgtP*	*kgtP*	<				<	*rrfG*		58.68
59.27		*nadK*	>				>	*recN*	*recN*	59.27
59.35		*smpB*	>				>>	*ssrA*	*ssrA*	59.35
59.36		*ssrA*	>				>	*intA*	*intA*	59.36
59.52	*yfjLK*	*yfjL*	<				<	*yfjM*		59.52
59.61		*yfjO*	>				>	*yfjP*	*yfjPQ*	59.61
59.79		*yfjW*	>				>>	*yfjX*	*yfjXYJZ-ypjF*	59.79
60.46		*proW*	>				>	*proX*	*(proVWX)*	60.46
60.51		*ygaY*	>				>	*ygaZ*	*ygaZH*	60.51
60.92		*srlD*	>				>	*gutM*	*gutM-srlRQ*	60.92
61.31	*(hycABCDEFGHI)*	*hycD*	<				<	*hycC*		61.31
61.59		*mutS*	>		HNS		>	*pphB*	*pphB*	61.59
62.01	*(cusABCDE12)*	*cas1*	<<				<	*casE*		62.01
62.70	*mazEFG*	*chpR*	<				<	*relA*		62.70
63.94	*rppH-ptsP*	*rppH*	<				<	*ygdT*		63.94
64.26	*kduI*	*kduI*	<				<	*yqeF*		64.26
64.43		*ygeF*	>				>	*ygeG*	*ygeG*	64.43
64.49		*ygeI*	>				>	*pbl*	*pbi*	64.49
65.59	*ygfF*	*ygfF*	<				<	*gcvP*		65.59

65.82		*zapA*	>				>>	*ssrS*	*ssrS-fau*	65.82
65.84		*ygfA*	>				>	*sibC*	*sibC*	65.84
66.18	*epd-pgk-fbaA*	*epd*	<				<	*yggC*		66.18
67.06	*pppA*	*pppA*	<<				<	*yghJ*		67.06
67.93		*metC*	>				>	*yghB*	*yghB*	67.93
68.24	*mqsRA*	*mqsR*	<				<	*ygiV*		68.24
68.25	*ygiV*	*ygiV*	<<				<	*ygiW*		68.25
68.40	*(tolC-ygjABC)*	*yqiA*	<				<	*cpdA*		68.40
68.61		*yqiC*	>		HNS		>	*ygiL*	*ygiL*	68.61
69.10		*ttdB*	>				>	*ttdT*	*ttdABT*	69.10
69.42		*ebgR*	>				>	*ebgA*	*ebgAC*	69.42
69.55		*ygjJ*	>				>>	*ygjK*	*(ygjJK)*	69.55
69.79		*alx*	>				>	*sstT*	*sstT*	69.79
69.93		*exuT*	>				>	*exuR*	*exuR*	69.93
69.96		*exuR*	>				>	*yqjA*	*yqjA-mzrA*	69.96
70.39		*tdcR*	>		HNS		>	*yhaB*	*yhaBC*	70.39
70.48	*(garPLRK-rrpB)*	*garR*	<<				<	*garL*		70.48
71.84		*ispB*	>				>	*sfsB*	*sfsB*	71.84
72.74	*sspAB*	*sspA*	<				<	*rpsI*		72.74
72.93		*argR*	>		HNS		>	*yhcN*	*yhcN*	72.93
73.46		*prmA*	>				>	*dusB*	*dusB-fis*	73.46
74.68	*bfd-bfr*	*bfd*	<				<	*chiA*		74.68
74.87	*yheO-tusDCB*	*yheO*	<				<	*fkpA*		74.87
75.03		*yheS*	>		HNS		>>	*yheT*	*(yheST)*	75.03
75.39		*yhfL*	>				>	*frlA*	*frlABCDR*	75.39
76.26		*yhgF*	>		HNS		>	*feoA*	*feoABC*	76.26
77.07	*gntR*	*gntR*	<<				<	*yhhW*		77.07
77.55	*rpoH*	*rpoH*	<				<	*ftsX*		77.55
78.19	*yhiI-rbbA-yhhJ*	*yhiI*	<				<	*yhiJ*		78.19
78.22	*yhiJ*	*yhiJ*	<				<	*yhiK*		78.22
78.65		*arsC*	>		HNS		>	*yhiS*	*yhiS*	78.65
78.76	*(hdeABD)*	*hdeB*	<		HNS		<	*hdeA*		78.76
79.79	*(dppABCDF)*	*dppC*	<<				<	*dppB*		79.79
80.65		*yiaK*	>				>	*yiaL*	*yiaKLMNO-lyxK-sgbHU*	80.65
80.87	*yiaWV*	*yiaW*	<		HNS		<	*aldB*		80.87
81.79		*waaC*	>				>	*rfaL*	*(rfaD-waaFC-rfaL)*	81.79
82.24		*yicC*	>				>	*dinD*	*dinD*	82.24
84.18	*pstB-phoU*	*pstB*	<				<	*pstA*		84.18
85.30	*ppiC*	*ppiC*	<				<	*yifO*		85.30
87.25		*polA*	>				>	*spf*	*spf*	87.25
87.36	*glnLG*	*glnL*	<				<	*glnA*		87.36
87.47		*typA*	>				>	*yihL*	*yihLM*	87.47
88.49		*fieF*	>				>	*pfkA*	*pfkA*	88.49
88.73	*rraA*	*rraA*	<				<	*menA*		88.73
90.08		*rplL*	>				>>	*rpoB*	*(rplKAJL-rpoBC)*	90.08
90.27	*sroH*	*sroH*	<				<	*thiH*		90.27
92.24	*yjcF*	*yjcF*	<		HNS		<	*actP*		92.24
92.52		*nrfG*	>		HNS		>	*gltP*	*gltP*	92.52
92.56	*yjcO*	*yjcO*	<				<	*fdhF*		92.56
93.18	*yjdN*	*yjdN*	<				<	*yjdM*		93.18
93.46	*adiA*	*adiA*	<		HNS		<	*melR*		93.46
93.90	*cadBA*	*cadB*	<				<	*cadC*		93.90
94.05	*dcuA*	*dcuA*	<<				<	*aspA*		94.05

94.22	*yjeJ*	*yjeJ*	<				<	*yjeK*		94.22
94.29		*ecnB*	>				>	*sugE*	*sugE*	94.29
94.89		*yjeT*	>				>	*purA*	*purA*	94.89
95.01		*rlmB*	>		HNS		>	*yjfI*	*(nsrR-rnr-rlmB-yjfIJ)*	95.01
95.35		*rpsR*	>				>>	*rplI*	*(rpsF-priB-rpsR-rplI)*	95.35
95.44		*fklB*	>				>	*cycA*	*cycA*	95.44
95.44	*nrdD*	*nrdD*	<				<	*treC*		96.10
97.36	*yjhU*	*yjhU*	<				<	*yjhF*		97.36
97.49	*yjhIHG*	*yjhI*	<				<	*sgcR*		97.49
97.61	*sgcXBCQAER*	*sgcX*	<<				<	*yjhP*		97.61
97.85		*fimB*	>		HNS		>	*fimE*	*fimE*	97.85
97.88		*fimE*	>		HNS		>	*fimA*	*fimAICDFGH*	97.88
98.12		*uxuB*	>				>	*uxuR*	*uxuR*	98.12
98.20	*iadA*	*iadA*	<				<	*yjiG*		98.20
98.30	*yjiK*	*yjiK*	<				<	*yjiL*		98.30
98.51		*yjiS*	>				>	*yjiT*	*yjiT*	98.51
98.63	*mcrBC*	*mcrB*	<				<	*symE*		98.63
99.52		*deoA*	>				>>	*deoB*	*(deoCABD)*	99.52
99.57		*deoD*	>		HNS		>	*yjjJ*	*yjjJ*	99.57
	123 operons (213 genes	123			227			105	105 operons (190 genes)	
	(Internal promoter 13)				HNS 41			(Internal promoter 10)		
