# Supplementary material for: Correction: The Whole Set of Constitutive Promoters Recognized by RNA Polymerase RpoD Holoenzyme of Escherichia coli
Source: PLoS One. 2014 Jun 16;9(6):e100908. doi: 10.1371/journal.pone.0100908 (PMC4059723; doi:10.1371/journal.pone.0100908)
Supplement: File S2 — Republished corrected article. (PDF) [file pone.0100908.s002.pdf]

PLOS ONE

The Whole Set of Constitutive Promoters Recognized by RNA Polymerase RpoD Holoenzyme of *Escherichia coli*

Tomohiro Shimada[1,2,3], Yukiko Yamazaki[4], Kan Tanaka[3], Akira Ishihama[1,2]*

1 Department of Frontier Biosience, Hosei University, Koganai, Tokyo, Japan, 2 Micro-Nano Technology Research Center, Hosei University, Koganai, Tokyo, Japan, 3 Chemical Resources Laboratory, Tokyo Institute of Technology, Nagatsuda, Yokohama, Japan, 4 Genetics Strains Research Institute, National Institute of Genetics, Mishima, Shizuoka, Japan

Abstract

The promoter selectivity of *Escherichia coli* RNA polymerase is determined by the sigma subunit with promoter recognition activity. The model prokaryote *Escherichia coli* contains seven species of the sigma subunit, each recognizing a specific set of promoters. The major sigma subunit, sigma-70 encoded by *rpoD*, plays a major role in transcription of growth-related genes. Concomitant with the increase in detection of promoters functioning *in vivo* under various stressful conditions, the variation is expanding in the consensus sequence of RpoD promoters. In order to identify the canonical sequence of ''constitutive promoters'' that are recognized by the RNA polymerase holoenzyme containing RpoD sigma in the absence of supporting transcription factors, an *in vitro* mixed transcription assay was carried out using a whole set of variant promoters, each harboring one base replacement, within the model promoter with the conserved -35 and -10 sequences of RpoD promoters. The consensus sequences, TTGACA(-35) and TATAAT(-10), were identified to be ideal for the maximum level of open complex formation and the highest rate of promoter opening, respectively. For identification of the full range of constitutive promoters on the *E. coli* genome, a total of 2,701 RpoD holoenzyme-binding sites were identified by Genomic SELEX screening, and using the reconfirmed consensus promoter sequence, a total of maximum 669 constitutive promoters were identified, implying that the majority of hitherto identified promoters represents the TF-dependent ''inducible promoters''. One unique feature of the constitutive promoters is the high level of promoter sequence conservation, about 85% carrying five-out-of-six agreements with -35 or -10 consensus sequence. The list of constitutive promoters provides the community resource toward estimation of the inducible promoters that operate under various stressful conditions in nature.

Citation: Shimada T, Yamazaki Y, Tanaka K, Ishihama A (2014) The Whole Set of Constitutive Promoters Recognized by RNA Polymerase RpoD Holoenzyme of *Escherichia coli*. PLoS ONE 9(3): e90447. doi:10.1371/journal.pone.0090447

Editor: Dipankar Chatterji, Indian Institute of Science, India

Received November 19, 2013; **Accepted** February 1, 2014; **Published** March 6, 2014

Copyright: © 2014 Shimada et al. This is an open-access article distributed under the terms of the Creative Commons Attribution License, which permits unrestricted use, distribution, and reproduction in any medium, provided the original author and source are credited.

Funding: This study was supported by Grants-in-Aid for Scientific Research from MEXT (Ministry of Education, Culture, Sports, Science and Technology of Japan): (A) (21241047) to AI, (B) (18310133) to AI, (C) (25430173) to AI, and Young Scientists (B) (24710214) to TS. This study was performed under the MEXT-Supported Program for the Strategic Research Foundation at Private Universities 208-2012 (S0801037). The funders had no role in study design, data collection and analysis, decision to publish, or preparation of the manuscript.

Competing Interests: The authors have declared that no competing interests exist.

* E-mail: aishiham@hosei.ac.jp

Introduction

The bacterial RNA polymerase core enzyme with the subunit structure $\alpha^2\beta\beta'\omega$ is fully active in catalysis of RNA polymerization but is unable to initiate transcription from promoters. Transcription initiation from gene promoters requires an additional dissociable sigma subunit, which reversibly associates with the core enzyme to form the holoenzyme, and directs the core enzyme to recognize promoters for transcription initiation. Most bacteria encode multiple species of the sigma factor [1,2]. In *Escherichia coli*, seven species of the sigma subunit exist, each recognizing a specific set of promoters [1,3]. The intracellular levels of seven sigma subunits vary depending on cell growth conditions [3,4]. Sigma replacement is a simple mechanism of switching of the pattern of genome transcription [4,5], and the intracellular concentration of seven sigma factors is therefore a primary determinant of the pattern of genome transcription.

The sigma-70, encoded by the *rpoD* gene, is the primary and major sigma, which is responsible for transcription of most of the genes that are expressed in exponentially growing phase of *E. coli* [1,2,6,7]. The holoenzyme containing RpoD sigma recognizes *in vitro* a pair of hexanucleotide sequence elements, TTGACA (-35) and TATAAT (-10) which are situated at 10 and 35 bp upstream, respectively, of transcription initiation sites [2, 6, 8. 9], and a spacer DNA of approximately 17 bp in length separates these two hexanucleotide sequences. This consensus sequence of RpoD promoters was originally proposed based on *in vitro* transcription assays of some model templates by purified RNA polymerase [10,11]. Later the promoter sequences were determined for a variety of functioning promoters *in vivo*, one by one, by using ordinary molecular genetic approaches [12,13]. More recently high-throughput experimental systems such as ChIP-chip analysis of RNA polymerase-bound DNA sequences [14,15] and RNA-Seq analysis of whole sets of transcripts [16–18] have been employed for searching functioning promoters *in vivo*. In parallel, computational approaches have been employed to identify promoters relying on the consensus sequences predicted based on the known promoters. Up to the present time, more than 2,000 experimentally defined or computational predicted promoters have been identified, which are under the direct control of RpoD sigma (see the promoter databases such as RegulonDB [19] and EcoCyc [20]). As a result, the striking diversity appeared in the canonical

promoter sequence, mainly arising from the detection of weak promoters, which are activated only in the presence of positive transcription factors (TFs).

The constitutive promoters have been defined as those that are active *in vivo* in all circumstances, but it is practically impossible to identify the whole set of constitutive promoters under various environmental conditions. Instead we propose in this study to define the "constitutive promoter" as the promoters that are recognized *in vitro* by RNA polymerase RpoD holoenzyme alone in the absence of additional supporting proteins with regulatory functions. For the identification of the whole set of constitutive promoters on the *E. coli* genome, we performed the Genomic SELEX screening system of genome DNA sequences recognized by the RNA polymerase holoenzyme containing RpoD sigma. The Genomic SELEX system was developed to identify DNA sequences recognized *in vitro* by DNA-binding transcription factors [21] and successfully applied for the identification of regulation targets of many TFs [22]. After SELEX-chip screening, a total of 2,071 sites were identified on the *E. coli* genome, which are recognized by the RpoD holoenzyme alone. The location of constitutive promoters within these RpoD holoenzyme-binding regions was then computationally identified using the consensus sequence, which was experimentally determined in this study using the *in vitro* mixed transcription assay [23–25]. The total number of constitutive promoters on the entire *E. coli* genome was thus estimated to be between minimum 492 and maximum 669. This number of constitutive promoters represents only about one-forth of the hitherto identified promoters on the *E. coli* genome, indicating that the rest of promoters listed in the promoter databases represents the "inducible promoters", which are activated in the presence of supporting TFs. One unique feature of the constitutive promoters is the high-level conservation of consensus sequences, TTGACA(-35) and TATAAT(-10), each being separated by a 17-bp spacer.

In the absence of regulatory proteins with repression activity, the constitutive promoters must be always expressed. Based on the Genomic SELEX screening of the binding sites of nucleoid protein H-NS along the *E. coli* genome, we also propose that the H-NS plays a major role in silencing of the unnecessary expression of constitutive promoters.

Results

Genomic SELEX screening of RpoD holoenzyme-binding sequences on the *E. coli* genome

The constitutive promoters are transcribed *in vitro* by the RNA polymerase RpoD holoenzyme alone in the absence of supporting TFs. In order to identify the whole set of constitutive promoters on the entire genome of *E. coli* K-12 M3110, we first performed a mass-screening *in vitro* of the whole set of sequences that are recognized by the reconstituted RpoD holoenzyme. For this purpose, we prepared sigma-free core enzyme by passing the purified RNA polymerase three times through phosphocellulose column chromatography in the presence of 5% glycerol (note that sigma-core interaction becomes stronger in the presence of increasing glycerol concentration as used for prolonged storage of the holoenzyme [26]). The level of remaining sigma subunits was less than 0.1%, if any, as detected by both protein staining and immuno-staining with antibodies against each of all seven species of *E. coli* sigma subunits (RpoD, RpoN, RpoS, RpoH, RpoF, RpoE and FecI) (data not shown). The stoichiometry between core enzyme subunits was also checked by immuno-staining with antibodies against the core subunits, RpoA, RpoB, RpoB and RpoZ. The RpoD holoenzyme fully saturated with RpoD sigma was reconstituted by mixing this sigma-free core enzyme and 4-fold molar excess of purified RpoD sigma, which alone does not bind to DNA.

For the identification of DNA sequences that are recognized by RpoD holoenzyme, we employed the Genomic SELEX screening system [21], in which a library of *E. coli* genome DNA fragments of 200–300 bp in length was used instead of synthetic oligonucleotides with all possible sequences used in the original SELEX method [27–29]. The multi-copy plasmid library of 200–300 bp-long random DNA fragments was constructed from the *E. coli* K-12 W3110 genome [21]. The library used in this study contained 7-fold molar excess of the entire genome, and thus a single and the same sequence might be included in 7 different overlapping segments on average, thereby increasing the resolution of mapping of SELEX fragments. In each experiment of Genomic SELEX screening, the mixture of genome DNA fragments, which was regenerated by PCR from the genome DNA library, was mixed with 2-fold molar excess of the reconstituted RpoD RNA polymerase holoenzyme, and subjected to Genomic SELEX screening. DNA-holoenzyme complexes formed were recovered using the anti-RpoC antibody, which gave the highest level of RNA polymerase recovery among all the anti-core subunits. RNA polymerase-associated DNA was isolated from the antibody precipitates, amplified by PCR, and subjected to next cycles of SELEX. After two-cycles of SELEX screening, the final products of RpoD holoenzyme-bound DNA fragments were subjected to mapping on the genome using a DNA tilling microarray (Oxford Gene Technology, Oxford, UK) [30–32]. On the DNA tilling array used, the 60 b-long DNA probes are aligned at 105 bp-intervals in the order of *E. coli* genome sequence, and therefore approximately 300 bp-long SELEX fragments should bind to two or more consecutive probes. This criterion was employed to avoid the background noise of non-specific binding of RpoD holoenzyme-bound DNA fragments to the tilling array.

The sequences with binding affinity to the RpoD holoenzme formed a number of peaks along the entire *E. coli* genome. By setting the cut-off level of 2.0% relative to the highest peak located within a spacer upstream of *ssrA* (SsrA smRNA) and downstream of *smpB* (SmpB trans-translation factor), a total of 2,701 RpoD homolenzyme-binding peaks were identified, of which 1,075 (40%) are located within 543 intergenic spacers (average 1.98 sites for each spacer) (Fig. 1A). On the other hand, a total of 1,626 (60%) peaks are located inside of 777 open reading frames (average 2.09 peaks per gene) (Fig. 1A). Since the majority of hitherto identified promoters are located within spacers, detailed search for the constitutive promoters was focused on the total of 1,075 peaks within 543 spacers.

Location of the constitutive promoters within the *E. coli* genome

The spacers containing RpoD holoenzyme-binding sites can be classified into three types (Fig. 1A): 404 peaks are located within 177 type-A spacer between bidirectional transcription units (Table 1 for the whole list; see also Table S1); 583 peaks are located within 315 type-B spacers located upstream of one transcription unit but downstream of another transcription unit (Tables 2 and 3 for the whole list; see also Table S2); and 87 peaks are located within type-C spacers downstream of both transcription units of type-C spacers. Based on the transcription direction of flanking genes, the total number of constitutive promoters was predicted to range between minimum 492 (177 A-type plus 315 B-type) and maximum 669 (177x2 A-type plus 315 B-type).

Type-A spacers should contain at least two promoters for bidirectional transcription. The RpoD holoenzyme-binding sites

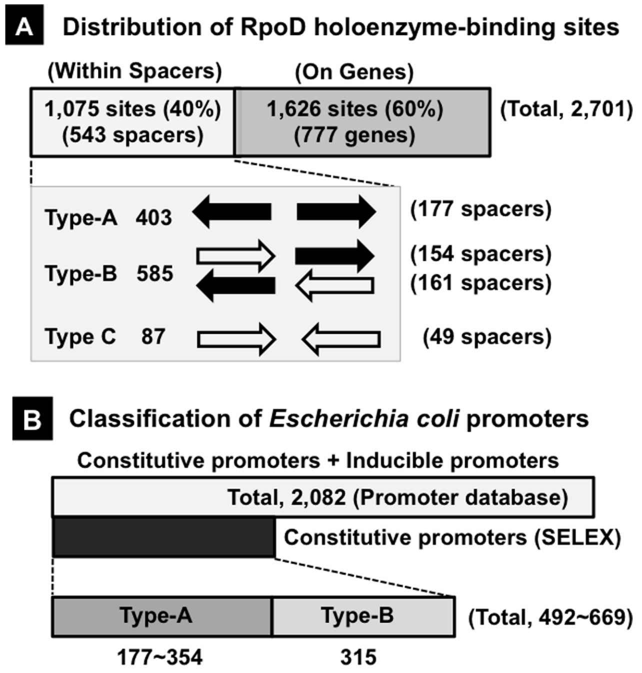

Figure 1. Distribution and classification of the constitutive promoters. Genomic SELEX search of RpoD holoenzyme-binding sequences was performed using the standard procedure [21]. RpoD holoenzyme-bound DNA fragments were recovered by immunoprecipitation using anti-RpoC antibody. SELEX fragments were isolated from the immuno-precipitates and subjected to mapping on the *E. coli* genome by using tilling DNA microarray as described previously [32,33]. **[A] Location of the constitutive promoters.** A total of 2,701 RpoD holoenzyme-binding sites were identified (see Fig. 1), of which 1,075 (40%) are located within intergenic spacers. On the basis of transcription direction of flanking genes, the spacers were classified into three types: type-A between bidirectional transcription units; type-B upstream of one transcription unit but downstream of another transcription unit; and type-C, downstream of both transcription units. **[B] Classification of the constitutive promoters.** A total of 2,082 promoters have been identified and listed in the current versions of RegulonDB and EcoCyc databases, whereas the total number of constitutive promoters identified by Genomix SELEX screening ranges between minimum 492 and maximum 669, indicating that the majority of *E. coli* promoters listed in promoter database are TF-dependent inducible promoters.
doi:10.1371/journal.pone.0090447.g001

identified in a total of 177 type-A spacers should represent promoters for one or both of bidirectional transcription. Close observation of the SELEX-chip pattern indicates that two RpoD holoenzyme-binding sites can be identified if the spacer is longer than 500 bp in length (Fig. 2). For instance, two peaks of RpoD holoenzyme binding were identified within a single and the same Type-A spacer between 755 bp-long *csgD-csgB* and between 1458 bp-long *nanC-fimB* (Fig. 2). Generally this-group promoters associated with stress-response genes are located within long spacers including the binding sites of a number of TFs such as in the spacers of 755 bp-long *csgD-csgB*, 920 bp-long *lrhA-alaA* and 1456 bp-long *nanC-fimB*. One typical example is the promoter for the *csgD* gene encoding the master regulator of biofilm formation, which is under the control of more than 20 TFs [23,33].

The binding of RpoD holoenzyme was identified in a total of 315 type-B spacers (Fig. 1A). The binding of RpoD holoenzyme alone to type-B spacers represents the presence of at least one constitutive promoter for one direction transcription. Even for this group of constitutive promoters, more than two RpoD holoenzyme-binding peaks were identified for some spacers (Fig. 3), indicating the presence of multiple promoters for one and the same transcription units such as the *cydA* promoters within 847 bp spacer and the *yobF* promoters within 670 bp spacer. In good agreement with the presence of multiple peaks for the *mngB-cydA* type-B spacer, five promoters have been identified for the *cydAB* operon encoding cytochrome *bd*-1 terminal oxidase [34–36], of which at least two may be the constitutive promoters that function in the absence of activator TF. The collection of constitutive promoters within type-B spacers also includes a total of 40 internal promoters located within intergenic spacers of single operons (indicted by symbol "Int" in P column of Tables 2 and 3; and Table S2). These internal promoters might play physiological roles under as yet unidentified circumstances. In fact a constitutive internal promoter within the *rplKAJL-rpoBC* operon has been identified [37], which should contribute the expression level control between four ribosomal proteins (L11, L1, L10 and L12) and two RNA polymerase subunits (RpoB and RpoC).

The total number of RpoD promoters (or the transcription initiation sites) listed in the current databases (RegulonDB and EcoCyc) are as many as 2,082, indicating that the majority of known promoters represent TF-dependent "inducible promoters" that are expressed only under the support of positive regulatory proteins (Fig. 1B). Among the total of 2,082 RpoD promoters (or transcription initiation sites) listed in the current version of promoter databases, the promoter sequences are described for a total of 582, of which 434 (75%) are located within the same spacers that contain the constitutive promoters (255 in type-A spacers and 179 in type-B spacers) (shown under black background of P columns in Tables 1, 2 and 3).

Identification of the consensus sequence of constitutive promoters recognized by the RpoD holoenzyme

For identification of the constitutive promoters within type-A and type-B spacers with the binding sites of RpoD holoenzyme, we performed *in silico* search using the consensus sequence of constitutive promoters. The current databases of *E. coli* promoters include both experimentally identified and computationally predicted promoters. The prediction of promoters has been performed using the canonical promoter, TTGACA-17 bp-TATAAT, which was originally identified using the *in vitro* transcription studies [10,11,38]. We then tried to identify the consensus sequence of constitutive promoters recognized *in vitro* by RpoD holoenzyme alone.

In order to experimentally confirm the consensus sequence recognized by RNA polymerase RpoD holoenzyme, we first constructed an ideal promoter with the complete set of consensus TTGACA (-35) and TATAAT (-10) sequences, separated by a spacer of 17 bp in length, starting from the *lacUV5* promoter [23]. To identify the best RpoD promoter giving the highest activity and to examine the role of individual bases within two hexanucleotide sequences, we then constructed a total of 48 variant consensus promoters, each carrying one base replacement at each position of both -35 and -10 signals. For accurate measurement of the RNA product directed by each variant promoter, we employed the *in vitro* mixed transcription system [24,25], in which transcription of each variant promoter was carried out in the simultaneous presence of the ideal promoter with the complete consensus sequence added as an internal reference. The test promoter directed the synthesis of 42 b-long run-off transcript while the ideal promoter directed the synthesis of 22 b-long run-off transcript [23]. The same amounts of two promoter fragments were mixed and incubated with 10-fold molar excess of RpoD holoenzyme for various time periods to allow the formation of open complexes,

Table 1. Constitutive Promoters (Type-A Spacers).

Map	Promoter sequence	*Right Operon*	*Left Gene*	D	RpoD	D	*Right Gene*	*Left Operon*	Promoter sequence
0.87	TTAACG-16-**AATAAT**	*caiTABCDE*	*caiT*	<	**HNS**	>	*fixA*	*fixABCX*	**GTGACA**-17-TAAAGT
1.82	TTGATT-18-TGAAAT	*leuLABCD*	*leuL*	<		>	*leuO**	*leuO*	TTCGCA-17-**TATTAT**
2.43	TTGTCT-18-CATAAA	*coaED*	*coaE*	<		>	*guaC*	*guaC*	TTTATA-18-GATTAT
2.99	ATGACG-18-***TATAAT***	*gcd*	*gcd*	<	**HNS**	>	*hpt*	*hpt*	TTAATA-18-TATAGG
3.06	TTTAAA-17-**TATATT**	*can*	*can*	<		>	*yadG*	*yadGH*	
5.16	ATAACA-17-GATATT	*yafV*	*yafV*	<		>	*ivy*	*ivy*	TTGGAA-17-TATCGT
5.19	TTGCTA-17-TACAAC	*fadE*	*fadE*	<		>	*lpcA*	*lpcA*	CTGACC-17-TGTAGT
5.31	TTGTCG-17-**TACAAT**	*dinJ-yafQ*	*dinJ*	<	**HNS**	>	*yafL*	*yafL*	**TTTACA**-17-TATGTT
5.57	TTAAGA-17-TATATC	*phoE*	*phoE*	<	**HNS**	>	*proB*	*proBA*	TTGTTA-17-**AATAAT**
5.99	CTGCCA-16-**TATGAT**	*insAB-2*	*insA2*	<		>	*insI-1*	*insI-1*	
6.04	TCTACA-17-TTTATT	*yagA--ykgN*	*yagA**	<		>	*yagE*	*yagEF*	TTTACC-16-CATAGT
6.22	TTGTGA-16-**AATAAT**	*argF*	*argF*	<		>	*ykgS*	*ykgS*	TTGAAT-18-**TGTAAT**
6.25	CTGCCA-16-**TATGA**T	*insAB-3*	*insA3*	<		>	*yagJ*	*yagJ*	
6.36	TTTATA-16-TATAGC	*intF*	*intF*	<		>	*ptwF*	*ptwF*	TTGCTA-18-**TATAAA**
7.07	TTGAAC-17-**TTTAAT**	*betIBA*	*betI**	<	**HNS**	>	*betT*	*betT*	TGGACG-17-CTTAAT
7.43		*yahN*	*yahN*	<		>	*yahO*	*yahO*	
8.26	***TTGACA***-18-**TACAAT**	*yaiS*	*yaiS*	<	**HNS**	>	*tauA*	*tauABCD*	**TTGAGA**-16-**TACAAT**
11.08		*qmcA-ybbJ*	*qmcA*	<		>	*ybbL*	*ybbLM*	
12.53	TTGATT-17-**TATAAC**	*ybcY*	*ybcY*	<	**HNS**	>	*tfaX*	*tfaX*	ATGGCA-15-TTAAAT
13.05	TTGCCT-17-TACCAT	*ybdK*	*ybdK*	<		>	*sokE*	*sokE*	ATGGCA-16-AAGAAT
13.14	TTGATT-18-TATTAT	*fepA*	*fepA*	<	**HNS**	>	*fes*	*fes- -entFE*	AAGACA-17-AATAAT
13.37	TCGATA-17-**TATCAT**	*fepDGC*	*fepD*	<		>	*entS*	*entS*	ATGAAA-17-TCTTAT
13.42	ATGATA-17-**TATCAT**	*fepB*	*fepB*	<		>	*entC*	*entCEBAH*	ATGATA-17-**TATCAT**
13.73	**ATGACA**-16-TTTACT	*dsbG*	*dsbG*	<		>	*ahpC*	*ahpCF*	TTGCCC-17-**TGTAAT**
14.09	ATGACA-17-AATTAT	*dcuC*	*dcuC*	<	**HNS**	>	*pagP*	*pagP*	TTAAGA-17-TAAAAA
14.54	CTGACG-17-AATAAG	*ybeQ*	*ybeQ*	<	**HNS**	>	*ybeR*	*ybeR-djiB*	
15.13	TTTACT-17-TATTTT	*nagB*	*nagB*	<		>	*nagE*	*nagE*	**GTGACA**-19-**TTTAAT**
15.69	ATGAAA-16-**TGTAAT**	*kdpFABC*	*kdpF*	<	**HNS**	>	*ybfA*	*ybfA*	TTCGCA-17-TGTAAA
16.22	***TTGACA***-18-TACAAA	*gltA*	*gltA*	<	**HNS**	>	*sdhC*	*sdhCDAB*	TTGTAA-17-**TATACT**
16.46	**TTGAAA**-18-**TATTAT**	*mngR*	*mngR**	<	**HNS**	>	*mngA*	*mngAB*	TTAATA-19-GATAAA
17.93	CTGAAA-18-TATTGT	*ybiA*	*ybiA*	<		>	*dinG*	*dinG*	ATGCCA-16-TACAGT
18.29	TTCAAA-18-TATATC	*rhtA*	*rhtA*	<	**HNS**	>	*ompX*	*ompX*	**TTGACT**-18-TGGAAT
18.37	TTAACG-16-***TATAAT***	*mntS*	*mntS*	<		>	*mntR**	*mntR-ybiR*	ATTACA-18-**TATATT**
19.13	**TTGATA**-19-**TAAAAT**	*ybjL*	*ybjL*	<	**HNS**	>	*ybjM*	*ybjM*	**TTGAAA**-15-**GATAAT**
21.47	**ATGACA**-17-**AATAAT**	*ssuEADCB*	*ssuE*	<		>	*elfA*	*elfAD*	TTTAAA-17-**TATTAT**
21.98	TTAACT-16-TATTCT	*sulA*	*sulA*	<	**HNS**	>	*sxy*	*sxy*	TTGCCC-17-TATTTT
22.59	TGGAGA-18-TACACT	*gfcA*	*gfcA*	<		>	*insA4*	*insAB-4*	CTGCCA-16-**TATGAT**
23.75	**TTATCA**-17-**TAAAAT**	*csgDEFG*	*csgD**	<	**HNS**	>	*csgB*	*csgBAC*	**CTGACA**-17-TGTAGT
23.86	TGGATA-17-CAGAAT	*mdoC*	*mdoC*	<		>	*mdoG*	*mboGH*	GTGAAA-17-CTTAAT
24.03	**TTAACA**-16-TACATT	*waaM*	*waaM*	<		>	*yceA*	*yceA*	TTGCCG-15-**AATAAT**
25.16	TTTATA-17-TAAAAA	*comR*	*comR**	<	**HNS**	>	*bhsA*	*bhsA*	TTCACC-17-**AATAAT**
25.79	TTGTAA-18-***TATAAT***	*ymfED*	*ymfE*	<		>	*lit*	*lit*	**TTGATA**-15-**AATAAT**
26.15	TTTGCA-17-TAAAAC	*bluF*	*bluF**	<	**HNS**	>	*ycgZ*	*ycgZ-ymgC*	**TTGTCA**-18-TTTTAT
26.43	TTTACC-17-TGGAAT	*pliG*	*pliG*	<		>	*ycgL*	*ycgLM*	TTCAGA-18-***TATAAT***
26.48	**ATGACA**-19-**TATCAT**	*hlyE*	*hlyE*	<		>	*umuD*	*umuDC*	CTGGCA-16-**TATAAA**
26.56	CTGACC-19-CATAAA	*nhaB*	*nhaB*	<		>	*fadR**	*fadR*	**TTGATA**-19-GAAAAT

Table 1. Cont.

Map	Promoter sequence	Right Operon	Left Gene	D	RpoD	D	Right Gene	Left Operon	Promoter sequence
26.62	TAGATA-17-TCTAAT	ycgB	ycgB	<		>	dadA	dadAX	TTATCA-17-TATTCT
26.79		ycgR	ycgR	<		>	ymgE	ymgE	TTGCCT-16-TGTAAG
26.92	TTGAAC-16-CGTAAT	dhaKLM	dhaK	<		>	dhaR*	dhaR	TTGCGA-17-CATAAG
27.10	**TTGTCA**-17-TAAACT	pth-ychF	pth	<		>	ychH	ychH	TTGTAA-17-CATAAC
27.37	**TCGACA**-16-**TAAAAT**	chaA	chaA	<		>	chaB	chaBC	**TTGACC**-18-TGTAAA
27.84	ATGAAA-17-**TATCAT**	hns	hns*	<		>	tdk	tdk	TTACCA-17-**TATAAC**
27.90	GTGACG-16-**TCTAAT**	adhE	adhE	<		>	ychE	ychE	TTTAAA-16-**TCTAAT**
29.94	ATGATA-19-**TCTAAT**	ycjY-ymjDC	ycjY	<		>	pgrR*	pgrR	TAGACC-18-**TATCAT**
31.03	TTGAAG-17-CATAGT	ldhA	ldhA	<		>	ydbH	ydbH--ydbL	**TTGAAA**-15-AAAAAT
31.90		azoR	azoR	<		>	hrpA	hrpA	TAGAAA-17-TATATC
32.07	**TTAACA**-19-TTTCAT	gapC	gapC	<	HNS	>	cybB	cybB	**TTGAGA**-19-GAAAAT
32.16	TTAATA-17-**TATCAT**	ydcI	ydcI*	<	HNS	>	ydcJ	ydcJ	**CTGACA**-16-**TATGAT**
32.48	TAAACA-18-**TGTAAT**	yncJ	yncJ	<		>	hicA	hicAB	TAAACA-19-**TATACT**
33.50	**TTGCCA**-15-TATAAA	bdm-sra	bdm	<	HNS	>	osmC	osmC	**TTGATA**-17-**TATATT**
34.73	TTTAAA-17-TATCTT	sad	sad	<		>	yneJ*	yneJ	TTTACT-17-AATAAT
34.84	CTGGCA-17-**GATAAT**	marC	marC	<		>	marR*	marRAB	**TTGACT**-17-**TATTAT**
35.08	ATGCCA-17-TTTAAG	ydfI	ydfI	<		>	ydfK	ydfK	TTCCCA-16-GATAAT
35.43		relBE-hokD	relB	<		>	ydfV	ydfV	
37.33	TTGCCG-17-AATAAT	grxD	grxD	<	HNS	>	ydhO	ydhO	TTAACT-18-**TAGAAT**
37.44	GTGAAA-17-TAATAT	ydhB	ydhB*	<		>	ydhC	ydhC	**TTCACA**-17-TACACT
37.78	TTGTCT-18-TTTAAT	ydhZ	ydhZ	<	HNS	>	pykF	pykF	TTAACT-18-**TATATT**
38.77	TCGTCA-17-**TAAAAT**	thrS- -infA	thrS	<		>	arpB	arpB	ATGATG-16-**TATAAC**
41.11	TTGCGA-17-***TATAAT***	kdgR	kdgR*	<		>	yebQ	yebQ	TTATCA-17-**TATAAA**
41.39	**TTAACA**-15-TGTGAT	pphA	pphA	<		>	ryeA	ryeA	ATCACA-18-TAAAAA
41.57	TTGATT-17-**TATACT**	yebG	yebG	<	HNS	>	purT	purT	AAGACA-18-**TATACT**
41.81	ATGAAA-17-TCTCAT	znuA	znuA	<		>	znuC	znuCB	ATGAGA-17-TTTCAT
41.90	**TTGTCA**-15-TACAAA	yobI	yobI	<		>	yebB	yebB	CTGAAA-17-TATTAC
41.96	TTTCCA-17-TTTCAT	aspS	aspS	<		>	yecD	yecDEN	GACACA-19-**TATTAT**
42.61	**TTGAAA**-17-TATGAC	insAB-5	insA5	<		>	uspC	uspC	**TTGGCA**-16-**TATAAG**
43.11	CTAACA-19-AAAAAT	fliC	fliC	<		>	fliD	fliCST	
43.23	**TTGAAA**-18-**TTTAAT**	yedD	yedD	<		>	yedE	yedEF	TTCAGA-17-**TATTAT**
43.32	**CTGACA**-16-ACTAAT	yedM	yedM	<		>	intG	intG	TTGCCC-16-CACAAT
43.89	TTGTAA-16-**TATAAC**	yedWV	yedW*	<		>	hluH	hluH	TAGAAA-15-CAAAAT
44.99	CTGACA-16-ACTAAT	yefM-yoeB	yefM*	<		>	hisL	hisLG--AFI	TAAACA-19-**TATAAA**
46.28	TTGTTA-18-**TATAAC**	yegI	yegI	<	HNS	>	yegJ	yegJ	ATGATA-17-**TACAAT**
46.68	TAAACA-18-**TATATT**	yegRZ	yegR	<	HNS	>	yegS	yegS	TTTAAA-17-**TATTAT**
48.01	TCAACA-18-CATCAT	dusC	dusC	<		>	yohJ	yohJK	
48.30	ATGAAA-15-**AATAAT**	folE-yeiB	folE	<		>	yeiG	yeiG	CTCACA-17-**TATGAT**
48.42	TTAAAA-19-***TATAAT***	yeiE	yeiE*	<		>	yeiH	yeiH	TTACCA-15-**TATAAA**
49.59		napF- -ccmH	napF	<		>	yojO	yojO	**TTGAGA**-16-**CATAAT**
49.78	ATGAAA-17-**TATCAT**	ompC	ompC	<	HNS	>	micF	micF	TTAAGA-17-AATAAG
50.41	TTGATC-16-**CATAAT**	ygaL	yfaL	<	HNS	>	nrdA	nrdAB	
51.81	CTGCCA-16-**TATGAT**	lrhA	lrhA*	<		>	alaA	alaA	TTAAGA-17-**TATAAC**
51.96	ATGACA-17-CATCAT	yfbV	yfbV	<		>	ackA	ackA-pta	CTGAAA-16-TAGACT
52.11	**GTGACA**-15-**TATAGT**	yfcF	yfcF	<		>	yfcG	yfcG	TCGGCA-15-TATCAA
53.46	TTGATA-17-TTTCAT	emrKV	emrK	<	HNS	>	evgA*	evgAS	***TTGACA***-19-TATATG

Table 1. Cont.

Map	Promoter sequence	*Right Operon*	*Left Gene*	D	RpoD	D	*Right Gene*	*Left Operon*	Promoter sequence
53.73	TTGCCA-17-TACTAT	*yfdV*	*yfdY*	<		>	*lpxP*	*lpxP*	**TCGACA**-18-AACAAT
54.02	TTTACA-17-TATTTT	*glk*	*glk*	<		>	*yfeO*	*yfeO*	CTCACA-17-CATAAC
54.09	TTGATA-17-TAGCAT	*mntH*	*mntH*	<		>	*nupC*	*nupC*	ATGACT-17-**TTTAAT**
54.40		*yfeR*	*yfeR**	<		>	*yfeH*	*yfeH*	TTCTCA-17-TAGAAC
54.49	TTAATA-16-GGTAAT	*zipA*	*zipA*	<		>	*cysZ*	*cysZK*	TTAACT-19-TGAAAT
54.96	TTGGCT-17-**CATAAT**	*ypeA-yfeZ*	*ypeA*	<		>	*amiA*	*amiA-hemF*	**TTGTCA**-17-TAAAAC
55.80	TTCACC-17-**TAGAAT**	*ypfM*	*ypfM*	<	HNS	>	*yffB*	*yffB- -ypfN*	TTCATA-16-**TTTAAT**
55.97		*dapA-bamC*	*dapA*	<		>	*gcvR**	*gcvR*	TTAAAA-18-TCTGAT
56.43	TTGCCG-16-***TATAAT***	*upp-uraA*	*upp*	<	HNS	>	*purM*	*purMN*	ATGATA-17-TATTTT
56.57	**ATGACA**-18-**TATAGT**	*yfgF*	*yfgF*	<	HNS	>	*yfgG*	*yfgG*	**TTGACC**-17-CTTAAT
56.70		*guaBA*	*guaB*	<		>	*xseA*	*xseA*	TCGACT-16-**TAGAAT**
57.34	TCGGCA-18-TAAGAT	*trmJ*	*trmJ*	<		>	*suhB*	*suhB*	AAGACA-17-**TATACT**
57.81	TTGATG-16-**TATAAA**	*glyA*	*glyA*	<		>	*hmp*	*hmp*	**TTTACA**-16-**TATAAG**
58.35	TAAACA-17-CATCAT	*rpoE--rseC*	*rpoE**	<		>	*nadB*	*nadB*	TAGACT-16-**TATAAG**
59.01	**TTGAAA**-16-TATCGT	*aroF-tyrA*	*aroF*	<		>	*yfiL*	*yfiL*	TTTTCA-17-TTTTAT
59.32		*ratAB*	*ratA*	<		>	*smpB*	*smpB*	
60.00	TAGATA-16-**TATCAT**	*ileY*	*ileY*	<	HNS	>	*ygaQ*	*ygaQ_12*	TTATCA-17-**TGTAAT**
60.24	TTAAGA-17-AACAAT	*yqaE*	*yqaE*	<	HNS	>	*ygaV**	*ygaVF*	TTTAGA-17-AATACT
60.27	**TTTACA**-17-**TTTAAT**	*stpA*	*stpA**	<	HNS	>	*alaE*	*alaE*	GTGATA-17-**TCTAAT**
60.83	TTGAAA-16-TTTGAT	*mltB*	*mltB*	<	HNS	>	*srlA*	*srlA- -gutM*	**TTAACA**-18-TATGGT
61.61	TTCACA-18-TATTTT	*ygbI*	*ygbI**	<	HNS	>	*ygbJ*	*ygbJK*	**TTCACA**-16-GTTAAT
62.45	***TTGACA***-16-TGTGAT	*ygcW*	*ygcW*	<	HNS	>	*yqcE*	*yqcE-ygcE*	TTCTCA-18-**GATAAT**
62.56	TTTAAA-17-GTTAAT	*queE*	*queE*	<	HNS	>	*yqcG*	*ygcG*	TTAACA-18-**GATAAT**
63.94	**TTGACG**-18-TTTAGT	*rppH-ptsP*	*rppH*	<		>	*mutH*	*mutH*	TCGGCA-18-**TTTAAT**
65.80		*ygfB- -visC*	*ygfB*	<		>	*zapA*	*zapA*	TTGTCT-17-**TATAGT**
68.75	TAGAGA-19-**TTTAAT**	*glgS*	*glgS*	<	HNS	>	*yqiJ*	*yqiJK*	TTTAAA-15-**TATATT**
69.27	TTAACA-18-TTTTAT	*yqjH*	*yqjH*	<		>	*yqjI**	*yqjI*	TTGCAA-16-**TATAAA**
69.31	TTGATC-18-**TATAGT**	*aer*	*aer*	<	HNS	>	*patA*	*ygjG*	TAAACA-19-**TAAAAT**
69.68	TTTTCA-18-**TATCAT**	*rlmG*	*rlmG*	<		>	*ygjP*	*ygjP*	TTGCCC-18-*TATACC*
70.35	**TTGATA**-16-**TGTAAT**	*trcA- -G*	*tdcA**	<	HNS	>	*tdcR*	*tdcR*	TTTAAA-16-**TATAAA**
71.48	***TTGACA***-18-***TATAAT***	*metY- -pnp*	*metY*	<		>	*argG*	*argG*	ATGAAA-17-AAAAAT
71.93	TGGACT-16-TAAAAC	*mlaFEDCB*	*mlaF*	<		>	*yrbG*	*yrbG- -lptAB*	TTTACT-17-CAAAAT
72.51	TTACCA-16-CATAAA	*insH-10*	*insH10*	<		>	*yhcF*	*yfcF*	CTCACA-18-TTTAAG
72.99	ATAACA-18-**TATATT**	*aaeXAB*	*aaeX*	<	HNS	>	*aaeR**	*aaeR*	**TTGATA**-19-TGTTAT
73.51	TTAAAA-17-**TATATT**	*envR*	*envR**	<	HNS	>	*acrE*	*acrEF*	TTGAGT-19-**AATAAT**
73.94	CTGTCA-18-**TAGAAT**	*smf*	*smf*	<		>	*def*	*def-fmt*	TTGCTA-19-GATAAG
74.39	TTAATA-17-**TATGAT**	*gspAB*	*gspA*	<	HNS	>	*gspC*	*gspD- -LMO*	TTGATT-17-TACTAT
76.72	TTTACG-19-**CATAAT**	*glpEGR*	*glpE*	<		>	*glpD*	*glpD*	**TTGAAA**-19-**TATAAC**
77.61	**GTGCCA**-18-TGTAGT	*ftsYEX*	*ftsY*	<		>	*rsmD*	*rsmD-yhhL*	**TTCACA**-18-TGTTAT
78.27	TTTACA-17-**GATTAT**	*yhiL*	*yhiL*	<	HNS	>	*yhiM*	*yhiM*	CTGAAA-16-**TATAAA**
78.33	TTGCCC-17-**GATAAT**	*yhiN*	*yhiN*	<	HNS	>	*pitA*	*pitA*	TTCACT-18-***TATAAT***
78.58	ATGACG-17-**TATAAA**	*dinQ*	*dinQ*	<		>	*arsR**	*arsRBC*	ATGACG-17-**TATAAA**
78.67	**TTGTCA**-18-***TATAAT***	*insH-11*	*insH11*	<	HNS	>	*slp*	*slp-dctR*	TTTACG-17-**TAAAAT**
78.93	**TTGAAA**-16-**TATAAG**	*gadW*	*gadW**	<	HNS	>	*gadY*	*gadY*	TTCGCA-18-**TATAAA**
80.49	**TTCACA**-17-TCAAAT	*bax*	*bax*	<		>	*malS*	*malS*	**TTGATA**-18-TCAAAT
80.81	TTGTAA-19-TTTTAT	*yiaT*	*yiaT*	<	HNS	>	*yiaU**	*yiaU*	TTCATA-18-**TAAAAT**

Table 1. Cont.

Map	Promoter sequence	Right Operon	Left Gene	D	RpoD	D	Right Gene	Left Operon	Promoter sequence
81.24	TATACA-18-***TATAAT***	yibIH	yibI	<		>	mtlA	mltADR	TCTACA-19-**TACAAT**
81.71	TATACA-18-***TATAAT***	yibB	htrL	<	HNS	>	hldD	rfaD-waaCL	TTAATA-17-CATAAA
82.01	**TCTACA**-17-**TTTATT**	waaQ- -waaK	waaQ	<	HNS	>	waaA	waaA-coaD	CTGACA-16-**TTTTAT**
82.28	GTCACA-16-TAAAAG	ligB	ligB	<		>	gmk	gmk	GTCACA-16-AATAAG
82.60	TTGTAA-19-***TATAAT***	yicJI	yicJ	<	HNS	>	selC	selC	TTATCA-18-**TATAAA**
83.00	**TGGACA**-17-GATACT	istR	istR	<	HNS	>	tisB	tisA	TTGTCC-17-TATACA
84.59	TTTATA-18-**TATGAT**	asnC--mnmG	asnC*	<		>	asnA	asnA	TTGATT-16-**TAAAAT**
85.06	TTGGCC-18-**AATAAT**	yifB	yifB	<	HNS	>	ilvL	ilvLXGM	TTGGCC-18-**AATAAT**
85.39	TTCACC-19-AATAGT	rhlB	rhlB	<		>	trxA	trxA	TTTACG-16-AATAAA
85.83	**TTAACA**-18-**TGTAAT**	aslA	aslA	<	HNS	>	glmZ	glmZ	**TTGAGA**-17-GATGAT
85.95	**CTCACA**-15-**TGTAAT**	hemCDKY	hemC	<		>	cyaA	cyaA	**CTGACA**-18-TAGGAT
87.95	**TCGACA**-15-**TACATT**	fdoG--fdhE	fdoG	<		>	fdhD	fdhD	**TCGACA**-15-TACATT
88.43	TTTGCA-19-TATCGT	cpxRA	cpxR*	<		>	cpxP	cpxP	ATGACG-19-TTTAAA
88.70	**TGGACA**-17-**TACAAT**	glpFKX	glpF	<		>	zapB	zapB	**TGGACA**-17-*TACAAT*
88.86	TTTGCA-18-**TATGAT**	priA	priA	<		>	rpmE	rpmE	CAGACA-17-TATAGC
88.93	TTGAGC-17-**TAAAAT**	metJ	metJ*	<		>	metB	metBL	TTGAGC-17-**TAAAAT**
89.48	TTCATA-17-GATACT	argE	argE	<		>	argC	argCBH	***TTGACA***-18-TATCAA
91.12	TTGGCT-16-TCAAAT	pepE	pepE	<		>	rluF	rluF	ATAACA-17-TATTTT
91.17	TTGACA-16-TTTATT	lysC	lysC	<		>	pgi	pgi	ATCACA-18-**TACAAT**
91.65		plsB	plsB	<		>	dgkA	dgkA	TTAACG-19-**CATAAT**
91.76	**TTGATA**-17-CATAAC	zur	zur*	<		>	yjbL	yjbLM	TTGTCG-18-**AATAAT**
91.84		qorA	qorA	<		>	dnaB	dnaB	TCGTCA-17-TAAAGT
92.01	ATGCCA-15-**TTTAAT**	uvrA	uvrA	<		>	ssb	ssb	TTGACC-18-TGGAAT
92.09	CTAACA-15-TATAGT	yjcB	yjcB	<	HNS	>	yjcC	yjcC	TTTTCA-16-**TATAAA**
94.13	GTGAAA-18-TTTCAT	yjeH	yjeH	<		>	groS	groSL	TTTTCA-17-CAGAAT
95.19	ATCACA-18-**TATCAT**	ulaG	ulaG	<	HNS	>	ulaA	ulaA--EF	TTAACT-15-**GATAAT**
95.32	TTGATT-15-GATCAT	yjfY	yjfY	<		>	rpsF	rpsF- -rplI	TTCAAA-17-**TGTGAT**
95.37	TTGATT-15-GATCAT	yjfZ	yjfZ	<	HNS	>	ytfA*	ytfA	**TTCACA**-16-AATAAA
95.51	**TCGACA**-15-TACATT	qorB	qorB	<		>	ytfH*	ytfH	
96.37	***TTGACA***-17-**TGTAAT**	bdcA	bdcA	<		>	bdcR*	bdcR	TTGATT-17-TACAAA
96.46	TTGCAA-15-**TATAAA**	argI	argI	<		>	rraB	rraB	TTAAAA-16-GATTAT
96.50	**TTGATA**-19-**TAAAAT**	yjgM	yjgM	<	HNS	>	yjgN	yjgN	TTGCCA-18-TATTGT
96.99	**TTAACA**-17-GATAAA	insG	insG	<		>	yjhB	yjhBC	AAGACA-17-TATTGT
97.13	TTAACG-17-**TAGAAT**	insM	insM	<		>	yjhV	yjhV	CTGTCA-16-**TATAAA**
97.33	TTCTCA-17-**GATAAT**	fecIR	fecI**	<		>	insA7	insA-7	**TTAACA**-17-**TATAAG**
97.78	TCAACA-17-**TTTAAT**	nanCM	nanC	<	HNS	>	fimB	fimB	TTGGCA-16-**TATATT**
98.46	**TTCACA**-19-TTTTAT	yjiR	yjiR*	<		>	yjiS	yjiS	TTAACC-15-TAAAAG
99.15	ATGAAA-17-**TTTAAT**	yjjP	yjjP	<	HNS	>	yjjQ*	yjjQ-bglJ	CTGATA-17-**GATAAT**
99.21	**TTGATA**-19-**GATAAT**	fhuF	fhuF	<		>	yjjZ	yjjZ	TTGCAA-16-**TATGAT**
99.96		arcA	arcA*	<		>	yjjY	yjjY	TTGCCA-19-TACAAA
		**300 genes (a)**	**178 (b)**	**K**	**HNS**		**178 (a)**	**291 genes (b)**	
		**1.68 (a/b)**	**26 TFs**		**64**		**19 TFs**	**1.63 (a/b)**	

Table 1. Cont.

Map	Promoter sequence	*Right Operon*	*Left Gene*	D	RpoD	D	*Right Gene*	*Left Operon*	Promoter sequence
		**63 Y-genes**			**36%**			**74 Y-genes**	
		**20 essential**						**19 essential**	

A total of 1,075 RpoD holoenzyme-binding sites were identified within spacers on the entire *E. coli* K-12 W3110 genome. The constitutive promoters were predicted within type-A and type-B intergenic spacers (see Fig. 1A for classficiation). A total of 178 RNA polymerase RpoD holoenzyme-binding sites were identified within type-A spacers, which direct bidirectional transcription. Based on the gene orientation around these promoters, the genes and operons under the control of these promoters were estimated, that are located on either left side (left gene column) or right side (right gene column) of the respective spacers. Genes encoding transcription factors are indicated by star symbols (*) and the operons are shown in the operon columns [note that only the first and the last genes are shown for polycitronic operons]. The directions of transcription for these flanking genes are shown by arrows in column D. The map positions of left-side and right-side genes are shown in the map columns. The essential genes listed in the PEC database are underlined. The promoter sequences were predicted according to the analysis procedure described in Materials and Methods. For some spacers, multiple promoters were identified, of which the best-match promoters with the highest scores are described. The promoter sequence with complete match with the canonical promoter (see Fig. 4) is shown in bold and italic while the promoter sequence with 5-out of-6 match is shown in bold. The spacer including H-NS binding sites are shown as HNS mark in the spacer column. The numbers of hitherto identified promoters are 121 and 133 for left-ward and right-ward transcription, respectively, which correspond to 68 and 75%. Total number of genes under the control of 178 promoters were 300 for left-ward transcription, and 291 for right-ward transcription. The average numbers of genes under one promoter are 1.68 and 1.63 for left-ward and right-ward transcription, respectively. Among the total of 178 RpoD holoenyme-binding sites, 64 (36%) overlap with the H-NS-binding sites.
doi:10.1371/journal.pone.0090447.t001

and then a mixture of substrates and heparin was added to allow the single-round transcription. The final level of transcripts represents the amount of RNA synthesized in 15 min reaction after the addition of substrate mixture into open complexes formed during preincubation for various times up to 30 min (referred to parameter-I in this study). Parameter-I represents the binding affinity of RpoD holoenzyme to the test promoter. On the other hand, the slope of transcript increase represents the rate of open complex formation (referred to parameter-II).

Both parameter-I and -II were determined for each variant promoter for three times and the average values are shown in Fig. 4. In each panel, the promoter activity is compared between four templates with different bases at the same position. Among the collection of -35 variants, the best promoter giving the highest activity of open complex formation (parameter-I) was identified for the consensus TTGACA sequence (Fig. 4A). This indicates that the consensus sequence of promoter -35 influences the binding affinity of RNA polymerase to the promoter in agreement with the previous estimation [23]. As to the promoter -10 signal, the best sequence giving the highest rate of open complex formation (parameter-II) was identified for the consensus TATAAT (Fig. 4B), indicating that promoter -10 influences the rate of promoter opening.

Using this experimentally confirmed consensus sequence, TTGACA-17 bp-TATAAT, of the constitutive promoter recognized by RpoD holoenzyme alone, we search for the location of constitutive promoters within both type-A and type-B spacers including RpoD holoenzyme-binding sites.

Unique features of the RpoD constitutive promoters

After sequence analysis of the entire genome of *E. coli* K-12, we realized that there is no ideal sequence of RpoD promoter with perfect matching to this consensus sequence. We then analyzed whether the constitutive RpoD promoters harbor unique sequences. By setting a rather severe screening condition of the sequence matching of more than 4 out of 6 bases for both -35 and -10 signals (total score, higher than 8; the highest score, 12) and with a spacer length of 17 plus/minus 2 (score 3 for 17 bp spacer, score 2 for 16 and 18 bp spacers, and score 1 for 15 and 19 bp spacers), a total of as many as 316 promoter sequences (89%) were identified among 354 predicted constitutive promoters within type-A spacer, and a total of 226 promoter sequences (82%) were identified among 276 predicted promoters excluding the internal promoters within type-B spacer. Overall the total amount of constitutive promoters with high-level (higher than 4/6) matching with the consensus RpoD promoter for both -36 and -10 signals is more than 85% (Fig. 5A-1). This is in sharp contrast with the collection of 582 experimentally defined promoters, in which the amount of promoters containing a high-level (4/6) agreement to both -35 or -10 signals are less than 40% (Fig. 5B-1). On average, only less than half of the 12 canonical bases of the -35 and -10 boxes are conserved among the experimentally identified promoters [39]. The length of spacers between -35 and -10 signals ranges from minimum 14 to maximum 21 bp. Only 10–20% efficient promoters have been proposed to include either -35 or -10 box that resembles the consensus with five-out-of-six bases (5/6 agreement).

Using a total of 669 sequences of the constitutive promoter with high-level of sequence conservation, the consensus sequence of RpoD promoter was reevaluated. The Logo plot, shown in Fig. 5A-2, indicates that: 1) the patterns of conserved bases of both -35 and -10 sequences are similar for both left and right genes with both type-A and type-B spacers (data not shown); and 2) a significant difference exists in the consensus sequence of both -35 and -10 signals between a total of 543 newly identified constitutive promoters and a total of 582 experimentally identified promoters. The Logo pattern generated from the alignment of a total of 582 experimentally identified *E. coli* promoters, shown in Fig. 5B-2, indicates that the level of sequence conservation is significantly higher for -10 than -35, and the conserved bases are limited, TT (positions 1 and 2) of -35 signal and TA (positions 1 and 2) and T (position 6) of -10 signal. This Logo pattern agrees well with that analyzed by Mitchell *et al.* [40] using the promoter set of RegulonDB [41]. In the case of constitutive promoters, -35 and -10 sequences are equally well-conserved (Fig. 5A-2). Within -35 sequence, TT (position 1 and 2) and AA (positions 4 and 6) are well-conserved but G at position 3 and C at position 5 appear less important even for the constitutive promoters. On the other hand, all six bases of TATAAT within -10 signal are equally well-conserved, indicating their equal contribution to the promoter activity. The marked difference of the consensus sequence of experimentally identified RpoD promoters from that of constitutive promoters indicates that the promoter databases include a number of inducible promoters that were active under the support of positive TFs.

Table 2. Constitutive Promoters (Type-B Spacers) (Leftward transcription).

Map	Promoter sequence	*Left Operon*	*Left Gene*	D	RpoD	D	*Right Gene*	*Right Operon*
0.44	CTGCCA-16-**TATGAT**	*insAB-1*	*insA-1*	<		<	*rpsT*	*rpsT*
0.85		*caiABCDE*	*caiA*	<		<	*caiT*	*caiTABCDE*
3.96		*yaeH*	*yaeH*	<		<	*yaeI*	*yaeI*
5.76	TTGAAA-15-**TATCAT**	*ykfA*	*ykfA*	<		<	*perR*	*perR*
5.99	Internal Promoter	*(insAB-afuBC)*	*afuB*	<		<	*ykgN*	*(yagAB-ykgN)*
6.02	Internal Promoter	*(yagAB-ykgN)*	*ykgN*	<		<	*yagB*	*(yagAB-ykgN)*
6.28	TTTAGA-16-***TATAAT***	*yagK*	*yagK*	<	**HNS**	<	*yagL*	*yagL*
6.34	TTAAAA-17-**TATCAT**	*yagN*	*yagN*	<	**HNS**	<	*intF*	*intF*
6.72	**TTGAAA**-17-TATCTT	*ykgMO*	*ykgM*	<	**HNS**	<	*ykgR*	*ykgR*
6,84		*ykgIB*	*ykgI*	<	**HNS**	<	*ykgC*	*ykgC*
6.98	GCGACA-16-**TATATT**	*ykgH*	*ykgH*	<	**HNS**	<	*betA*	*betA*
7.77	Internal Promoter	*(lacZYA)*	*lacA*	<		<	*lacY*	*(lacZYA)*
8.14	Internal Promoter	*(frmRAB)*	*frmA*	<		<	*frmR*	*frmRAB*
8.17	TTGACA-15-TATAGT	*frmRAB*	*frmR**	<		<	*yaiO*	*yaiO*
9.47	Internal Promoter	*(xseB-ispA-dxs)*	*ispA*	<		<	*xseB*	*(xseB-ispA-dxs)*
9.63	Internal Promoter	*(cyoABCDE)*	*cyoD*	<		<	*cyoC*	*(cyoABCDE)*
10.27	TCCACA-17-**TACACT**	*ylaB*	*ylaB*	<		<	*ylaC*	*ylaC*
10.30		*ylaC*	*ylaC*	<		<	*maa*	*maa*
10.33		*hha*	*hha*	<		<	*tomB*	*tomB-hha*
10.34	ATGAAA-17-**TATAGT**	*tomB-hha*	*tomB*	<	**HNS**	<	*acrB*	*(acrAB)*
14.19	ATGGCA-17-TACATT	*lipA*	*lipA*	<		<	*ybeF*	*ybeF*
14.22	TTTACA-15-**TATATT**	*ybeF*	*ybeF**	<	**HNS**	<	*lipB*	*lipB*
15.30	TTGTAA-18-**TACAAT**	*uof-fur*	*uof*	<		<	*fldA*	*fldA*
15.31	TGGGCA-18-AATAAG	*fldA*	*fldA*	<		<	*ybfE*	*ybfE*
15.32	TTGGCG-18-**TATTAT**	*ybfE*	*ybfE*	<		<	*ybfF*	*ybfF*
16.20	TAAACA-16-**TAAAAT**	*ybgD*	*ybgD*	<	**HNS**	<	*gltA*	*gltA*
16.94	TTCAAA-17-CATATT	*gpmA*	*gpmA*	<		<	*galM*	*(galETKM)*
17.03	ATGAAA-17-TAAAAA	*galETKM*	*galE*	<	**HNS**	<	*modF*	*modEF*
19.38	TTGTCC-17-TAAATT	*artJ*	*artJ*	<		<	*artM*	*artM*
19.45	TTAACT-18-**CATAAT**	*artPIQM*	*artP*	<	**HNS**	<	*ybjP*	*ybjP*
19.68	TTGACG-19-**TGTAAT**	*ybjE*	*ybjE*	<		<	*aqpZ*	*aqpZ*
20.54	**TTTACA**-17-**AATAAT**	*focA-pflB*	*focA*	<		<	*ycaO*	*ycaO*
22.15	**TTGAAA**-16-TATATC	*hspQ*	*hspQ*	<		<	*rlmI*	*rlmI*
24.14	ATGACA-17-TATAAA	*bssS*	*bssS*	<	**HNS**	<	*dinI*	*dinI*
25.21	TTGCTA-16-GATAAT	*mfd*	*mfd*	<		<	*ycfT*	*ycfT*
25.49	Internal Promoter	*(potABCD)*	*potB*	<		<	*potA*	*(potABCD)*
26.11	TTCATA-17-**CATAAT**	*iraM*	*iraM*	<	**HNS**	<	*ycgX*	*ycgX*
26.13	TTTAAA-16-**AATAAT**	*bluR*	*bluR**	<	**HNS**	<	*bluF*	*bluF*
27.07	Internal Promoter	(*ychF*)	*ychF*	<		<	*pth*	*pth*
28.17	**TTAACA**-17-TATGTT	*kch*	*kch*	<		<	*yciI*	*yciI*
28.91	TTAACA-17-**TATTAT**	*osmB*	*osmB*	<	**HNS**	<	*yciT*	*yciT*
28.92	TTGAGG-16-TATTTT	*deoT*	*deoT*	<		<	*yciZ*	*yciZ*
29.06	GTGAAA-17-GAGAAT	*fabI*	*fabI*	<		<	*ycjD*	*ycjD*
29.08	CTGACA-16-CAGAAT	*ycjD*	*ycjD*	<		<	*sapF*	*sapDF*
29.17	ATGACA-15-**TTTAAT**	*sapABCDF*	*sapA*	<		<	*ymjA*	*ymjA*
30.85	TGTACA-16-**AATAAT**	*pinR*	*pinR*	<		<	*ynaE*	*ynaE*

Table 2. Cont.

Map	Promoter sequence	*Left Operon*	*Left Gene*	D	RpoD	D	*Right Gene*	*Right Operon*
30.86	CTGACA-17-TACCAT	*ynaE*	*ynaE**	<	HNS	<	*uspF*	*uspF*
31.54	**CTGACA**-17-AATAAC	*ynbG*	*ynbG*	<		<	*insC-2*	*insCD-2*
33.21	TTCACC-16-**TCTAAT**	*narU*	*narU*	<		<	*yddJ*	*yddJ*
33.25	Internal Promoter	*(yddLKJ)*	*yddK*	<		<	*yddL*	*yddLKJ*
33.42	Internal Promoter	*yddM*	*yddM*	<		<	*adhP*	*adhP*
33.58	Internal Promoter	*(ddpXABCDF)*	*ddpB*	<		<	*ddpA*	*(ddpXABCDF)*
34.03	**TTGTCA**-16-TATTAA	*ydeN*	*ydeN*	<		<	*ydeO*	*ydeO*
34.10	TTGAAG-16-**TATATT**	*ydeP*	*ydeP*	<	HNS	<	*ydeQ*	*ydeQ*
34.27	**TTGACT**-16-TAAAAC	*hipBA*	*hipB*	<		<	*yneO*	*yneO*
34.28	CTGACA-17-**TTTAAT**	*yneO*	*yneO*	<	HNS	<	*lsrK*	*lsrRK*
34.68	TTGCCG-19-TATCTT	*yneF*	*yneF*	<		<	*yneG*	*yneG*
34.71	TTTTCA-17-TAGAAA	*yneHG*	*yneH*	<		<	*sad*	*sad*
35.31	TTTATA-16-**AATAAT**	*essQ- -rrrQ-ydfP*	*essQ*	<	HNS	<	*cspB*	*cspB*
35.58	Internal Promoter	*(rspAB)*	*rspB*	<		<	*rspA*	*rspAB*
35.60	TTGTCA-17-TATACG	*rspAB*	*rspA*	<		<	*ynfA*	*ynfA*
36.28	TTAACG-17-AAAAAT	*fumC*	*fumC*	<		<	*fumA*	*fumA*
36.52	TTAACC-17-TATACG	*uidR*	*uidR**	<		<	*hdhA*	*hdhA*
37.12	TTCAAA-15-TACACT	*sodC*	*sodC*	<		<	*ydhF*	*ydhF*
37.98	ATCACA-16-**GATAAT**	*sufABCDSE*	*sufA*	<		<	*rydB*	*rydB*
38.00	TTGTCA-16-CATATT	*ydiH*	*ydiH*	<		<	*ydiI*	*ydiJI*
38.76		*infC-rpmI-rplT*	*infC*	<		<	*thrS*	*thrS- -rplT-pheST*
39.02	ATGACT-16-**AATAAT**	*ydjO*	*ydjO*	<	HNS	<	*cedA*	*cedA*
39.28		*ves*	*ves*	<		<	*spy*	*spy*
39.92	TTGAAA-17-**GATAAT**	*ydjF*	*ydjF**	<		<	*ydjG*	*ydjG*
40.46	TTGCCC-19-TTTTAT	*yeaQ*	*yeaQ*	<		<	*yoaG*	*yoaG*
40.65	TTAATA-18-**TATCAT**	*fadD*	*fadD*	<		<	*yeaY*	*yeaY*
41.07	TTGCCA-17-GATAAC	*yobF-cspC*	*yobF*	<	HNS	<	*yebO*	*yebO*
41.18		*prc*	*prc*	<		<	*proQ*	*proQ*
41.53	TTGTCC-15-CACAAT	*yebE*	*yebE*	<		<	*yebF*	*yebF*
41.62	TTCACC-17-TACACT	*edd-eda*	*edd*	<		<	*zwf*	*zwf*
41.89	TGGATA-17-**TATCAT**	*ruvAB*	*ruvA*	<	HNS	<	*yobI*	*yobI*
42.14	**GTGACA**-18-TAAAAA	*torYZ*	*torY*	<	HNS	<	*cutC*	*cutC*
42.90		*pgsA*	*pgsA*	<		<	*uvrC*	*uvrC*
42.98	TTGCAA-17-**AATAAT**	*sdiA*	*sdiA**	<	HNS	<	*yecC*	*yecC*
43.07	Internal Promoter	*(fliAZY)*	*fliZ*	<		<	*fliA*	*fliAZY*
44.47	**TTGAGA**-16-**TATATT**	*cobUST*	*cobU*	<		<	*insH-6*	*insH-6*
44.49	TGTACA-17-CATGAT	*yoeG*	*yoeG*	<		<	*yoeH*	*yoeH*
44.55	TTTTCA-18-***TATAAT***	*yoeH*	*yoeH*	<	HNS	<	*insD-3*	*insD-3*
44.77	**TTGTCA**-15-AGTAAT	*yeeX*	*yeeX*	<		<	*yeeA*	*yeeA*
44.95		*yeeY*	*yeeY*	<		<	*yeeZ*	*yeeZ*
45.26	ATTACA-19-**TATCAT**	*insH-7*	*insH-7*	<	HNS	<	*wbbK*	*(wbbIJK)*
45.37		*glf-wbbH*	*glf*	<		<	*rfbX*	*(rfbBDACX)*
45.48	**TTCACA**-18-TGGAAT	*rfbBDACX*	*rfbB*	<		<	*galF*	*galF*
46.67		*ogrK*	*ogrK*	<		<	*yegZ*	*yegZ*
46.92	Internal Promoter	*(gatZABCD)*	*gatB*	<		<	*gatA*	*(gatZABCD)*
47.00		*yegX*	*yegX*	<		<	*thiD*	*thiMD*
47.63		*yehS*	*yehS*	<		<	*yehT*	*yehT*

Table 2. Cont.

Map	Promoter sequence	Left Operon	Left Gene	D	RpoD	D	Right Gene	Right Operon
47.89	**TTGACG**-18-**TATGAT**	pbpG	pbpG	<		<	yohC	yohC
49.05	TTGATG-17-**TATCAT**	yejG	yejG	<		<	bcr	bcr
49.64	TTGATG-16-TGCAAT	mqo	mqo	<	HNS	<	yojI	yojI
51.11	GTGAAA-16-**GATAAT**	pmrD	pmrD	<		<	menE	menE
51.61	Internal Promoter	(nuoABC- -HIJKL)	nuoH	<		<	nuoG	(nuoAB- -GHIJKL)
51.73	Internal Promoter	(nuoABC- -HIJKL)	nuoC	<		<	nuoB	(nuoAB- -GHIJKL)
51.78	***TTGACA***-18-TAAAAA	nuoAB- -IJKLMN	nuoA	<		<	lrhA	lrhA
52.25	**TTGAAA**-16-**TTTAAT**	hisJQMP	hisJ	<		<	argT	argT
52.87	**TTGAAA**-17-**TATAGT**	yfcV	yfcV	<	HNS	<	sixA	sixA
53.61	**CTGACA**-19-CATTAT	yfdV	yfdV	<		<	oxc	oxc
53.67	TTTATA-18-**AATAAT**	frc	frc	<	HNS	<	yfdX	yldX
57.16		sseB	sseB	<		<	pepB	pepB
57.96	TTAACT-17-**TCTAAT**	glmY	glmY	<		<	purL	purL
58.68	ATGATA-15-AATATT	kgtP	kgtP	<		<	rrfG	(rrsG- -rrlG-rrfG)
59.52	TAGACG-18-TGGAAT	yfjLK	yfjL	<		<	yfjM	yfjM
59.97	TTATCA-18-**TTTAAT**	ypjC	ypjC	<	HNS	<	ileY	ileY
61.31	Internal Promoter	(hycABCDEFGHI)	hycD	<		<	hycC	(hycABCDEFGHI)
62.01	Internal Promoter	(cusABC -ygbTF)	ygbT	<		<	casE	(cusABC- -ygbTF)
62.70	**TTGATA**-15-**TATGAT**	mazEFG	chpR	<		<	relA	relA
64.26	**TTGAAA**-17-**TATCAT**	kduI	kduI	<	HNS	<	yqeF	yqeF
64.40		yqeL	yqeL	<		<	yqeK	yqeK
65.59	GTGACG-15-TTCAAT	ygfF	ygfF	<		<	gcvP	gcvP
66.18	TTCCCA-16-TGTGAT	epd-pgk-fbaA	epd	<	HNS	<	yggC	(yggDC)
67.03	GTGACG-15-TTCAAT	yghF	yghF	<		<	yghG	yghG
67.77	TTGCCT-17-GACAAT	yghW	yghW	<		<	yghX	yghX
68.24	TTAACC-15-TAAAGT	mqsRA	mqsR	<		<	ygiV	ygiV
68.40	Internal Promoter	(nudF- -yqiA-parE)	yqiA	<		<	cpdA	(nudF- -yqiA-parE)
68.81	**TTGACG**-17-TAAAGT	sibD	sibD	<		<	sibE	sibE
71.46		rimP-nusA-infB	rimP	<		<	metY	metY
71.60		folP-glmM	folP	<		<	ftsH	ftsH
72.18	TTGAGG-18-CACAAT	arcB	arcB	<		<	yhcC	yhcC
72.74	TGGCCA-18-TAAAAA	sspAB	sspA	<		<	rpsI	rpsI
74.68	**TTGAAA**-17-TATTTT	bfd-bfr	bfd	<		<	chiA	chiA
74.87	TGGAAA-16-ATTAAT	yheO-tusDCB	yheO*	<		<	fkpA	fkpA
75.16	**TTGCCA**-17-CATATT	argD	argD	<		<	pabA	pabA
76.67		glpR	glpR*	<		<	glpG	glpG
77.07	TTAGCA-17-TTTAGT	gntR	gntR*	<		<	yhhW	yhhW
77.55	**TTCACA**-19-GATAAA	rpoH	rpoH	<		<	ftsX	ftsX
78.19	**TAGACA**-16-TACTAT	yhiI-rbbA-yhhJ	yhiI	<		<	yhiJ	yhiJ
78.22	TTGACG-19-***TATAAT***	yhiJ	yhiJ	<	HNS	<	yhiL	yhiL
78.76	TGAACA-17-TAAAAG	(hdeABD)	hdeB	<	HNS	<	hdeA	hdeABD
78.95	TTAATA-16-**TGTAAT**	gadX	gadX*	<	HNS	<	gadA	gadA
78.98	TTAATA-17-**TATATT**	gadAX	gadA	<	HNS	<	yhjA	yhjA
79.79	Internal Promoter	(dppABCDF)	dppC	<		<	dppB	(dppABCDF)
80.87	CAGACA-17-**TATAAA**	yiaWV	yiaW	<	HNS	<	aldB	aldB
83.00	CTGAAA-19-TGTAAA	ivbL- -uhpABC	ivbL	<		<	istR	istR

Table 2. Cont.

Map	Promoter sequence	*Left Operon*	*Left Gene*	D	RpoD	D	*Right Gene*	*Right Operon*
83.53		*gyrB*	*gyrB*	<		<	*recF*	*recF*
84.18		*pstB-phoU*	*pstB*	<		<	*pstA*	*(pstCA)*
85.30	**CTGACA**-18-GATCAT	*ppiC*	*ppiC*	<		<	*yifO*	*yifO*
86.22		*yigF*	*yigF*	<		<	*yigG*	*yigG*
87.36		*glnLG*	*glnL*	<		<	*glnA*	*glnA*
88.73	**TTGCCA**-18-**TATACT**	*rraA*	*rraA*	<		<	*menA*	*menA-rraA*
92.24	TTCCCA-15-TAAACT	*yjcF*	*yjcF*	<	HNS	<	*actP*	*actP*
92.56		*yjcO*	*yjcO*	<		<	*fdhF*	*fdhF*
93.18	TTATCA-16-***TATAAA***	*yjdN*	*yjdN*	<		<	*yjdM*	*yjdM*
93.46	TTTACA-17-GATACT	*adiA*	*adiA*	<	HNS	<	*melR*	*melR*
93.90	TTGAGT-19-***TATAAT***	*cadBA*	*cadB*	<	HNS	<	*cadC*	*cadC*
94.05	ATAACA-17-TAAAAA	*dcuA*	*dcuA*	<		<	*aspA*	*aspA-dcuA*
94.22	TTAACC-17-TAGAGT	*yjeJ*	*yjeJ*	<		<	*epmB*	*epmB*
95.44	TTTTCA-16-AAAAAT	*nrdD*	*nrdD*	<		<	*treC*	*treC*
97.36	TTAAGA-15-**TTTAAT**	*yjhU*	*yjhU**	<		<	*yjhF*	*yjhF*
97.49	TTCAAA-17-**TTTAAT**	*yjhIHG*	*yjhI**	<		<	*sgcR*	*sgcR*
97.61	TTTACC-17-TATCAC	*sgcXBCQAER*	*sgcX*	<		<	*yjhP*	*yjhQP*
98.20		*iadA*	*iadA*	<		<	*yjiG*	*(yjiHG-iadA)*
98.30	CTGACC-19-**TACAAT**	*yjiK*	*yjiK*	<		<	*yjiL*	*yjiL*
98.63	TTTACC-16-AAAAAT	*mcrBC*	*mcrB*	<		<	*symE*	*symE*
99.60		*lplA*	*lplA*	<		<	*ytjB*	*ytjB*
		**290 (a)**	**181 (b)**		**HNS**			
		**1.80 (a/b)**	**15 TFs**		**39**			
		**69 Y-genes**			**24%**			
		**14 essential**						

Among the total of 1,075 RpoD holoenzyme-binding sites, 181 are located within type-B spacers upstream of left-side genes and downstream of right-side genes, indicating that these promoters direct leftward transcription. The genes and operons under the control of these 181 promoters were estimated, of which 16 represent putative internal promoters. Descriptions and symbols are as in Table 1. A total of 15 genes encoding transcription factors are indicated by star symbols (*). The essential genes listed in the PEC database are underlined within the operons. The promoter sequence with complete match with the canonical promoter (see Fig. 4) is shown in bold and italic while the promoter sequence with 5-out of-6 match is shown in bold. The spacers including H-NS binding sites are marked as HNS in the spacer column. Total number of genes under the control of these 181 promoters were 290 (1.80 gene per promoter). Among the total of 181 RpoD holoenyme-binding sites, 39 (24%) overlap with the H-NS-binding sites.
doi:10.1371/journal.pone.0090447.t002

Constitutive promoters carrying the binding site of silencer H-NS

The constitutive promoters direct transcription by the RpoD holoezyme alone in the absence of positive transcription factors. Expression *in vivo* of the constitutive promoters must be repressed under conditions where the target genes are not needed. One possible transcription factor with the silencing activity is H-NS, one of the major core nucleoid proteins with functional dichotomy [42]. H-NS is known as a universal silencer for repression of a number of xenogeneic genes [43,44]. In parallel with the mapping of RpoD promoters, we identified a total of 987 H-NS-binding on the *E. coli* genome using the Genomic SELEX system [45]. Within a total of 492 spacers (177 type-A and 315 type-B) with RpoD holoenzyme-binding sites, H-NS binding sites were identified within 63 type-A (36%) (indicated as H-NS in column P of Table 1) and 77 type-B spacers (24%) (Tables 2 and 3).

The genome-silencing function of H-NS is attributable to its unique mode of DNA binding. H-NS recognizes and binds to intrinsically curved (or bent) DNA with AT-rich sequences, and such curved sequences are often located near promoters and also within transported DNA segments such as phage genomes [46,47]. Promoter-associated DNA curvature provides H-NS with the initial contact site, leading to form DNA-H-NS filaments via cooperative protein-protein interaction [48]. Since once H-NS binds within one spacer, it could be fully covered with H-NS by spreading from the initial binding sites through high level of protein-protein cooperativity. Thus, if H-NS binds within one type-A spacer, it influences the silencing effect to promoters for both of the bidirectional transcription. Taken together we estimated that a total of 203 promoters (63x2 + 77) or 31% among a total of 669 constitutive promoters could be under the control of genome silencer H-NS, indicating that one third of the constitutive promoters are subject to silencing by H-NS.

Among the total of 203 possible targets of H-NS silencing, a total of 20 promoters are known to be under the control of H-NS. Up to the present time, however, the involvement of H-NS in promoter regulation has not analyzed seriously because this bifunctional nucleoid protein is abundant, playing both architectural and regularatory roles. The involvement of H-NS in transcription regulation of a specific gene has only been identified

during the systematic search for transcription factors. For instance, the *csgD* (the master regulator of biofilm formation), the *ndh* gene (NADH:ubiqinone oxidoreductase), and the *gadX* gene (the stress-response regulator for acid resistance) are all under the control of more than 10 transcription factors including H-NS [4,22]. By using the newly developed PS-TF (promoter-specific transcription factor) screening system, we have identified the involvement of H-NS for regulation of a set of genes that are all under the control of multiple transcription factors [49]. These findings altogether suggest that the involvement of H-NS in transcription silencing of a number of constitutive promoters will be established once PS-TF screening is performed.

Discussion

Definition of the constitutive promoters

The pattern of genome transcription in *E. coli* is determined by controlling the utilization of a limited number of RNA polymerase [4,22]. High-throughput systems have been developed for identification of the RNA polymerase distribution within the genome. For instance, large-scale mapping of the functioning promoters *in vivo* on the *E. coli* genome has been performed by the chromatin immuno-precipitation and microarray (ChIP-chip) analysis with specific antibodies against RNA polymerase subunits [14,15]. To prevent moving RNA polymerase along DNA, *E. coli* cells were treated with rifampicin, which binds to the RpoB subunit of RNA polymerase and blocks transcription initiation, thereby fixing the initiated RNA polymerase on promoters [50]. For identification of the distribution pattern of functioning RNA polymerase, RNA-Seq analysis of high throughput sequencing of transcriptomes is becoming the method of choice [16–18]. Concomitant with the increase in the number of promoters detected under various stressful culture conditions, the variation of promoter sequence is expanding. The fluctuation of functioning promoters is attributable to the modulation of promoter selectivity of RNA polymerase by sigma factors and transcription factors with positive and negative regulatory functions [3,4].

The constitutive promoter has been considered to represent a set of promoters associated with the genes constitutively expressed *in vivo* in all circumstances. Based on this definition, however, it is practically impossible to identify the whole set of constitutive promoters of *E. coli* under various environmental conditions. At present, the whole sets of promoters determined by high-through put procedures (see above) are accumulating, but the experiments have been performed using different *E. coli* strains, under different culture conditions and using different experimental systems. In addition, even in laboratory culture conditions, it now turned clear that the steady state of cell growth does not exist and moreover, the genome expression pattern varies between individual cells within the same culture. In contrast, the whole set of constitutive promoters can be identified *in vitro* because in the case of *E. coli*, the faithful transcription can be established *in vitro* using purified RNA polymerase and pure DNA template under defined conditions. To avoid the complexity arisen from *in vivo* determination of the functioning promoters, an attempt was then made in this study to identify the whole set of the constitutive promoters using an *in vitro* system. Based on the results, we propose to revise the definition of the constitutive promoter as the promoter that is recognized and transcribed by the RNA polymerase holoenzyme alone in the absence of supporting TFs.

SELEX-chip search for the constitutive promoters

For this purpose, we used the improved method of Genetic SELEX screening [21], which has been successfully employed for identification of binding sites of a number of TFs on the *E. coli* genome [22]. As a result, the number of regulation targets markedly increased even for the TFs with known regulatory functions. For instance, the number of regulation targets increased more than 2.5 fold from 150 to 350 even for the best characterized transcription factor, CRP (cAMP receptor protein) or CAP (catabolite activator protein) [51]. This experimental system is particularly useful for short-cut estimation of the regulation targets of uncharacterized TFs including YbjK (renamed to RcdA), YcdC (renamed to RutR), YcjZ (renamed to PgrR), YdhM (renamed to NemR) and YgiP (renamed to Dan) [22]. The Genomic SELEX screening system has also been successfully employed for detection of the alteration of promoter-recognition properties of transcription factors after phosphorylation (in the case of two-component systems) or interaction with effector ligands. For instance, the selection of regulation targets of SdiA, a regulator of genes for cell division and differentiation, was found to alter differently in the presence of each of three homoserine lactone analogs, the QS signals [52].

As an extension of screening of the whole set of RpoD promoters, we have successfully performed the screening of promoters recognized by RNA holoenzymes containing minor sigma factors, RpoS, RpoH, RpoF, RpoE and FecI. In the case of RpoN sigma factor, it requires enhancer-binding proteins such as NtrC and for formation of stable RNA polymerase-promoter complexes. Results will be described elsewhere.

Physiological roles of the constitutive promoters

The genes under the control of constitutive promoters are supposed to be expressed constitutively although unnecessary genes are subject to repression by silencers (see the H-NS chapter). One short-cut interpretation is that the essential genes are under the control of constitutive promoters. After systematic deletion of the genome, the total number of essential genes in the *E. coli* genome has been minimized to generate the minimal genome consisting of 302 essential genes ([53]; also see PEC database). Among 302 essential genes, 70 (23%) were identified to be under the control of constitutive promoters. Noteworthy is that the expression of some essential genes should be under the control of constitutively expressed positive transcription factors. In fact, we have identified the presence of approximately 100 species of *E. coli* transcription factor throughout cell growth.

Marked variation of the promoter sequences listed in databases must have been arisen from several different factors: 1) the list of RpoD-dependent promoters include those recognized by RpoS (and possibly other minor sigma factors), of which the promoter recognition properties overlaps with RpoD [54,55]; 2) the list also includes promoters that are functional *in vivo* only under the support of positive TFs [56–58]; and 3) a variety of promoter-like sequences have been identified *in silico* to be promoters. In fact approximately 60% of the RpoD holoenzyme-binding sites are located inside open reading frames (see Fig. 1A). Binding of RNA polymerase on some open reading frames has been recognized [59]. The unexpected high number of RpoD holoenzyme-binding sites inside open reading frames raises a possibility of an as yet unidentified functional role(s) for the RpoD holoenzyme. These promoter-like sequences may contribute transcription initiation from internal promoters and/or blocking the migration of elongation complexes. Clustering of promoter-like sequences within the *E. coli* genome were predicted by Collado-Vides and colleagues [60] while Ozoline and colleagues proposed the presence of as many as 78 'Promoter Islands' [59,61]. These promoter-like sequences could form transcriptionally inactive

Table 3. Constitutive Promoters (Type-B Spacers) (Rightward transcription).

Map	*Left Operon*	*Left Gene*	D	RpoD	D	*Right Gene*	*Right Operon*	Promoter sequence
0.37	*sokC*	*sokC*	>	HNS	>	*nhaA*	*nhaAR*	TTAACC-17-**TCTAAT**
0.61	*dapB*	*dapB*	>		>	*carA*	*carAB*	**TTGACT**-17-CAGAAT
0.99	*uaaU*	*yaaU*	>		>	*kefF*	*kefFC*	**TTGACT**-16-TATGAC
2.27	*(mraZ- -lpxC)*	*ftsZ*	>		>	*lpxC*	*lpxC*	
2.76	*(pdhR- -lpd)*	*aceF*	>		>	*lpd*	*lpd*	TTTAAA-17-**TAAAAT**
3.55	*hrpB*	*hrpB*	>		>	*mrcB*	*mrcB*	TTGAGA-17-TGTAAC
3.61	*mrcB*	*mrcB*	>		>	*fhuA*	*fhuACDB*	TTGCGA-18-**TATTAT**
3.81	*clcA*	*clcA*	>		>	*erpA*	*erpA*	TAGATA-19-**TAGAAT**
4.33	*(bamA- -lpxA)*	*lpxD*	>		>	*fabZ*	*(bamA- --lpxA)*	TCGCCA-15-TCTCAT
4.36	*(bamA- -lpxA)*	*fabZ*	>		>	*lpxA*	*(bamA- -lpxA)*	Internal Promoter
5.41	*dinB-yafNOP*	*dinB*	>		>	*yafN*	*yafNOP*	
5.62	*(proBC)*	*proA*	>		>	*thrW*	*thrW*	**TTGACG**-15-TTTAAC
7.15	*betT*	*betT*	>	HNS	>	*yahA*	*yahA*	TTGATC-16-***TATAAT***
8.44	*yaiU*	*yaiU*	>	HNS	>	*yaiV**	*yaiV*	TTCACT-18-**TTTAAT**
8.73	*yaiI*	*yaiI*	>	HNS	>	*aroL*	*aroL-yaiA-aroM*	TCGAAA-17-**TATGAT**
9.33	*(nrdR- -pgpA)*	*ribD*	>		>	*ribE*	*(nrdR- -pgpA)*	Internal Promoter
9.79	*bloA*	*bolA*	>		>	*tig*	*tig*	TCGACT-17-***TATAAT***
9.83	*tig*	*tig*	>		>	*clpP*	*clpPX*	TTGAAA-17-CATAAC
10.08	*cof*	*cof*	>		>	*ybaO**	*ybaO*	TTGTCG-17-**TAAAAT**
10.70	*adk*	*adk*	>		>	*hemH*	*hemH*	TTATCA-15-GATATT
11.26	*(ybbAP)*	*ybbP*	>	HNS	>	*rhsD*	*rhsDC-yibH*	TTAATA-17-**TGTAAT**
11.36	*(rhsD- -ylbH)*	*ylbH*	>		>	*ybbD*	*ybbD*	
11.38	*ybbD*	*ybbD*	>		>	*ylbI*	*ylbI*	TCGTCA-19-TAAAAT
12.23	*(renD-emrE)*	*emrE*	>	HNS	>	*ybcK*	*ybcK*	GTGACC-17-TAAAAA
12.24	*ybcK*	*ybcK*	>	HNS	>	*ybcL*	*ybcLM*	GTGGCA-17-TACAAT
12.29	*ybcLM*	*ybcL*	>		>	*ybcM*	*(ybcLM)*	Internal Promoter
12.55	*tfaX*	*tfaX*	>	HNS	>	*appY**	*appY*	TTATCA-17-**TTTAAT**
13.06	*sokE*	*sokE*	>		>	*hokE*	*hokE*	
13.56	*(entcEBAH)*	*entH*	>		>	*cstA*	*cstA*	TTTACA-15-**TAAATT**
14.15	*pagP*	*pagP*	>		>	*cspE*	*cspE*	TGGACA-17-TGTACT
14.57	*ybeR-djiB*	*ybeR*	>		>	*djiB*	*(ybeR-djiB)*	Internal Promoter
15.84	*(ybfOC)*	*ybfC*			>	*ybfQ*	*ybfQ*	TTTTCA-17-AATACT
16.61	*(mngAB)*	*mngB*	>	HNS	>	*cydA*	*cydAB*	TCTACA-17-**TATATT**
17.49	*(biobFCD)*	*bioD*	>		>	*uvrB*	*uvrB*	TTGGCA-17-**TAAAAT**
17.64	*(moaABCDE)*	*moaE*	>		>	*ybhL*	*ybhL*	TGCACA-17-TATCCT
17.65	*ybhL*	*ybhL*	>	HNS	>	*ybhM*	*ybhM*	ACGACA-16-**TATAAA**
19.21	*(ybjC- -ybjN)*	*nfsA*	>		>	*rimK*	*rimK-ybjN*	
20.23	*(lolA-rarA)*	*rarA*	>		>	*serS*	*serS*	TGGCCA-17-GATAAG
20.68	*(serC-aroA)*	*aroA*	>		>	*ycaL*	*ycaL*	TTGATA-17-ATTAAT
23.37	*(efeOB)*	*efeB*	>	HNS	>	*phoH*	*phoH*	TTTATA-16-**TATATT**
24.80	*(rpmF- -fabHDG)*	*fabG*	>		>	*acpP*	*acpP-fabF*	TTGCAA-16-TACACT
24.84	*(acpP-fabF)*	*fabF*	>		>	*pabC*	*pabCG- -ycfH*	CTGCCA-15-GATAAG
25.12	*(hinT- -ycfP)*	*ycfP*	>	HNS	>	*ndh*	*ndh*	CTCACA-17-AACAAT
25.92	*(ymfTL- -ymfS)*	*ymfL*	>		>	*ymfM*	*(ymfTL- -ymfS)*	Internal Promoter
26.07	*pinE*	*pinE*	>		>	*mcrA*	*mcrA*	TTGTCG-17-ATTAAT
26.19	*ycgZ- -ymgC*	*ycgZ*	>		>	*ymgA*	*(ycgZ- -ymgC)*	Internal Promoter

Table 3. Cont.

Map	*Left Operon*	*Left Gene*	D	RpoD	D	*Right Gene*	*Right Operon*	Promoter sequence
26.20	*(ycgZ- -ymgC)*	*ariR*	>		>	*ymgC*	*(ycgZ- -ymgC)*	Internal Promoter
26.22	*(ycgZ- -ymgC)*	*ymgC*	>	HNS	>	*ycgG*	*ycgG*	TTGACG-19-TATTTT
26.27	*ymgF*	*ymgF*	>	HNS	>	*ycgH*	*ycgHI*	TTGACA-19-**TATAAG**
28.00	*ychE*	*ychE*	>	HNS	>	*oppA*	*oppABCDF*	TTAACA-17-AAGAAT
28.51	*(yciVOQ)*	*yciO*	>		>	*yciQ*	*(yciVOQ)*	Internal Promoter
28.71	*topA*	*topA*	>	HNS	>	*cysB**	*cysB*	TTCACA-15-**TATAAA**
28.84	*pgpB*	*pgpB*	>		>	*yciS*	*yciSM*	TTGATT-18-AATCAT
31.32	*(paaAB- -GHIJK)*	*paaB*	>		>	*paaC*	*(paaAB- -GHIJK)*	Internal Promoter
31.44	*(paaAB- -GHIJK)*	*paaJ*	>		>	*paaK*	*(paaAB- -GHIJK)*	Internal Promoter
31.63	*insI-2*	*insI-2*	>		>	*ydbC*	*ydbC*	**TTAACA**-17-TCGAAT
32.89	*yncH*	*yncH*	>	HNS	>	*rhsE*	*rhsE*	TTGACT-17-TATTAC
34.53	*(lsrACD- -tam)*	*lsrD*	>		>	*lsrB*	*(lsrACD- -tam)*	Internal Promoter
35.16	*ydfK*	*ydfK*		HNS	>	*pinQ*	*pinQ*	TGTACA-16-**AATAAT**
35.51	*dicF*	*dicF*	>		>	*dicB*	*dicB- -insD-intQ*	
35.98	*ynfM*	*ynfM*	>	HNS	>	*asr*	*asr*	GTCACA-18-**TGTAAT**
36.26	*(rstAB)*	*rstB*	>		>	*tus*	*tus*	TGGTCA-17-**TATAAA**
37.36	*ydhO*	*ydhO*	>		>	*sodB*	*sodB*	TTGCTA-16-AATAAG
37.49	*ydhC*	*ydhC*	>		>	*cfa*	*cfa*	CTAACA-17-TGAAAT
38.12	*rprA*	*rprA*	>		>	*ydiL*	*ydiL*	CTGATA-15-TATTGT
38.53	*aroH*	*aroH*	>	HNS	>	*ydiE*	*ydiE*	TTGATA-16-TATCAA
38.97	*yniC*	*yniC*	>		>	*ydjM*	*ydjM*	CTGAAA-17-ATTAAT
38.99	*ydjM*	*ydjM*	>		>	*ydjN*	*ydjN*	ATGACT-16-**AATAAT**
39.26	*nadE*	*nadE*	>		>	*cho*	*cho*	**TTGTCA**-15-**TTTAAT**
41.46	*holE*	*holE*	>		>	*yobB*	*yobB-exoX*	TATACA-16-CATAAC
43.96	*(yedVZ)*	*yedZ*	>		>	*zinT*	*zinT*	TTGTCA-18-**AATAAT**
44.29	*amn*	*amn*	>	HNS	>	*yeeN*	*yeeN*	TAGACG-18-***TATAAT***
44.60	*yeeP*	*yeeP*	>		>	*isrC*	*isrC*	TTGTCC-17-**TAGAAT**
44.60	*isrC*	*isrC*	>		>	*flu*	*flu*	
46.62	*(mdtA- -baeSR)*	*baeR*	>		>	*yegP*	*yegP*	CTGGCA-17-CATACT
48.45	*yeiH*	*yeiH*	>		>	*nfo*	*nfo*	
48.47	*nfo*	*nfo*	>		>	*yeiI*	*yeiI*	
49.23	*(yejLM)*	*yejM*	>		>	*proL*	*proL*	TTGCAA-16-TAGTAT
49.61	*yojO*	*yojO*	>	HNS	>	*eco*	*eco*	GCGACA-15-TATAAA
51.29	*rbn*	*rbn*	>		>	*elaD*	*elaD*	TTAAAA-18-TGTTAT
54.54	*cysZ*	*cysZ*	>		>	*cysK*	*cysK*	ATGTCA-16-TATAGA
55.21	*(yffOP)*	*yffP*	>		>	*yffQ*	*yffQR*	CTCACA-16-TATCAC
55.23	*(yffQR)*	*yffR*	>		>	*yffS*	*yffS*	
56.02	*bcp*	*bcp*	>		>	*hyfA*	*hyfAB- -GHIJR*	ATGACC-17-CAGAAT
56.02	*hyfAB- -GHIJR*	*hyfA*	>		>	*hyfB*	*(hyfAB- -GHIJR)*	Internal Promoter
57.13	*sseA*	*sseA*	>	HNS	>	*ryfA*	*ryfA*	**TTGTCA**-16-TATTGT
58.93	*bamD*	*bamD*	>		>	*raiA*	*raiA*	CTGTCA-18-TTTAGT
59.27	*nadK*	*nadK*	>		>	*recN*	*recN*	TTTACG-17-**TATAAA**
59.35	*smpB*	*smpB*	>		>	*ssrA*	*ssrA*	TGGTCA-18-**TATACT**
59.59	*(rnlAB)*	*rnlB*	>			*yfjP*	*yfjPQ*	TTGAAA-15-**TATCAT**
59.79	*yfjW*	*yfjW*	>		>	*yfjX*	*yfjXYJZ-ypjF*	TTGGCA-19-**TATAAA**
59.82	*(yfjXYJZF)*	*ypjF*	>		>	*psaA*	*psaA*	**CTGACA**-15-AACAAT

Table 3. Cont.

Map	*Left Operon*	*Left Gene*	D	RpoD	D	*Right Gene*	*Right Operon*	Promoter sequence
60.31	*ygaM*	*ygaM*	>		>	*nrdH*	*nrdHIEF*	TCAACA-18-**TATCAT**
60.46	*(proVWX)*	*proW*	>		>	*proX*	*(proVWX)*	TTATCA-16-AATAAC
60.51	*(ygaXY)*	*ygaY*	>		>	*ygaZ*	*ygaZH*	TTAAGA-17-***TATAAT***
60.92	*(srlAEBD)*	*srlD*	>		>	*gutM**	*gutM-srlRQ*	
61.59	*mutS*	*mutS*	>	HNS	>	*pphB*	*pphB*	TTAACG-17-TAAAAA
64.43	*ygeF*	*ygeF*	>	HNS	>	*ygeG*	*ygeG*	TTTAAA-17-TATCAA
64.49	*ygeI*	*ygeI*	>		>	*pbl*	*pbi*	TTGACC-16-GATACT
65.32	*yqfG*	*yqfG*	>		>	*idi*	*idi*	TTGTCG-18-AATCAT
65.82	*zapA*	*zapA*	>		>	*ssrS*	*ssrS-fau*	
65.83	*(ssrS-fau)*	*fau*	>		>	*sibC*	*sibC*	***TTGACA***-15-CCTAAT
66.49	*metK*	*metK*	>	HNS	>	*galP*	*galP*	ATAACA-18-**TATAAC**
67.93	*metC*	*metC*	>		>	*yghB*	*yghB*	TGGACA-15-**TATTGT**
68.61	*yqiC*	*yqiC*	>	HNS	>	*ygiL*	*ygiL*	TCGATA-17-**TATAAA**
69.10	*(ttdABT)*	*ttdB*	>		>	*ttdT*	*(ttdABT)*	Internal Promoter
69.42	*ebgR*	*ebgR*	>		>	*ebgA*	*ebgAC*	TTGCCG-15-TATTTT
69.55	*ygjJK*	*ygjJ*	>		>	*ygjK*	*(ygjJK)*	Internal Promoter
69.79	*aix*	*alx*	>		>	*sstT*	*sstT*	CTGACC-17-TGTCAT
69.93	*exuT*	*exuT*	>		>	*exuR**	*exuR*	TTTTCA-16-TAAACT
69.96	*exuR*	*exuR*	>		>	*yqjA*	*yqjA-mzrA*	TTGTCT-17-**TATAAA**
70.39	*tdcR*	*tdcR*	>		>	*yhaB*	*yhaBC*	TTGATA-19-GAAAAT
70.79	*(agaS--agaBCDI)*	*agaI*	>	HNS	>	*yraH*	*yraHI*	**TTGATA**-17-TCAAAT
71.84	*ispB*	*ispB*	>		>	*sfsB**	*sfsB*	TTTAGA-18-**TATAGT**
72.93	*argR*	*argR*	>	HNS	>	*yhcN*	*yhcN*	TTGAAA-18-AATAAC
73.46	*(panf-prmA)*	*prmA*	>		>	*dusB*	*dusB-fis*	GTGCCA-18-AAAAAT
75.03	*yheST*	*yheS*	>		>	*yheT*	*(yheST)*	Internal Promoter
75.35	*(nirBDC-cysG)*	*cysG*	>	HNS	>	*yhfL*	*yhfL*	TTAACG-19-***TATAAT***
75.39	*yhfL*	*yhfL*	>		>	*frlA*	*frlABCDR*	**CTGACA**-18-**TTTAAT**
76.26	*yhgF*	*yhgF*	>	HNS	>	*feoA*	*feoABC*	TTATCA-15-**TTTAAT**
77.16	*yhhZA*	*yhhZ*	>		>	*insA-6*	*insAB-6*	**TTGAAA**-17-**TTTAAT**
77.20	*(insAB-6)*	*insB6*		HNS	>	*yrhD*	*yrhD*	TAGAGA-18-**TATATT**
78.71	*slp-dctR*	*slp*		HNS	>	*dctR*	*dctR*	TTAATA-17-**TATTAT**
80.65	*yiaK*	*yiaK*	>		>	*yiaL*	*(yiaK- -sgbHUE)*	Internal Promoter
81.13	*yibA*	*yibA*	>		>	*yibJ*	*yibJ*	
81.15	*yibJ*	*yibJ*	>		>	*yibG*	*yibG*	
81.20	*yibV*	*yibV*			>	*yibU*	*yibU*	TTAACT-15-**GATAAT**
81.79	*(rfaD-waaFCL)*	*waaC*	>		>	*waaL*	*(rfaD-waaFCL)*	Internal Promoter
82.24	*yicC*	*yicC*	>		>	*dinD*	*dinD*	GTGAGA-15-**TATAAA**
82.34	*dinD*	*dinD*	>		>	*yicG*	*yicG*	TTATCA-16-AAAAAT
82.48	*xanP*	*xanP*	>		>	*yicH*	*yicH*	
85.10	*ilvLXGMEDA*	*ilvL*	>		>	*ilvX*	*(ilvLXGMEDA)*	Internal Promoter
85.43	*trxA*	*trxA*	>		>	*rhoL*	*rhoL-rho*	**TTGACT**-17-TATTAA
85.74	*(rfe- -tffT- -rffM)*	*rffM*	>		>	*yifK*	*yifK*	ATTACA-15-**TTTAAT**
87.25	*polA*	*polA*	>		>	*spf*	*spf*	CTGTCA-17-TAGAAA
87.47	*typA*	*typA*	>		>	*yihL*	*yihLM*	
88.49	*fieF*	*fieF*	>		>	*pfkA*	*pfkA*	
90.08	*(rplKAJL-rpoBC)*	*rplL*	>		>	*rpoB*	*(rplKAJL-rpoBC)*	TAGTCA-15-TGTAAG
91.98	*aphA*	*aphA*	>		>	*yibQ*	*yibQR*	

Table 3. Cont.

Map	*Left Operon*	*Left Gene*	D	RpoD	D	*Right Gene*	*Right Operon*	Promoter sequence
92.52	*(nrfABCDEFG)*	*nrfG*	>	**HNS**	>	*gltP*	*gltP*	ATGCCA-18-**TATTAT**
94.29	*ecnB*	*ecnB*	>		>	*sugE*	*sugE*	TTGAAA-16-CAAAAT
94.89	*yjeT*	*yjeT*	>		>	*purA*	*purA*	CTGAAA-19-TTTAAG
95.01	*(nsrR-rnr- -yjflJ)*	*rlmB*	>		>	*yjfl*	*(nsrR-rnr- -yjflJ)*	TTAATA-17-TGGAAT
95.35	*(rpsF-priB- -rplI)*	*rpsR*	>		>	*rplI*	*(rpsF-priB- -rplI)*	Internal Promoter
97.06	*(yjhBC)*	*yjhC*	>		>	*ythA*	*ythA*	
97.85	*fimB*	*fimB*	>	**HNS**	>	*fimE*	*fimE*	TTGTAA-17-**CATAAT**
98.12	*(uxuAB)*	*uxuB*	>		>	*uxuR**	*uxuR*	GTGCCA-17-**TATAGT**
98.51	*yjiS*	*yjiS*	>	**HNS**	>	*yjiT*	*yjiT*	TTGAGA-18-**TATAAA**
99.35	*osmY*	*osmY*	>		>	*ytjA*	*ytjA*	
99.36	*ytjA*	*ytjA*	>		>	*yjjU*	*yjjU*	
99.52	*(deoCABD)*	*deoA*	>		>	*deoB*	*(deoCABD)*	Internal Promoter
99.57	*(deoCABD)*	*deoD*	>	**HNS**	>	*yjjJ**	*yjjJ*	TTTTCA-18-TCTATT
99.57	*yjjJ*	*yjjJ*	>	**HNS**	>	*yjtD*	*yjtD*	TTGTCG-17-AATTAT
				**HNS**			**154 (b)**	**271 (a)**
				**38**			**9 TFs**	**1.76 (a/b)**
				**25%**			**17 essential**	**62 Y-genes**

Among the total of 1,075 RpoD holoenzyme-binding sites, 154 are located within type-B spacers upstream of right-side genes and downstream of lest-side genes, indicating that these promoters direct rightward transcription. The genes and operons under the control of these 154 promoters were estimated, of which 18 represent putative internal promoters. A total of 9 genes encoding transcription factors are indicated by star symbols (*). The essential genes listed in the PEC database are underlined within the operons. The promoter sequence with complete match with the canonical promoter (see Fig. 4) is shown in bold and italic while the promoter sequence with 5-out of-6 match is shown in bold. The spacers including H-NS binding sites are marked as HNS in the spacer column. Total number of genes under the control of 154 promoters were 271 (1.76 gene per promoter). Among the total of 154 RpoD holoenyme-binding sites, 38 (25%) overlap with the H-NS-binding sites.
doi:10.1371/journal.pone.0090447.t003

complexes with RNA polymerase but might contribute to increase local concentrations of RNA polymerase on the genome.

Some of the constitutive promoters with high-level of conservation of the consensus sequence are located within long spacers with long UTR sequences but lacking protein-coding sequences of reasonable sizes. In these cases, it would be worthwhile to test as yet unidentified regulatory sRNAs [62]. Noteworthy is that such long spacers including the constitutive promoters often correspond to the Promoter Islands.

The consensus sequence of constitutive promoters

The canonical model of RpoD promoters consisting of two hexanucleotide sequences, TTGACA -35 signal and TATAAT -10 signal, each being separated by 17 bp linker was originally identified using *in vitro* transcription of some model templates by purified RNA polymerase [10,11]. Here we identified the consensus sequence of as many as 669 constitutive promoters. The most significant feature of constitutive promoters is the high-level conservation of canonical TTGACA(-35)-17bp-TATAAT(-10) sequence. We also identified the roles and -35 and -10 signals and the conservation level of each base within these two hexanucleotide sequences. The promoter -35 TTGACA signal plays a key role in binding the RNA polymerase (see Fig. 4) but G at position 3 and C at position 5 are relatively less important for the constitutive promoters, suggesting that both play roles in TF-depending inducible promoters. The promoter -10 TATAAT signal plays a major role in promoter opening (see Fig. 4) in agreement with the previous proposals [63,64], but the novel finding is that all six bases of TATAAT are equally important for this -10 signal function, supporting the hypothesis that the cooperativity of energy threshold, but not interaction of individual bases with RpoD, are important factors guiding the dynamics and selectivity of promoter open complex formation [65].

Regulation of the conserved promoter

The constitutive promoters must be repressed in cases when the genes under their control are not necessary. A group of silencing proteins play roles in preventing the potentially harmful effects of uncontrolled expression of the constitutive promoters. In *E. coli*, the H-NS family proteins are the major players of anti-silencing [44,45,66]. The high-level of overlapping was observed in the distribution between the constitutive promoters (see Tables 1, 2 and 3) and the binding sites of silencer H-NS [45]. A total of 203 (30%) of the constitutive promoters were predicted to be under the control of H-NS silencer, but silencing proteins for other 70% promoters remain unidentified. The spectrum of silencing targets by H-NS should be modulated by interaction with the members of Hha/YdgT family of small-sized co-regulators [67,68]. Possible involvement of other growth condition-specific nucleoid proteins in silencing such as Dps (DNA-binding protein in starved cells) [69] and Dan (DNA-binding protein under anaerobic conditions) [32] awaits further studies.

It should be noted that the constitutive promoters are also subject to activation by positive TFs for enhanced expression of the target genes. For instance, the *csgD* gene encoding the master regulator of biofilm formation carries one of the Type-A constitutive promoters, which is controlled by as many as 20 positive and negative TFs [22,33]. The level of constitutive *csgD* promoter is higher than that of *csgB* promoters directing transcription toward opposite orientation (see Fig. 2A[b]), but

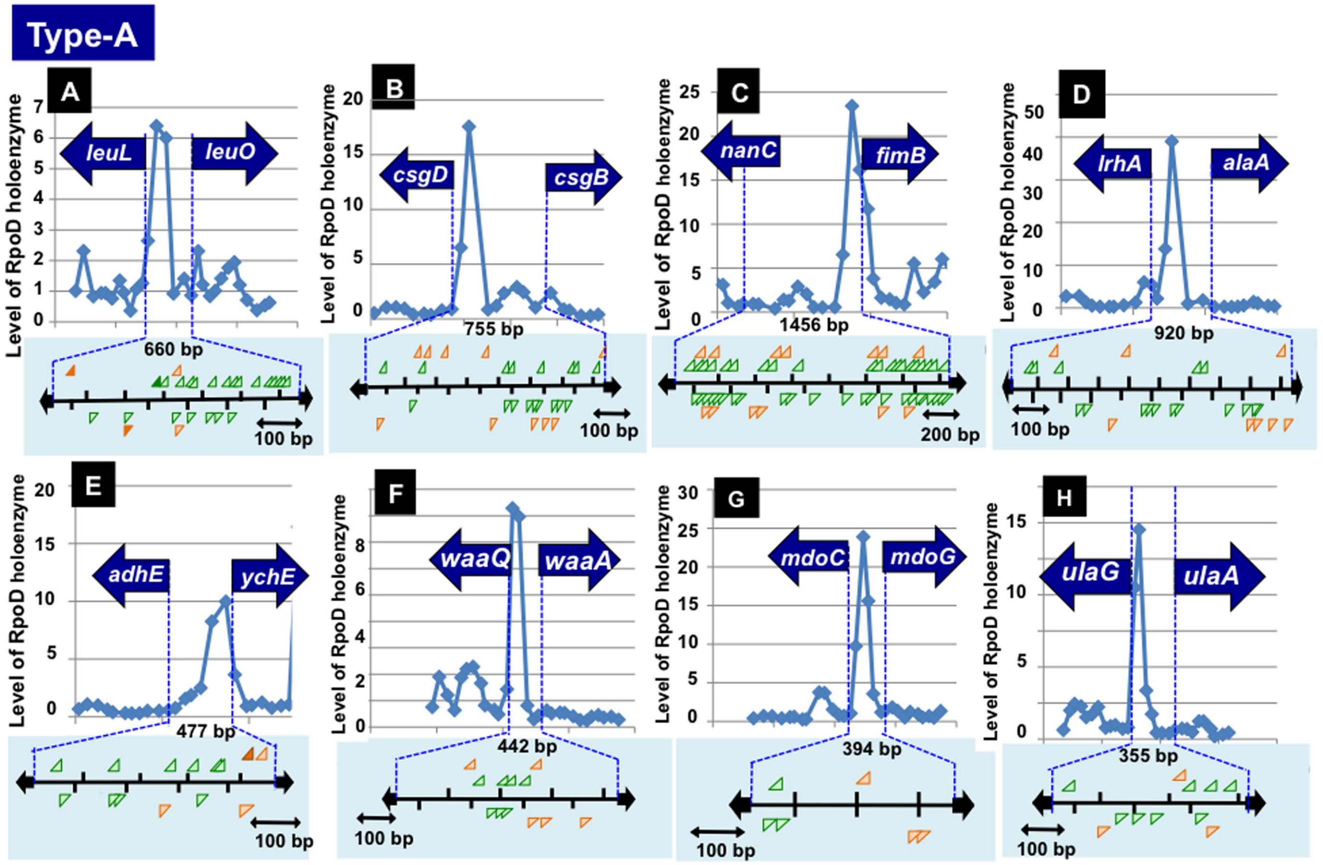

Figure 2. RpoD holoenzyme-binding peaks within type-A spacers. RpoD holoenzyme-binding peaks were identified within a total of 177 type-A spacers. Some representative patterns of RpoD holoenzyme-peaks are shown, which are located between *leuL-leuO* (a), *csgD-csgB* (b), *nanC-fimB* (c), *lrhA-alaA* (d), *adhE-ychE* (e), *waaQ-waaA* (f), *mdoC-mdoG* (g) and *ulaG-ulaA* (h). Distribution of promoter -35 (indicated by orange arrows) and -10 (indicated by green arrows) signals is shown in the panel under each SELEX pattern.
doi:10.1371/journal.pone.0090447.g002

once the regulator CsgD is produced, it antagonizes H-NS silencer and the expression of the *csgBAC* operon is markedly enhanced, leading to production of curli fimbriae for biofilm formation.

In conclusion, we classified *E. coli* promoters into the constitutive promoters recognized by RNA polymerase holoenzyme alone and the transcription factor-assisted inducible promoters. Using the information of RpoD holoenzyme-binding sites identified by Genomic SELEX screening system, we predicted a total of 669 constitutive promoters with high-level conservation of the promoter consensus sequences. This finding indicates that the majority of hitherto identified promoters represent the TF-dependent inducible promoters.

Materials and Methods

Bacterial strains and plasmids

E. coli K12 W3350 type-A [70] was used for purification of RNA polymerase and the template DNA for Genomic SELEX screening of RpoD promoters. *E. coli* BL21(DE3) was used for the expression and purification of sigma and core enzyme subunit proteins. Expression plasmids for the core enzyme subunits (pRpoD, pRpoA, pRpoB and pRpoC) and all seven sigma subunits (pRpoD, pRpoS, pRpoN, pRpoH, pRpoF, pRpoE and pFec) were constructed by ligating the respective coding sequences, which were prepared by PCR amplification of the *E. coli* K12 W3350 type-A genome DNA as template, into pET21 expression vector essentially according to the standard procedure used for expression of all seven sigma subunits and all 300 transcription factors in this laboratory [71,72].

Purification of core RNA polymerase

RNA polymerase was purified from log-phase cells of *E. coli* K-12 W3350 by the standard procedure [26]. Separation of the core and holoenzymes was performed by passing the purified RNA polymerase through P11 phosphocellulose column in the presence of 50% glycerol [26]. To remove trace amounts of the core enzyme-associated sigma factors, the purified RNA polymerase in the storage buffer containing 50% glycerol was dialyzed against the same buffer but containing 5% glycerol and fractionated by phosphocellulose column chromatography in the presence of 5% glycerol [26]. The level of remaining sigma factors was less than 0.1%, if any, as checked by immuno-staining with antibodies against each of seven sigma factors.

Purification of core and sigma subunits

The core enzyme subunits (RpoA, RpoB, RpoC and RpoZ) were expressed using the respective expression plasmids and purified by two cycles of column chromatography through DEAE (DE52) and P11 phosphocellulose [26]. Sigma subunits were expressed and purified by ion-exchange column chromatography through DE52 and P11 followed by Sephacryl S-300 gel filtration column [26]. The purified sigma and core subunit proteins were

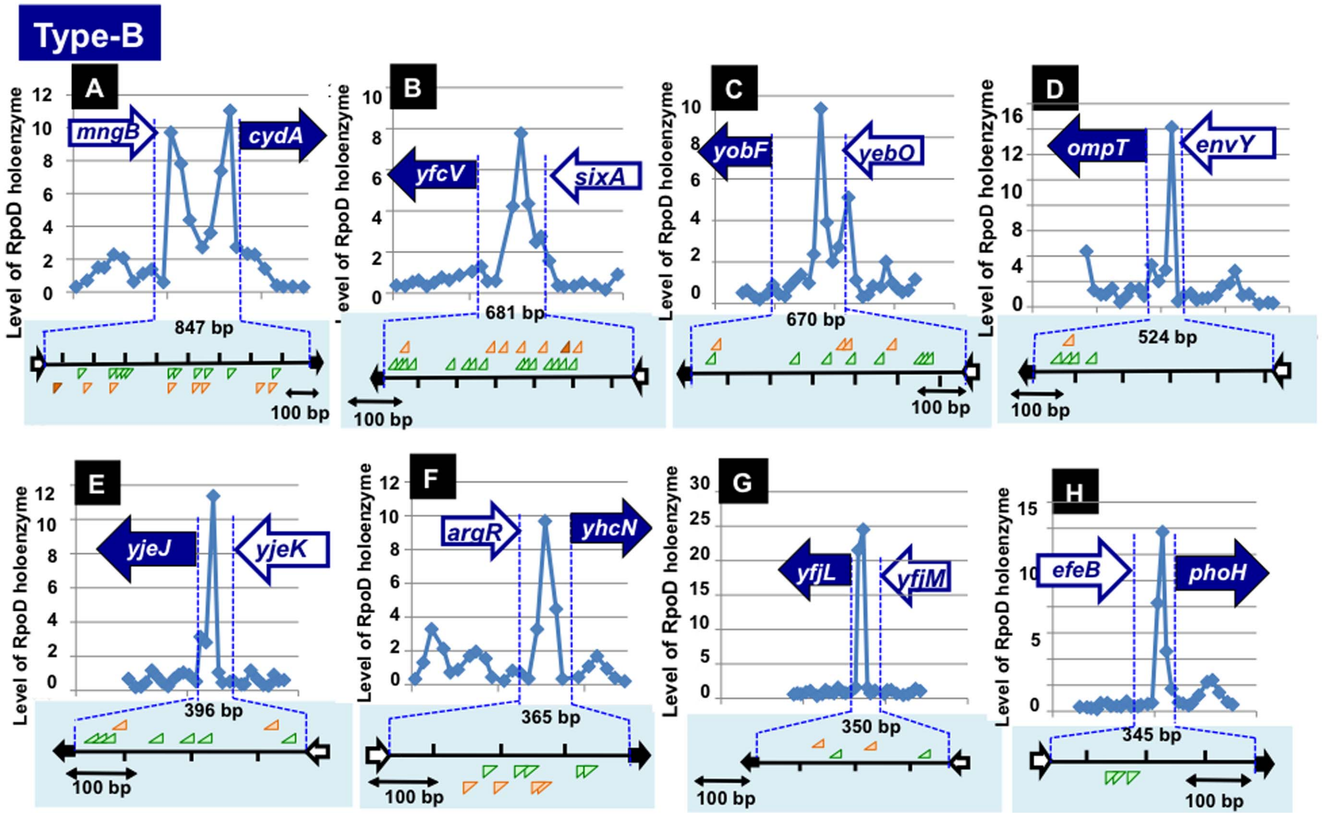

Figure 3. RpoD holoenzyme-binding peaks within type-B spacers. RpoD holoenzyme-binding peaks were identified within a total of 315 type-B spacers. Some representative patterns of RpoD holoenzyme-peaks are shown, which are located, which include the constitutive promoters for *cydA* (a), *yfcV* (b), *yobF* (c), *ompT* (d), *yjeJ* (e), *yhcN* (f), *yfjL* (g) and *phoH* (h) operons. Distribution of promoter -35 and -10 is shown below each panel.
doi:10.1371/journal.pone.0090447.g003

more than 99% pure as judged by protein staining of SDS-PAGE gels.

Preparation of antibodies

Antibodies against RpoD sigma and core enzyme subunits were produced in rabbits by injecting purified sigma proteins. The protocol for antibody production was raised following the Ethical Guidelines proposed by the Science Council of Japan and the Japanese Government, and approved by the Committee on the Ethics of Animal Experiments in the Animal Laboratory of Mitsubishi Chemical Medience Co. (Uto, Kumamoto, Japan). Antibodies against each RNA polymerase proteins were produced in two rabbits, and after examination of antibody activity using immune-blot analysis, the batch of higher activity was used in this study. Anti-RpoD, anti-RpoC used in this study did not cross-react with each other.

Genomic SELEX screening of RpoD holoenzyme-binding sequences

The genomic SELEX method was carried out as previously described [21]. A mixture of DNA fragments of the *E. coli* K-12 W3110 genome was prepared after sonication of purified genome DNA, and cloned into a multi-copy plasmid pBR322. In each SELEX screening, the DNA mixture was regenerated by PCR. For SELEX screening, 5 pmol of the mixture of DNA fragments and 10 pmol RNA polymerase RpoD holoenzyme were mixed in a binding buffer (10 mM Tris-HCl, pH 7.8 at 4°C, 3 mM magnesium acetate, 150 mM NaCl, and 1.25 mg/ml bovine serum albumin) and incubated for 30 min at 37°C. The DNA-RNA polymerase mixture was treated with anti-RpoC antibody and DNA fragments recovered from the complexes were PCR-amplified and subjected to next cycle of SELEX for enrichment of RNA polymerase-bound DNA fragments.

For SELEX-chip analysis, DNA samples were isolated from the DNA-protein complexes at the final state of SELEX, PCR-amplified and labeled with Cy5 while the original DNA library was labeled with Cy3. The fluorescent labeled DNA mixtures were hybridized to a DNA microarray consisting of 43,450 species of 60 b-long DNA probe, which are designed to cover the entire *E. coli* genome at 105 bp interval (Oxford Gene Technology, Oxford, UK) [27–29]. The fluorescent intensity of test sample at each probe was normalized with that of the corresponding peak of original library. After normalization of each pattern, the Cy5/Cy3 ratio was measured and plotted along the *E. coli* genome.

Mixed transcription assay *in vitro*

The promoter sequence of 195 bp-long *lacUV5* template and its 3′ truncated 175 bp-long DNA was modified to the consensus sequence, TTGACA(-35)-17 bp-TATAAT(-10), each producing run-off transcripts of 42 and 22 nucleotides in length, respectively. Starting from these DNA fragments with the ideal promoter, the complete set of variant consensus promoters, each containing one base substitution at all positions of the hexanucleotide sequences of promoter -35 and -10, was prepared as described previously [23]. The test promoters of variant consensus promoters directed the synthesis of 42 b-long RNA while the reference template containing the original consensus promoter directed the synthesis

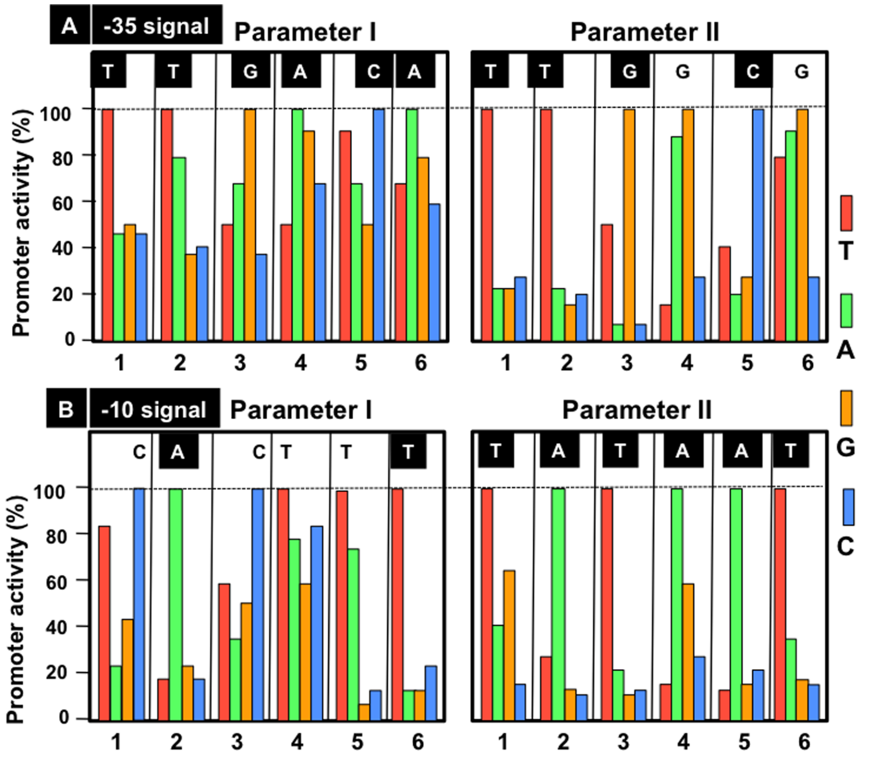

Figure 4. Determination of the consensus sequence of constitutive promoters using the *in vitro* mixed transcription system. Mixtures of equal amounts of 195 bp-long template containing the ideal promoter of complete consensus sequence and 175 bp-long mutant template, each carrying one base substitution, were subjected to the *in vitro* mixed transcription [24,25]. After preincubation for 0.5, 1.0, 2.5, 5.0, 7.5, 10 and 15 min, a mixture of substrates and heparin was added and RNA synthesis was allowed for 10 min. The final level of RNA synthesis represents the level of RpoD holoenzyme binding (parameter I) while the rate of open complex formation (parameter II) was determined as a reciprocal of the time required to reach the plateau level. For each set of four promoters with mutations at the same position, the promoter activities are shown as the values relative to the promoter with the highest activity.
doi:10.1371/journal.pone.0090447.g004

of 22 b-long RNA. The *in vitro* mixed transcription was performed under the standard single-round reaction conditions in the presence of [^{32}P]UTP as a labeled substrate [23–25]. In brief, a mixture of these two templates was preincubated with RpoD holoenzyme for various times (0.5, 1.0, 2.5, 5.0, 10, 15 and 30 min) at 37°C in the standard transcription assay buffer, and then a mixture of substrates and heparin was added to allow a single-round transcription for 15 min. Heparin inactivates RNA polymerase that was not involved in open complex formation. RNA products were separated by electrophoresis on 10% PAGE containing 8.3 M urea. The amount of RNA was determined by measuring the intensity of ^{32}P radioactivity and corrected for the U content of each transcript.

Promoter sequence analysis

The complete genome sequence of *E. coli* K-12 MG1655 (U00096.2) was used for the promoter sequence analysis. The perl scripts for finding the candidate sequences of promoters include the following functions: 1) extraction of every 6 bases in the genome sequences with sliding 1 bp; 2) comparison of the 6 base sequences with TTGACA (-35) and calculation of the score by setting one match scoring 1 point, one mis-match scoring 0 point, and without gap; 3) comparison of the 6 bases with TATAAT (-10) under the same conditions; and 4) extraction of a pair of -35 and -10 hexanucleotide sequences by setting the best-match score of 12 (6 at -35 and at -10 signal); 6) extraction of a pair of -35 and -10 signals with a spacer length of 17 plus/minus 2, giving the score of +3 for 17 bp, +2 for 16 and 18 bp; and +1 for 15 and 19 bp, respectively. Thus, the maximum score for the best-match promoter in both -35 and -10 sequences separated by 17 bp spacer is 15.

The Logo pattern analysis of promoter sequences was performed using the established sequence logo generator [73].

Supporting Information

Table S1 RNA polymerase RpoD holoenzyme-binding sites on the *E. coli* genome. A total of 1,075 RpoD holoenzyme-binding sites were identified within spacers on the entire *E. coli* K-12 W3110 genome. The binding sites identified within intergenic spacers were classified into Type-A, Type-B and Type-C (see Fig. 2 and Table 1). The constitutive promoters were predicted based on the location of the RpoD holoenzyme-binding sites. [S1A] Promoters within type-A spacers. The genes under the constitutive promoters on left- and right-sides of type-A intergenic spacers and their functions are described. The genes shown under filled background encodes DNA-binding transcription factors (TFs), each regulating a set of target genes. The spacers under grey background contain two distinct peaks of holoenzyme binding. The positions of these genes on the genome are described on the left-end column while the levels of SELEX-peaks relative to the highest peak are described on the right-end column. [S1B] Promoters within type-B spacers. The genes under the constitutive promoters on either left- or right-sides of type-B intergenic spacers and their functions are described. The positions of these genes on

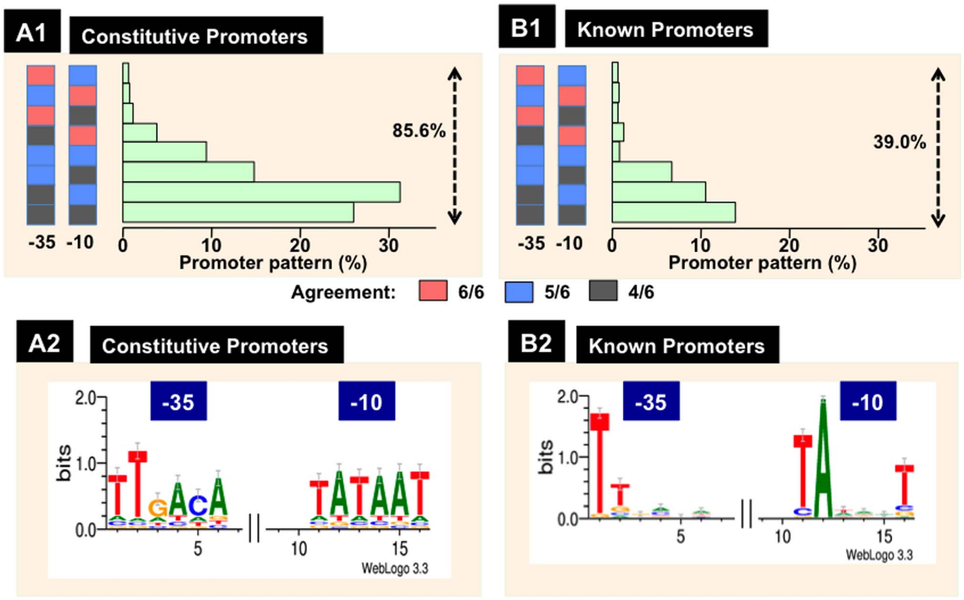

Figure 5. The composition of constitutive promoters. [A] The known consensus sequences of RpoD-dependent promoter, TTGAAC (-35) and TATAAT (-10) separated by 17 plus/minus 2 bp, were searched for all type-A (177) and type-B (315) spacers (see Experimental Procedure for the analysis method). Most of the constitutive promoters carry high-levels of the consensus sequence as listed in Table 1. The composition of promoter -35 and -10 sequence was classified into 8 groups based on the conservation level of consensus sequences. About 89% of type-A promoters and 82% of type-B promoters (or 86% of A- plus B-type promoters) contain the sequence higher than 4/6 agreement with the consensus sequence at both -35 or -10 positions (A1), while only 39% of a total of 582 known promoters carries this level of consensus sequences (B1). [B] The whole set of constitutive promoter sequences were subjected to Logo analysis [73]. The Logo patterns of -35 and -10 sequences are essentially the same among the constitutive promoters within type-A and type-B spacers. The Logo pattern of the whole set of constitutive promoters was compared with the Logo pattern generated using the total of 582 experimentally identified promoters [19,20]. The contribution of each base of the consensus -35 and -10 sequences is significantly different between the constitutive promoters and the set of known promoters.
doi:10.1371/journal.pone.0090447.g005

the genome are described on the left-end column while the levels of SELEX-peaks relative to the highest peak are described on the right-end column. The genes shown under black background encodes DNA-binding TFs. The spacers under grey background contain two distinct peaks of holoenzyme binding.
(PDF)

Table S2 Operons under the control of the constitutive promoters. Operons under the control of constitutive promoters are listed on the operon columns. Promoters shown by shaded background represent those listed in RegulonDB and EcoCyc databases. Spacers containing H-NS binding sites are indicated by HNS. [S2A] Operons under the control of constitutive promoters within type-A spacers. The constitutive promoters identified within type-A spacers direct bidirectional transcription of the operons indicated on left- or right-operon columns. The map positions of the promoter-proximal genes are indicated on the left- and right-end columns. [S2B] Operons under the control of constitutive promoters within type-B spacers. The constitutive promoters identified within type-B spacers direct transcription toward one direction.
(PDF)

Author Contributions

Conceived and designed the experiments: AI TS. Performed the experiments: TS AI. Analyzed the data: YY TS AI. Contributed reagents/materials/analysis tools: AI. Wrote the paper: AI. Supervised the project: AI KT.

References

1. Gruber TM, Gross CA (2003) Multiple sigma subunits and the partitioning of bacterial transcription space. Annu Rev Microbiol 57: 441–466.
2. Paget MSB, Helmann JD (2003) The sigma-70 family of sigma factors. Genome Biol 4: 203.
3. Ishihama A (2000) Functional modulation of *Escherichia coli* RNA polymerase. Annu Rev Microbiol 54: 499–518.
4. Ishihama A (2010) Prokaryotic genome regulation: Multi-factor promoters, multi-target regulators and hierarchic networks. FEMS Microbial Rev 34: 628–645.
5. Gourse RL, Ross W, Rutherford ST (2006) General pathway for turning on promoters transcribed by RNA polymerase containing alternative sigma factors. J Bacteriol 188: 4589–4591.
6. Lonetto M, Gribskov M, Gross CA (1992) The sigma-70 family: sequence conservation and evolutionary relationships. J Bacteriol 174: 3843–3849.
7. Saecker RM, Record MT Jr, Dehaseth PL (2011) Mechanism of bacterial transcription initiation: RNA polymerase-promoter binding, isomerization to initiation competent open complexes. J Mol Biol 412: 754–771.
8. Burgess RR, Antthony L (2001) How sigma docks to RNA polymerase and what sigma does. Curr Opin Microbiol 4: 126–131.
9. Young BA, Gruber TM, Gross CA (2004) Minimal machinery of RNA polymerase holoenzyme sufficient for promoter melting. Science 303: 1382–1384.
10. Pribnow D (1975) Bacteriophage T7 early promoters: nucleotide sequences of two RNA polymerase binding sites. J Mol Biol 15: 419–443.
11. Siebenlist U, Simpson RB, Gilbert W (1980) *E. coli* RNA polymerase interacts homologously with two different promoters. Cell 20: 269–281.
12. Busby S, Ebright RH (1994) Promoter structure, promoter recognition, and transcription activation in prokaryotes. Cell 79: 743–746.
13. Gralla JD (1996) Activation and repression of *E. coli* promoters. Curr Opin Genet Dev 6**:** 526–530.
14. Herring CD, Raffaelle M, Allen TE, Kanin EI, Landick R, et al. (2005) Immobilization of *Escherichia coli* RNA polymerase and location of binding sites by use of chromatin immunoprecipitation and microarrays. J Bacteriol 187: 6166–6174.

15. Mooney RA, Davis SE, Peters JM, Rowland JL, Ansari AZ, et al. (2009) Regulator trafficking on bacterial transcription units *in vivo*. Mol Cell 33: 97–108.
16. Cho BK, Zengler K, Qiu Y, Park YS, Knight EM, et al. (2009) The transcriptome unit architecture of the *Escherichia coli* genome. Nat Biotechnol 27: 1043–1049.
17. Raghavan R, Sage A, Ochman H (2011) Genome-wide identification of transcription start sites yields a novel thermosensing RNA and new cyclic AMP receptor protein-regulated genes in *Escherichia coli*. J Bacteriol 193: 2871–2874.
18. Ginnoukos G, Ciulla DM, Huang K, Haas BJ, Izard J, et al. (2012) Efficient and robust RNA-seq process for cultured bacteria and complex community transcritomes. Genome Biol 13: r23.70.
19. Salgado H, Peralte-Gil M, Gama-Costro S, Santos-Zavaleta A, Muniz-Rascado L, et al. (2013) RegulonDB v.8.0; omics data sets, evolutionary conservation, egulatory phrases, cross-validated gold standards and more. Nucleic Acids Res 41: D203–213.
20. Keseler IM, Collado-Vides J, Santoz-Zavaleta A, Perelta-Gil M, Gama-Castoro S, et al. (2011) EcoCyc: a comprehensive database of *Escherichia coli* biology. Nucleic Acids Res 39: D583–D590.
21. Shimada T, Fujita N, Maeda M, Ishihama A (2005) Systematic search for the Cra-binding promoters using genomic SELEX. Genes Cells 10: 907–918.
22. Ishihama A (2012) Prokaryotic genome regulation: Multi-factor promoters and multi-target regulators. Proc Jpn Acad, Ser B, Phys Biol Sci 88: 485–508.
23. Kobayashi M, Nagata K, Ishihama A (1990) Promoter selectivity of *Escherichia coli* RNA polymerase: Effect of base substitution in the promoter -35 region on promoter strength. Nucleic Acids Res 18: 7346–7372.
24. Kajitani M, Ishihama A (1983) Determination of the promoter strength in the mixed transcription system, II. Promoters of ribosomal RNA, ribosomal protein S1 and *recA* protein operons from *Escherichia coli*. Nucleic Acids Res 11: 3873–3888.
25. Kajitani M, Ishihama A (1983) Determination of the promoter strength in the mixed transcription system, I. Promoters of lactose, tryptophan and ribosomal protein L10 operons from *Escherichia coli*. Nucleic Acids Res 11: 671–688.
26. Fujita N, Ishihama A (1996) Reconstitution of RNA polymerase. Meth Enzymol 273: RNA Polymerase and Associated Factors, Part A, Adhya Sed., pp. 121–130, Academic Press, New York, 1996.
27. Ellington AD, Szostak JW (1990) *In vitro* selection of DNA molecules that bind specific ligands. Nature 346: 818–822.
28. Singer BS, Shtatland T, Brown D, Gold L (1997) Libraries for genomic SELEX. Nucleic Acids Res 25: 781–786.
29. Tuerk C, Gold L (1990) Systematic evolution of ligands by exponential enrichment: RNA ligands to bacteriophage T4 DNA polymerase. Science 249: 505–510.
30. Shimada T, Ishihama A, Busby SJW, Grainger DC (2008) The *Escherichia coli* RutR transcription factor binds at targets within genes as well as intergenic regions. Nucleic Acids Res 36: 3950–3955.
31. Shimada T, Hirao K, Kori A, Yamamoto K, Ishihama A (2007) RutR is the uracil/thymine-sensing master regulator of a set of genes for synthesis and degradation of pyrimdines. Mol Microbiol 66: 744–779.
32. Teramoto J, Yoshimura S, Takeyasu K, Ishihama A (2010) A novel nucleoid protein of *Escherichia coli* induced under anaerobiotic growth conditions. Nucleic Acids Res 38: 3605–3618.
33. Ogasawara H, Yamada K, Kori K, Yamamoto K, Ishihama A (2010) Regulation of the *Escherichia coli csgD* promoter: interplays between five transcription factors. Microbiology 156: 2470–2483.
34. Cotter PA, Davie S, Gunsalus RP (1992) The effect of iron limitation on expression of the aerobic and anaerobic electron transport pathway genes in *Escherichia coli*. FEMS Microbiol Lett 79: 227–232.
35. Ramseier TM, Chien SY, Saier MH (1996) Cooperative interaction between Cra and Fnr in the regulation of the *cydAB* operon of *Escherichia coli*. Curr Microbiol 33: 270–274.
36. Liu X, de Wulf P (2004) Probing the ArcA-P modulon of *Escherichia coli* by whole genome transcriptional analysis and sequence recognition profiling. J Biol Chem 279: 12588–12597.
37. Fukuda R, Nagasawa-Fujimori H (1983) Mechanism of the rifampicin induction of RNA polymerase beta and beta-prime subunit synthesis in *Escherichia coli*. J Biol Chem 2558: 2720–2728.
38. Harley CB, Reynolds RP (1987) Analysis of *E. coli* promoter sequence. Nucleic Acids Res 15: 2343–2361.
39. Huerta AM, Collado-Vides J (2003) Sigma 70 promoters in *Escherichia coli*: specific transcription in dense regions of overlapping promoter-like signals. J Mol Biol 333: 261–278.
40. Mitchell JE, Zheng D, Busby SJ, Minchin S (2003) Identification and analysis of 'extended -10' promoters in *Escherichia coli*. Nucleic Acids Res 31: 4689–4695.
41. Collado-Vides J, Magasanik B, Gralla JD (1991) Control site location and transcription regulation in *Escherichia coli*. Microbiol Rev 55: 371–395.
42. Ishihama A (2009) The nucleoid: an overview. *EcoSal – Escherichia coli* and *Salmonella*: Cellular and Molecular Biology (Boek A, Curtiss R III, Kaper JB, Karp PD, Neidhardt FC, Nystrom T, Slauch JM, Squires CL, UsseryD, eds). ASM Press, Washington, DC.
43. Goransson M, Sonden B, Nilsson P, Dagberg B, Forsman K, et al. (1990) Transcriptinal silencing and thermoregulation of gene expression in *Escherichia coli*. Nature 344: 682–684.
44. Navarre WW, Porwollik S, Wang Y, McClelland M, Libby SJ, et al. (2006) Selective silencing of foreign DNA with low GC content by the H-NS protein in *Salmonella*. Science 313: 236–238.
45. Shimada T, Bridier A, Briandet R, Ishihama A (2011) Novel roles of LeuO in transcription regulation in *E. coli*: Antagonistic interplay with the universal silencer H-NS. Mol Microbiol 82: 376–397.
46. Dame RT, Wyman C, Goosen N (2001) Structural basis for preferential binding of H-NS to curved DNA. Biochimie 83: 231–234.
47. Gordon BR, Li Y, Cote A, Weirauch MT, Ding P, et al. (2011) Structural basis for recognition of AT-rich DNA by unrelated xenogeneic silencing proteins. Proc Natl Acad Sci USA 28: 10690–10695.
48. Lim CJ, Lee SY, Kenney LJ, Yan J (2012) Nucleoprotein filament formation is the structural basis for bacterial protein H-NS gene silencing. Sci Rep 2: 509.
49. Shimada K, Ogasawara H, Yamada K, Shimura M, Kori A, et al. (2013) Screening of promoter-specific transcription factors: multiple regulators for the *sdiA* gene involved in cell division control and quorum sensing. Microbiogy 159: 2501–2512.
50. Campbell EA, Muzzin O, Chlenov M, Sun JL, Olson CA, et al. (2002) Structure of the bacterial RNA polymerase promoter specificity sigma subunit. Mol Cell 9: 527–539.
51. Shimada T, Fujita N, Yamamoto K, Ishihama A (2012) Novel roles of cAMP receptor protein (CRP) in regulation of transport and metabolism of carbon sources. PLoS ONE 6: e20081.
52. Shimada T, Shimada K, Matsui M, Kitai Y, Igarashi J, et al. (2014) Roles of cell division control factor SdiA: Recognition of quorum sensing signals and modulation of transcription regulation targets. Genes Cells, in press.
53. Yamazaki Y, Niki Y, Kato J (2008) Profiling of *Escherichia coli* chromomsome database. Methods Mol Biol 416: 385–389.
54. Tanaka K, Kusano S, Fujita N, Ishihama A (1995) Promoter determinants for *Escherichia coli* RNA polymerase holoenzyme containing sigma-38 (the *rpoS* gene product). Nucleic Acids Res 23: 827–734.
55. Hengge-Aronis R (2002) Stationary phase gene regulation: what makes an *Escherichia coli* promoter sigma S-selective? Curr Opin Microbiol 5: 591–595.
56. Ishihama A (1992) Role of the RNA polymerase alpha subunit in tanscription activation. Mol Microbiol 6: 3283–3288.
57. Ishihama A (1993) Protein-protein communication within the transcription apparatus. J Bacteriol 175: 2483–2489.
58. Busby S, Ebright RH (1999) Transcription activation by catabolite activator protein. J Mol Biol 293: 199–213.
59. Panyukov VV, Ozoline ON (2013) Promoters of *Escherichia coli* versus Promoter Islands: Function and structure comparison. PLoS one 8: e62601.
60. Huerta AM, Collado-Vides J (2003) Sigma 70 promoters in *Escherichia coli*: specific transcription in dense regions of overlapping promoter-like signals. J Mol Biol 333: 261–278.
61. Shavkunov KS, Masulis IS, Tutukina MN, Deev AA, Ozoline ON (2009) gains and unexpected lessons from genome-scale promoter mapping. Nucleic Acids Res 37: 4919–4931.
62. Gottesman S, Storz G (2011) Bacterial small RNA regulators: versatile roles and reapidly evolving variations. Cold Spring Harb Perspect Biol 3: a003798.
63. Burgess RR, Anthony L (2001) How sigma docks to RNA polymerase and what sigma does. (2001) Curr Opn Microbiol 4: 126–131.
64. Singh SS, Typas A, Hengge R, Grainger DC (2011) *Escherichia coli* sigma 70 senses sequence and conformation of the promoter spacer region. Nucleic Acids Res 39: 5109–5118.
65. Mekler V, Severinov K (2013) Cooperativity and interaction energy threshold effects in recognition of the -10 promoter element by bacterial RNA polymerase. Nucleic Acids Res 41: 7276–7285.
66. Oshima T, Ishikawa S, Kurokawa K, Aiba H, Ogasawara N (2006) *Escherichia coli* histone-like protein H-NS preferentially binds to horizonally acquired DNA in association with RNA polymerase. DNA Res 13: 141–153.
67. Vivero A, Banos RC, Mariscotti JF, Oliveros JC, Carcia-del Portillo F, et al. (2008) Modulation of horizonatally acquired genes by the Hha-YdgT proteins in *Salmonella enterica* serovar Typhimurium. J Bactriol 190: 1152–1156.
68. Ali SS, Whitney JC, Stevenson J, Robinson H, Howell PL (2013) Structural insights into the regulation of foreign genes in *Salmonella* by the Hha/H-NS complex. J Biol Chem 288: 13356–13369.
69. Almron M, Link AJ, Furlon D, Kolter R (1992) A novel DNA-binding protein with regulatory and protective roles in starved *Escherichia coli*. Genes Dev 6: 2648–2654.
70. Jishage M, Ishihama A (1997) Variation in RNA polymerase sigma subunit composition within different strains of *Escherichia coli* W3110. J Bacteriol 179: 959–963.
71. Maeda H, Fujita N, Ishihama A (2000) Competition among seven *Escherichia coli* sigma subunits: relative binding affinity to the core RNA polymerase. Nucleic Acids Res 28: 3497–3503.
72. Igarashi K, Ishihama A (1991) Bipartite functional map of the *E. coli* RNA polymerase alpha subunit: Involvement of the C-terminal region in transcription activation by cAMP-CRP. Cell 65: 1015–1022.
73. Crooks GE, Hon G, Chandonia JM, Brenner SE (2004) WebLogo: a sequence logo generator. Genome Res 14: 1188–1190.
